# Supplementary material for: The INO80 chromatin remodeller facilitates DNA damage bypass via postreplicative gap repair
Source: EMBO J. 2025 Oct 13;44(22):6626–48. doi: 10.1038/s44318-025-00580-4 (PMC12624141; doi:10.1038/s44318-025-00580-4)
Supplement: Supplementary file 1 — Appendix [file 44318_2025_580_MOESM1_ESM.pdf]

Appendix for

The INO80 chromatin remodeller facilitates DNA damage bypass via postreplicative gap repair

Table of Contents

Appendix Table S1. *Saccharomyces cerevisiae* strains used in this study ________ page 2

Appendix Table S2. Plasmids used in this study ________ page 4

Appendix Table S3. Oligonucleotides used in this study ________ page 5

Appendix References ________ page 6

Appendix Table S1. *Saccharomyces cerevisiae* strains used in this study

Name	ID	Genotype	Figures	Reference
DF5a (*WT* a)	003	Mata, *his3-Δ200, leu2-3,2-112, lys2-801, trp1-1(am), ura3-52*	1A-B, EV1A-C	(Finley *et al*, 1987)
arp8Δ	3445	DF5a *arp8Δ::HIS3MX6*	1A-B	This study
rad18Δ	162	DF5a *rad18Δ::TRP1*	1A, EV1B	(Ulrich & Jentsch, 2000)
ubc13Δ	345	DF5a *ubc13Δ::HIS3*	1A-B, EV1C	(Ulrich & Jentsch, 2000)
tlsΔ	1876	DF5a *rev1Δ::URA3, rev3Δ::kanMX4, rad30Δ::HIS3*	1A	(Saugar *et al*, 2012)
pol32Δ	2034	DF5a *pol32Δ::kanMX4*	1B, EV1C	(Saugar *et al*, 2012)
rad18Δ arp8Δ	5526	DF5a *rad18Δ::TRP1, arp8Δ::natNT2*	1A	This study
ubc13Δ arp8Δ	5527	DF5a *ubc13Δ::HIS3, arp8Δ::natNT2*	1A	This study
tlsΔ arp8Δ	5528	DF5a *rev1Δ::URA3, rev3Δ::kanMX4, rad30Δ::HIS3, arp8Δ::natNT2*	1A	This study
pol32Δ arp8Δ	5529	DF5a *pol32Δ::kanMX4, arp8Δ::natNT2*	1B	This study
ubc13Δ pol32Δ	3964	DF5a *pol32Δ::kanMX4, ubc13Δ::natNT2*	1B, EV1C	(García-Rodríguez *et al*, 2018b)
W303 (*WT* a)	2821	Mata, *RAD5+, leu2-3-112, trp1-1, can1-100, ura3-1, ade2-1, his3-11-15*	1C, EV1D	(García-Rodríguez *et al*, 2018b)
W303 *arp8Δ*	5293	W303 *arp8Δ::HIS3MX6*	1C	This study
W303 *ubc13Δ*	4044	W303 *ubc13Δ::natNT2*	1C, EV1D	(García-Rodríguez *et al*, 2018b)
W303 *smc6-56*	3753	W303 *smc6-56-13myc::kanMX*	1C, EV1D	(García-Rodríguez *et al*, 2018b)
W303 *smc6-56 arp8Δ*	5530	W303 *smc6-56-13myc::kanMX, arp8Δ::natNT2*	1C	This study
W303 *smc6-56 ubc13Δ*	4039	W303 *smc6-56-13myc::kanMX, ubc13Δ::natNT2*	1C, EV1D	(García-Rodríguez *et al*, 2018b)
INO80$^{AID*-FLAG}$	3438	DF5a *his3-Δ200::pRS303-ADH-AFB2(HIS3), INO80*$^{AID*-FLAG}$*::hphNT1*	EV1A-C	This study
INO80$^{AID*-FLAG}$ *pol32Δ*	4059	DF5a *INO80*$^{AID*-FLAG}$, *pol32Δ::kanMX4*	EV1C	This study
INO80$^{AID*-FLAG}$ *ubc13Δ*	5126	DF5a *INO80*$^{AID*-FLAG}$, *ubc13Δ::natNT2*	EV1C	This study
INO80$^{AID*-FLAG}$ *pol32Δ ubc13Δ*	5127	DF5a *INO80*$^{AID*-FLAG}$ *pol32Δ, ubc13Δ::natNT2*	EV1C	This study
ARP5$^{AID*-FLAG}$	4740	DF5a *his3-Δ200::pRS303-ADH-AFB2(HIS3), ARP5*$^{AID*-FLAG}$*::hphNT1*	EV1A	This study
W303 *INO80*$^{AID*-FLAG}$	5122	W303 *his3-11,15::pRS303-ADH-AFB2(HIS3), INO80*$^{AID*-FLAG}$*::hphNT1*	EV1D	This study
W303 *INO80*$^{AID*-FLAG}$ *smc6-56*	5123	W303 *smc6-56, his3-11,15::pRS303-ADH-AFB2(HIS3), INO80*$^{AID*-FLAG}$*::hphNT1*	EV1D	This study
TET-RAD18	2221	DF5a *kanMX4::tTA-TetO*$_7$*-RAD18, leu2-3,2-112::TetR'-SSN6(LEU2)*	EV1A	(Daigaku *et al*, 2010)
TET-RAD18 AFB2	5062	*TET-RAD18, his3-Δ200::pRS303-ADH-AFB2(HIS3)*	N/A	This study
TET-RAD18 ARP5$^{AID*-FLAG}$	5065	*TET-RAD18 AFB2, ARP5*$^{AID*-FLAG}$*::hphNT1*	EV1A	This study
TET-RAD18 INO80$^{AID*-FLAG}$	5063	*TET-RAD18 AFB2, INO80*$^{AID*-FLAG}$*::hphNT1*	3F, EV1A, EV3D	This study
TET-RAD18 BrdU-inc (URA) INO80$^{AID*-FLAG}$	3452	*TET-RAD18 INO80*$^{AID*-FLAG}$, *ura3-52::BrdU-inc(URA3)*	3K, EV3G	This study
TET-RAD18 INO80$^{AID*-FLAG}$ *BrdU-inc (TRP)*	5086	*TET-RAD18 INO80*$^{AID*-FLAG}$, *trp1-1(am)::BrdU-inc(TRP1)*	3N	This study
TET-RAD18 RFA1^{GFP}	3436	*TET-RAD18, RFA1*^{GFP}*::HIS3MX6*	EV3F	This study

TET-RAD18 AFB2 RFA1^{GFP}	5221	*TET-RAD18, trp1-1(am)::YIp204-ADH-AFB2, RFA1*^{GFP}*::HIS3MX6*	EV3F	This study
TET-RAD18 INO80$^{AID*-FLAG}$ *RFA1*^{GFP}	5139	*TET-RAD18 INO80*$^{AID*-FLAG}$*, RFA1*^{GFP}*::natNT2*	2B, 2D, 3G, 3L-M, EV2D-E, EV3E, EV3H, 4E, EV4A-D	This study
TET-RAD18 BrdU-inc (URA) INO80$^{AID*-FLAG}$ ^{His}*POL30*	3466	*TET-RAD18 INO80*$^{AID*-FLAG}$ *BrdU-inc(URA), trp1-1(am)::YIp211-P30-*^{His}*POL30(TRP1), pol30Δ::natNT2*	2C, 3O	This study
S288C (*WT* a)	2078	GA-4563, *trp1-1, leu2D0, lys2D0, ura3-1::TKx7-URA3, his3d1::hENT1:HSV-TK-HIS3*	N/A	(Shimada *et al*, 2008)
S288C ^{His}*POL30*	2960	S288C Mata *trp1-1, leu2Δ0::YIp128-P30-*^{His}*POL30(LEU2), lys2Δ0, ura3-1::TK×7(URA3), his3Δ1::hENT1:HSV-TK(HIS3)*	EV2B	This study
S288C ^{His}*POL30 ino80Δ*	2962	S288C ^{His}*POL30, ino80Δ::kanMX6*	EV2B	This study
S288C ^{His}*POL30 arp8Δ*	2961	S288C ^{His}*POL30, arp8Δ::kanMX6*	EV2B	This study
RFA1^{GFP} *AFB2*	3506	DF5a *trp1-1(am)::YIp204-ADH-AFB2(TRP1), RFA1*^{GFP}*::HIS3MX6*	N/A	This study
ARP8$^{AID*-FLAG}$ *RFA1*^{GFP}	3530	*RFA1*^{GFP} *AFB2, ARP8*$^{AID*-FLAG}$*::hphNT1*	4B-C	This study
ARP5$^{AID*-FLAG}$ *RFA1*^{GFP}	3531	*RFA1*^{GFP} *AFB2, ARP5*$^{AID*-FLAG}$*::hphNT1*	4B-C	This study
NHP10$^{AID*-FLAG}$ *RFA1*^{GFP}	3532	*RFA1*^{GFP} *AFB2, NHP10*$^{AID*-FLAG}$*::hphNT1*	4B-C	This study
BrdU×7 INO80$^{AID*-FLAG}$	4739	W303 *ura3-1::GPD-TK, pRS415-hENT1(LEU2), trp1-1::YIp204-ADH-AFB2(TRP1), INO80*$^{AID*-FLAG}$*::hphNT1*	3B-D, EV3A-C	This study
W303 *INO80*^{FLAG}	5577	W303 *INO80*^{5FLAG}*::hphMX6*	3H	This study
W303 *INO80*$^{AID*-FLAG}$	5568	W303 *his3-11-15::pRS303-ADH-AFB2(HIS3), INO80*$^{AID*-FLAG}$*::hphNT1*	3H	This study
W303 *cdc13-1 INO80*^{FLAG}	5656	W303 *cdc13-1, INO80*^{5FLAG}*::hphMX6*	3H	This study
W303 *cdc13-1 INO80*$^{AID*-FLAG}$	5657	W303 *cdc13-1, his3-11-15::pRS303-ADH-AFB2(HIS3), INO80*$^{AID*-FLAG}$*::hphNT1*	3H	This study
TET-RAD18 INO80$^{AID*-FLAG}$ *RFA1*^{GFP} *swr1Δ*	5207	*TET-RAD18 INO80*$^{AID*-FLAG}$*, RFA1*^{GFP}*::klTRP1, swr1Δ::natNT2*	4E-F, EV4A-B	This study
TET-RAD18 INO80$^{AID*-FLAG}$ *RFA1*^{GFP} *htz1Δ*	5204	*TET-RAD18 INO80*$^{AID*-FLAG}$*, RFA1*^{GFP}*::klTRP1, htz1Δ::natNT2*	4E-F, EV4A-B	This study
TET-RAD18 INO80$^{AID*-FLAG}$ *RFA1*^{GFP} *swr1Δ htz1Δ*	5124	*TET-RAD18 INO80*$^{AID*-FLAG}$ *RFA1*^{GFP} *swr1Δ, htz1Δ::CaUra3*	4E-F, EV4A-D	This study
TET-RAD18 BrdU-inc (URA) INO80$^{AID*-FLAG}$ *RFA1*^{GFP}	3467	*TET-RAD18 BrdU-inc(URA) INO80*$^{AID*-FLAG}$*, RFA1*^{GFP}*::natNT2*	EV4E-F	This study
TET-RAD18 BrdU-inc (URA) RFA1^{GFP} *MOT1*$^{AID*-myc}$	5210	*TET-RAD18 BrdU-inc(URA), his3-Δ200::pRS303-ADH-AFB2(HIS3), RFA1*^{GFP}*::klTRP1, MOT1*$^{AID*-myc}$*::natNT2*	EV4E-F	This study
TET-RAD18 BrdU-inc (URA) RFA1^{GFP} *MOT1*$^{AID*-myc}$ *INO80*$^{AID*-FLAG}$	5211	*TET-RAD18 BrdU-inc MOT1*$^{AID*-myc}$ *RFA1*^{GFP}*, INO80*$^{AID*-FLAG}$*::hphNT1*	EV4E-F	This study
INO80$^{AID*-FLAG}$ *RFA1*^{GFP}	3525	*RFA1*^{GFP} *AFB2, INO80*$^{AID*-FLAG}$*::hphNT1*	EV4G-H	This study
RPO21$^{AID*-myc}$ *RFA1*^{GFP}	5578	*RFA1*^{GFP} *AFB2, RPO21*$^{AID*-myc}$*::natNT2*	EV4G-H	This study
INO80$^{AID*-FLAG}$ *RPO21*$^{AID*-myc}$ *RFA1*^{GFP}	5579	*INO80*$^{AID*-FLAG}$ *RFA1*^{GFP}*, RPO21*$^{AID*-myc}$*::natNT2*	EV4G-H	This study
TET-RAD18 INO80^{FLAG} *CTF4*^{HA} *RFA1*^{myc}	6051	*TET-RAD18 INO80*^{5FLAG}*::hphMX6 CTF4*^{6HA}*::natNT2 RFA1*^{9myc}*::klTRP*	5A-B, EV5A	This study

Appendix Table S2. Plasmids used in this study

Name	ID	Usage	Reference
pFA6a-HIS3MX6	233	Gene knockout (*HIS3MX6* marker)	(Janke *et al*, 2004)
pYM-natNT2	1633	Gene knockout (*natNT2* marker)	(Janke *et al*, 2004)
pFA6a-CaURA3	4761	Gene knockout (*CaURA3* marker)	This study
pFA6a-kanMX4	452	Gene knockout (*kanMX4* marker)	(Janke *et al*, 2004)
pHyg-AID*-6FLAG	2353	Gene tagging (AID*-6xFLAG, *hphNT1* marker)	(Morawska & Ulrich, 2013)
pNat-AID*-9myc	2189	Gene tagging (AID*-9myc, *natNT2* marker)	(Morawska & Ulrich, 2013)
pYM-yeGFP-Nat	2442	Gene tagging (yeGFP, *natNT2* marker)	This study
pYM6	229	Gene tagging (9myc, *klTRP* marker)	(Janke *et al*, 2004)
pYM17	3281	Gene tagging (6HA, *natNT2* marker)	(Janke *et al*, 2004)
pYM26	2413	Gene tagging (yeGFP, *klTRP* marker)	(Janke *et al*, 2004)
pYM44	2123	Gene tagging (yeGFP, *HIS3MX6* marker)	(Janke *et al*, 2004)
pFA6a-5×FLAG-hphMX	4085	Gene tagging (5xFLAG, *hphMX6* marker)	(Noguchi *et al*, 2008)
pRS303-ADH-AFB2	2397	Expression of AFB2 (*HIS3* marker)	This study
YIp204-ADH-AFB2	2444	Expression of AFB2 (*TRP1* marker)	This study
YIp204-P30-His-POL30	907	Expression of His_6-tagged *POL30* (^{His}PCNA) (*TRP1* marker)	(Davies & Ulrich, 2012)
YIp128-P30-His-POL30	732	Expression of His_6-tagged *POL30* (^{His}PCNA) (*LEU2* marker)	(Davies & Ulrich, 2012)
p306-BrdU-inc	1462	BrdU incorporation cassette (*URA3* marker)	(Viggiani & Aparicio, 2006)
p404-BrdU-inc	1460	BrdU incorporation cassette (*TRP1* marker)	(Viggiani & Aparicio, 2006)
pFBDM_1	N/A	Production of Ino80^{2xFLAG}, Rvb1, Rvb2, Ies6, and Arp5^{His}	(Oberbeckmann *et al*, 2021a)
pFBDM_2	N/A	Production of Actin, Arp4, Arp8, Taf14, Ies2, Ies4, Ies1, Ies3, Ies5, and Nhp10	(Oberbeckmann *et al*, 2021a)
pFBDM_3	N/A	Production of Ino80(Δ1-461)^{2xFLAG}, Rvb1, Rvb2, Ies6, and Arp5^{His}	(Oberbeckmann *et al*, 2021a)
pFBDM_4	N/A	Production of Actin, Arp4, Arp8, Taf14, Ies2, and Ies4	(Oberbeckmann *et al*, 2021a)
pUC19-0N80 overhang	6147	Template for nucleosome sliding assay substrates	This study

Appendix Table S3. Oligonucleotides used in this study

Name	ID	Sequence (5' → 3')
**Gene knockout and tagging**		
ARP8 3' fwd	2303	GGGATGTTCACGGGAGTAGAATCTTACAATATAAATGTATTTTCACGTACCGTACGCTGCAGGTCGAC
ARP8 3' rev	2304	TGCAAAGACCTTTCAGAAAAAAAGATAACAAAAACTTCCATATGCATATCATCGATGAATTCGAGCTCG
ARP8 5' fwd	2305	ATTACTAGTCAATAGTACATAAATACAGGGATACAATCGCACCTAACATGCGTACGCTGCAGGTCGAC
INO80 3' fwd	2298	GCAAAAGCATAAGTCAAGATGGAATTAAGGAAGCGGCAAGTGCATTGGCACGTACGCTGCAGGTCGAC
INO80 3' rev	2299	GATAGACATTAACTCCGCTTAATGTAAATAACACAATATGAATACCTTTTATCGATGAATTCGAGCTCG
INO80 5' fwd	2300	ATATTAGCAAAGCAAGGCTTAAGACATATAGAAGAGCATTTATAGACATGCGTACGCTGCAGGTCGAC
INO80-5FLAG 3' fwd	6104	GCAAAAGCATAAGTCAAGATGGAATTAAGGAAGCGGCAAGTGCATTGGCACGGATCCCCGGGTTAATTAA
ARP5 3' fwd	2540	CAGAGTACATCAAAGAGCATAAGTTAGGGAATACGAAGTATTTTGAAGACCGTACGCTGCAGGTCGAC
ARP5 3' rev	2541	CTATTTATTTCTCTTTTTTTGTTTTTAAGGCGTTTCAGTTTGCTGTCTCCATCGATGAATTCGAGCTCG
NHP10 3' fwd	2535	AAGTAGCTGATTCTAAAGGAGGTGAAGATGGAAGTTTAGTTTCCTCTAACCGTACGCTGCAGGTCGAC
NHP10 3' rev	2536	CGTCTTACGATATCTTCAAAGAAAATAGAAAAAAATGGAATTTTTAATTTATCGATGAATTCGAGCTCG
RFA1 3' fwd	311	GAAGCCGACTATCTTGCCGATGAGTTATCCAAGGCTTTGTTAGCTCGTACGCTGCAGGTCGAC
RFA1 3' rev	312	TCTCATATGTTACATAGATTAAATAGTACTTGATTATTTGATACAATCGATGAATTCGAGCTCG
UBC13 5' fwd	135	CCAATATTAGCAAATAAGGTCAGGTTCATTGTAACATAGTTAGAACGTACGCTGCAGGTCGAC
UBC13 3' rev	120	GATATATATTTATATATTCAGTTGAGAAAACTTATACAGAAATGAATCGATGAATTCGAGCTCG
POL30 5' fwd	481	TCACAGCAACAAGCAGCAAGCACTAAGTACGCAGTCAAAAGAGAGAAAAACGTACGCTGCAGGTCGAC
POL30 3' rev	367	TTTTTTTTTGTTTATTATTTTTAGTATACAACTATATAGATAATTTACATATCGATGAATTCGAGCTCG
POL32 5' fwd	777	ACATTAACTAACAACCAGAAATAGGCTTTAGTTAACTCAATCGGTAATTACGTACGCTGCAGGTCGAC
POL32 3' rev	382	ATACATTACATCACAATTAGTAATGGAAAGTGTTTGGAAAAAAAAGAAGAATCGATGAATTCGAGCTCG
SWR1 5' fwd	5459	TTCTAACTGCTCTTTGCATTTTCCAAGTTATTGCATTACAAGAATATATGCGTACGCTGCAGGTCGAC
SWR1 3' rev	5461	TCCGATTTGGACAACTAAGGCAGCGGTGAAGAGTAGAACCTGGTCCTTCAATCGATGAATTCGAGCTCG
HTZ1 5' fwd	2702	CGTTAAATTCAATTTCGCACTATAGCCGCACGTAAAAATAACTTAACATACGTACGCTGCAGGTCGAC
HTZ1 3' rev	2701	ATACAGGAGCAGGGAGAATTACGGGAAATGGGAAAGAAAAACTATTCTTCATCGATGAATTCGAGCTCG
MOT1 3' fwd	5675	CTCAATACGAGGAGGAGTATAATTTAGACACCTTCATCAAAACTTTACGACGTACGCTGCAGGTCGAC
MOT1 3' rev	5676	AAACAAAAATGACCTTGTATACGCGTCATTCCAATGCAAGAATTTGTTTAATCGATGAATTCGAGCTCG
RPO21 3' fwd	6600	ATTCTCCAAAGCAAGACGAACAAAAGCATAATGAAAATGAAAATTCCAGACGTACGCTGCAGGTCGAC
RPA21 3' rev	6599	CTATATATAATGTAATAACGTCAAATACGTAAGGATGATATACTATATCAATCGATGAATTCGAGCTCG
**Preparation of nucleosome sliding assay substrates**		
0N80 #1	7145	/5FluorT/CGGTACCCGGGGATCCT
0N80 #2	7146	/5Phos/TCGGTACCCGGGGATCCT
0N80 #3	7150	/5Phos/CAAAGAAACCGGAATCAAAAAAGAA
0N80 #4	7149	CAAAGAAACCGGAATCAAAAAAGAA
0N80 #5	7152	/5Phos/GAACCGGTCTGGAGAATCC
0N80 #6	7151	GAACCGGTCTGGAGAATCC
0N80 fw	N/A	/56-FAM/CTGGAGAATCCCGGTGCCGAGG
0N80 rev	N/A	CGGTACCCGGGGATCCTCTAG

Appendix References

Daigaku Y, Davies AA & Ulrich HD (2010) Ubiquitin-dependent DNA damage bypass is separable from genome replication. *Nature* 465: 951–955

Davies AA & Ulrich HD (2012) Detection of PCNA modifications in *Saccharomyces cerevisiae*. *Methods Mol Biol* 920: 543–567

Finley D, Ozkaynak E & Varshavsky A (1987) The yeast polyubiquitin gene is essential for resistance to high temperatures, starvation, and other stresses. *Cell* 48: 1035–1046

García-Rodríguez N, Wong RP & Ulrich HD (2018b) The helicase Pif1 functions in the template switching pathway of DNA damage bypass. *Nucleic Acids Res* 46: 8347–8356

Janke C, Magiera MM, Rathfelder N, Taxis C, Reber S, Maekawa H, Moreno-Borchart A, Doenges G, Schwob E, Schiebel E, *et al* (2004) A versatile toolbox for PCR-based tagging of yeast genes: new fluorescent proteins, more markers and promoter substitution cassettes. *Yeast* 21: 947–962

Morawska M & Ulrich HD (2013) An expanded tool kit for the auxin-inducible degron system in budding yeast. *Yeast* 30: 341–351

Noguchi C, Garabedian MV, Malik M & Noguchi E (2008) A vector system for genomic FLAG epitope-tagging in *Schizosaccharomyces pombe*. *Biotechnol J* 3: 1280–1285

Oberbeckmann E, Krietenstein N, Niebauer V, Wang Y, Schall K, Moldt M, Straub T, Rohs R, Hopfner K-P, Korber P, *et al* (2021a) Genome information processing by the INO80 chromatin remodeler positions nucleosomes. *Nat Commun* 12: 3231

Saugar I, Parker JL, Zhao S & Ulrich HD (2012) The genome maintenance factor Mgs1 is targeted to sites of replication stress by ubiquitylated PCNA. *Nucleic Acids Res* 40: 245–257

Shimada K, Oma Y, Schleker T, Kugou K, Ohta K, Harata M & Gasser SM (2008) Ino80 chromatin remodeling complex promotes recovery of stalled replication forks. *Curr Biol* 18: 566–575

Ulrich HD & Jentsch S (2000) Two RING finger proteins mediate cooperation between ubiquitin-conjugating enzymes in DNA repair. *EMBO J* 19: 3388–3397

Viggiani CJ & Aparicio OM (2006) New vectors for simplified construction of BrdU-Incorporating strains of *Saccharomyces cerevisiae*. *Yeast* 23: 1045–1051
