# Supplementary figures and images for: The INO80 chromatin remodeller facilitates DNA damage bypass via postreplicative gap repair
Source: EMBO J. 2025 Oct 13;44(22):6626–48. doi: 10.1038/s44318-025-00580-4 (PMC12624141; doi:10.1038/s44318-025-00580-4)

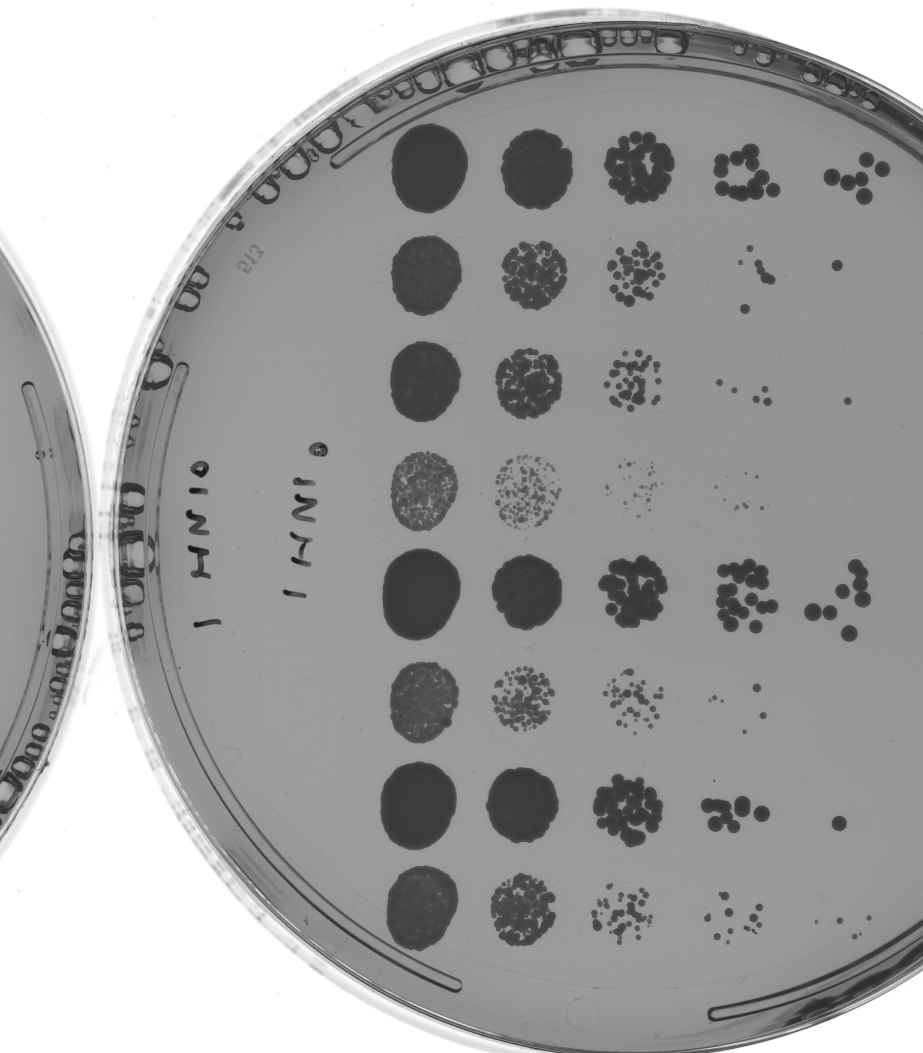

Supplement: Supplementary file 3 — Source data Fig. 1 [file 44318_2025_580_MOESM3_ESM.zip › Figure 1/A/HU 10mM.tif]

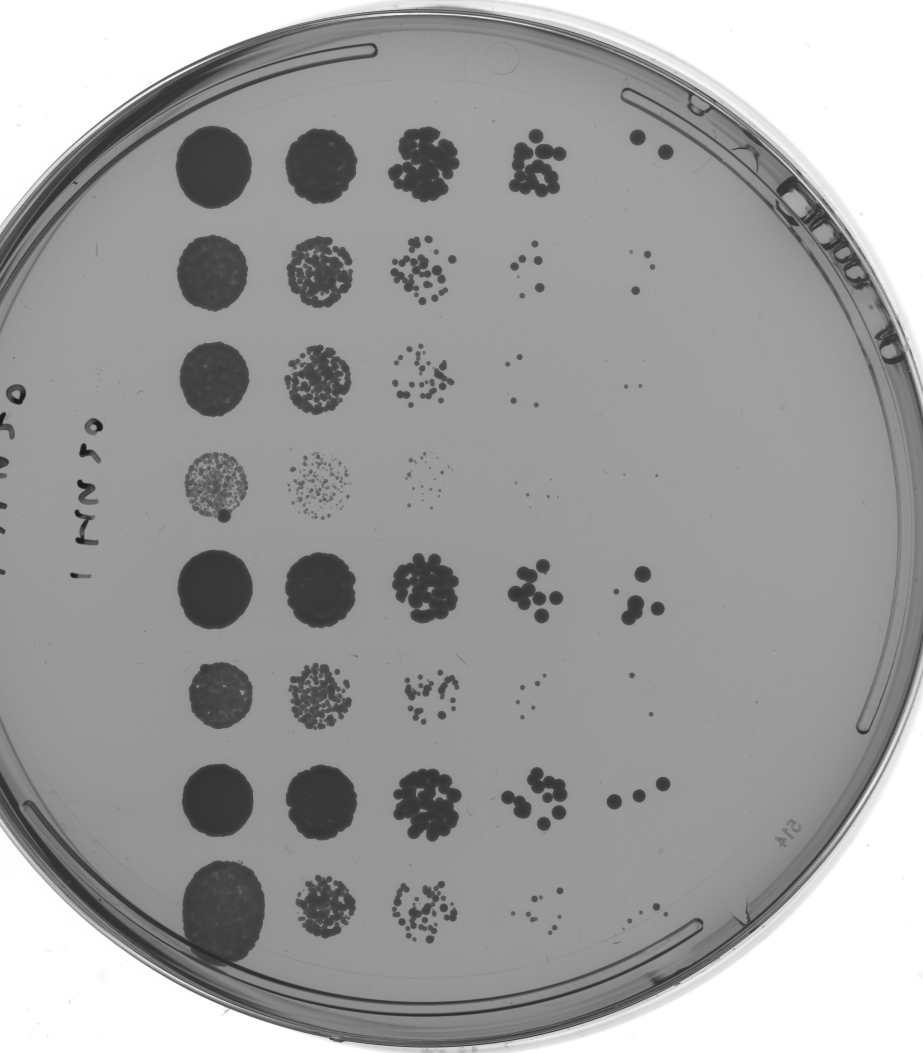

Supplement: Supplementary file 3 — Source data Fig. 1 [file 44318_2025_580_MOESM3_ESM.zip › Figure 1/A/HU 20mM.tif]

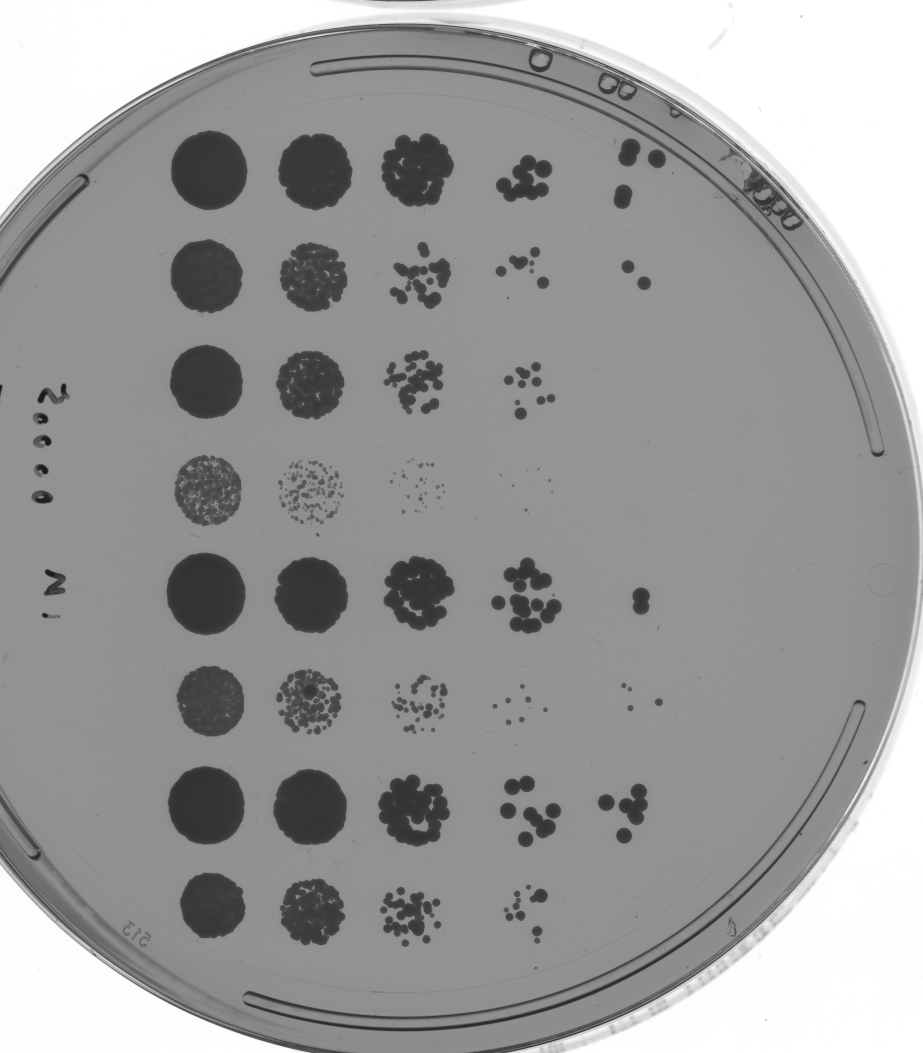

Supplement: Supplementary file 3 — Source data Fig. 1 [file 44318_2025_580_MOESM3_ESM.zip › Figure 1/A/MMS 00005.tif]

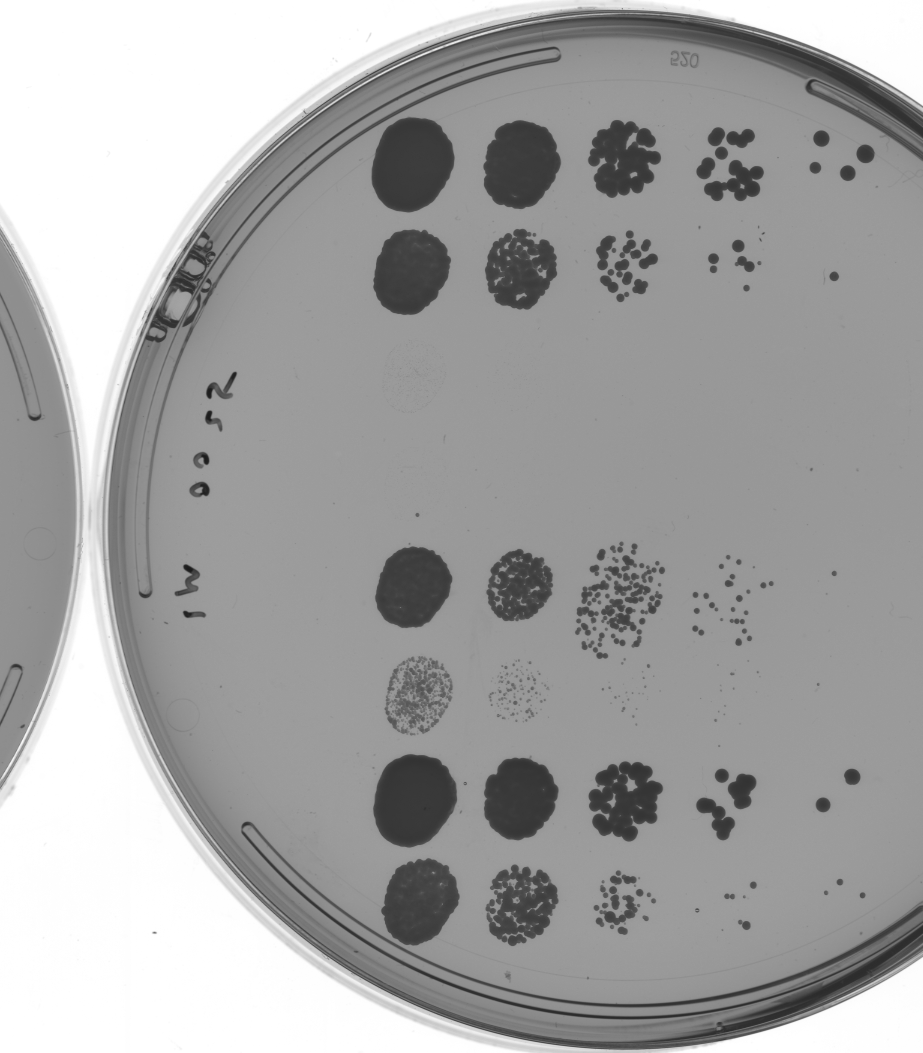

Supplement: Supplementary file 3 — Source data Fig. 1 [file 44318_2025_580_MOESM3_ESM.zip › Figure 1/A/MMS 0025.tif]

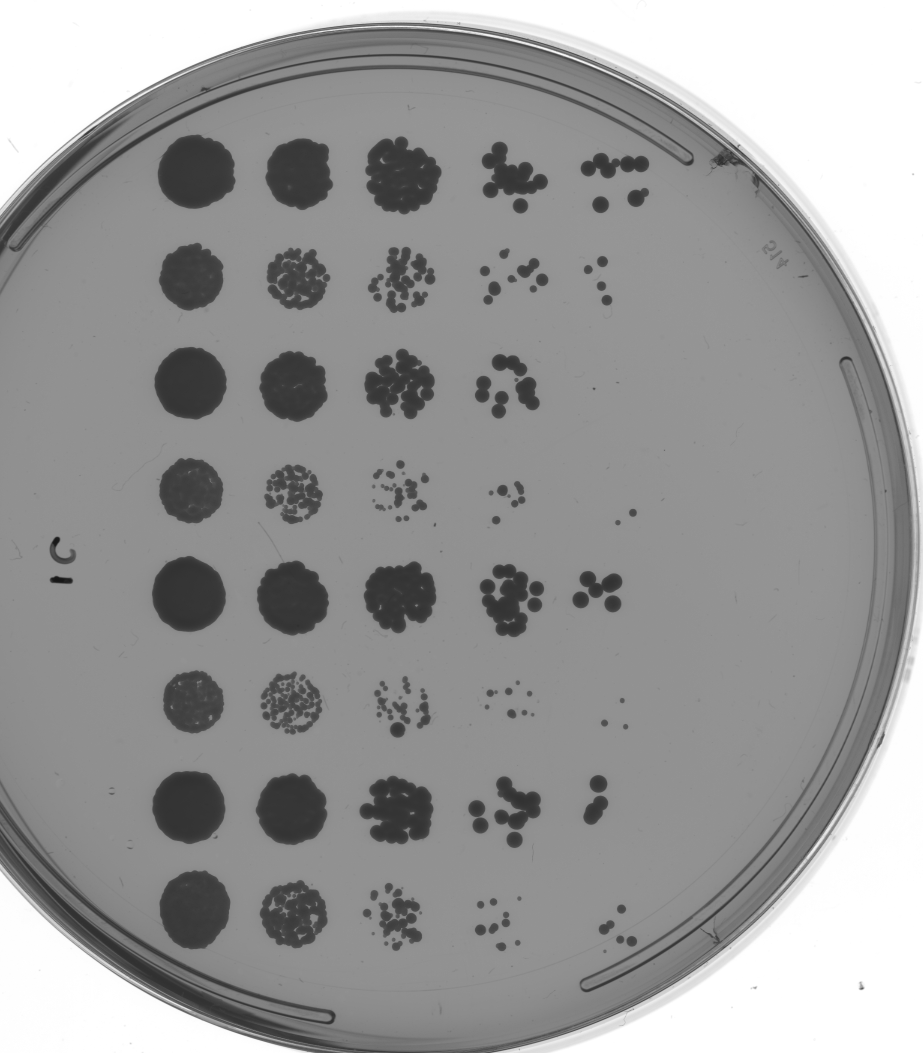

Supplement: Supplementary file 3 — Source data Fig. 1 [file 44318_2025_580_MOESM3_ESM.zip › Figure 1/A/untreated.tif]

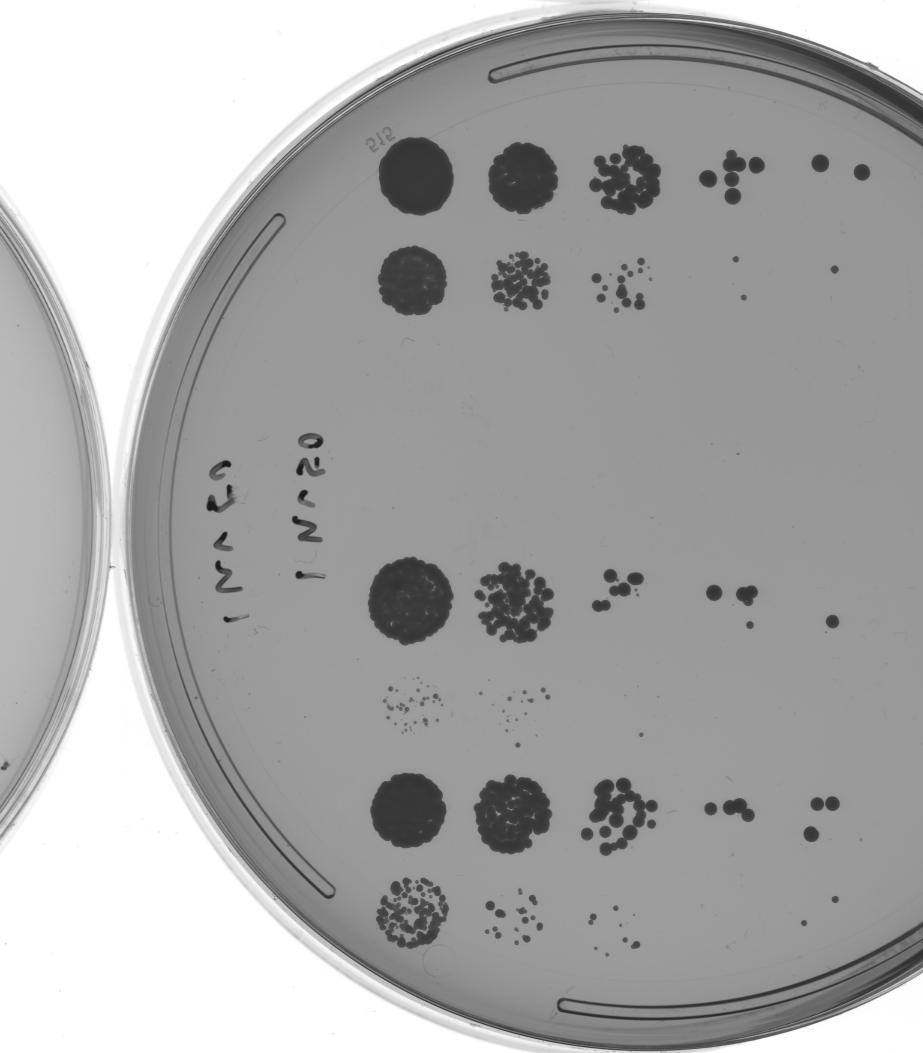

Supplement: Supplementary file 3 — Source data Fig. 1 [file 44318_2025_580_MOESM3_ESM.zip › Figure 1/A/UV 50J.tif]

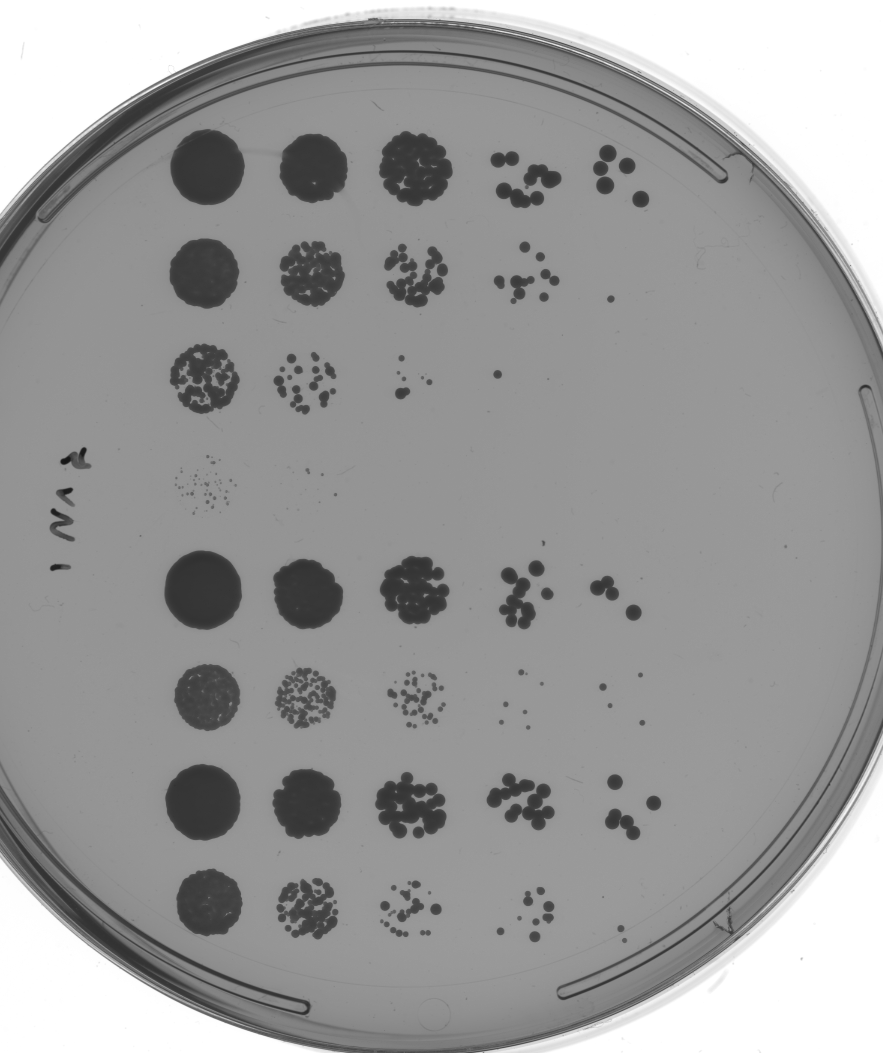

Supplement: Supplementary file 3 — Source data Fig. 1 [file 44318_2025_580_MOESM3_ESM.zip › Figure 1/A/UV 5J.tif]

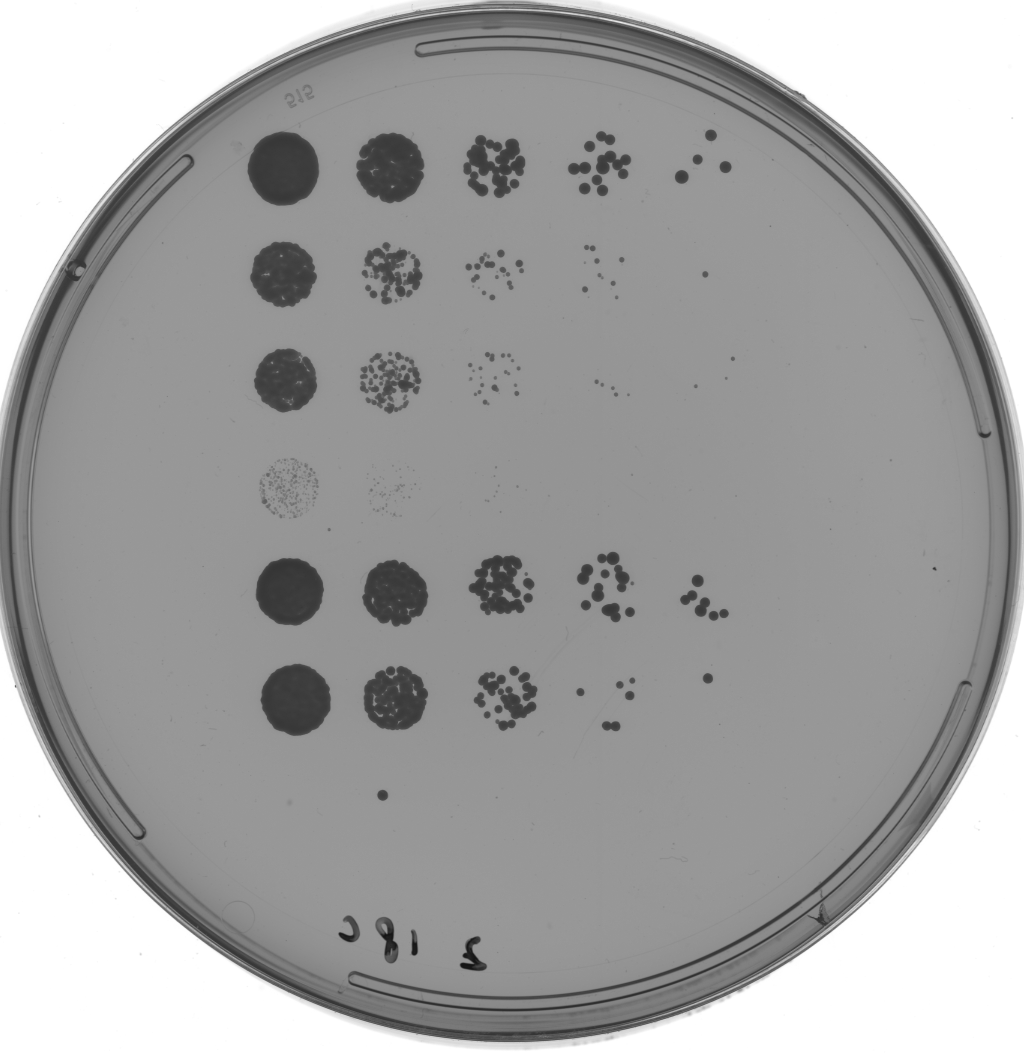

Supplement: Supplementary file 3 — Source data Fig. 1 [file 44318_2025_580_MOESM3_ESM.zip › Figure 1/B/18C.tif]

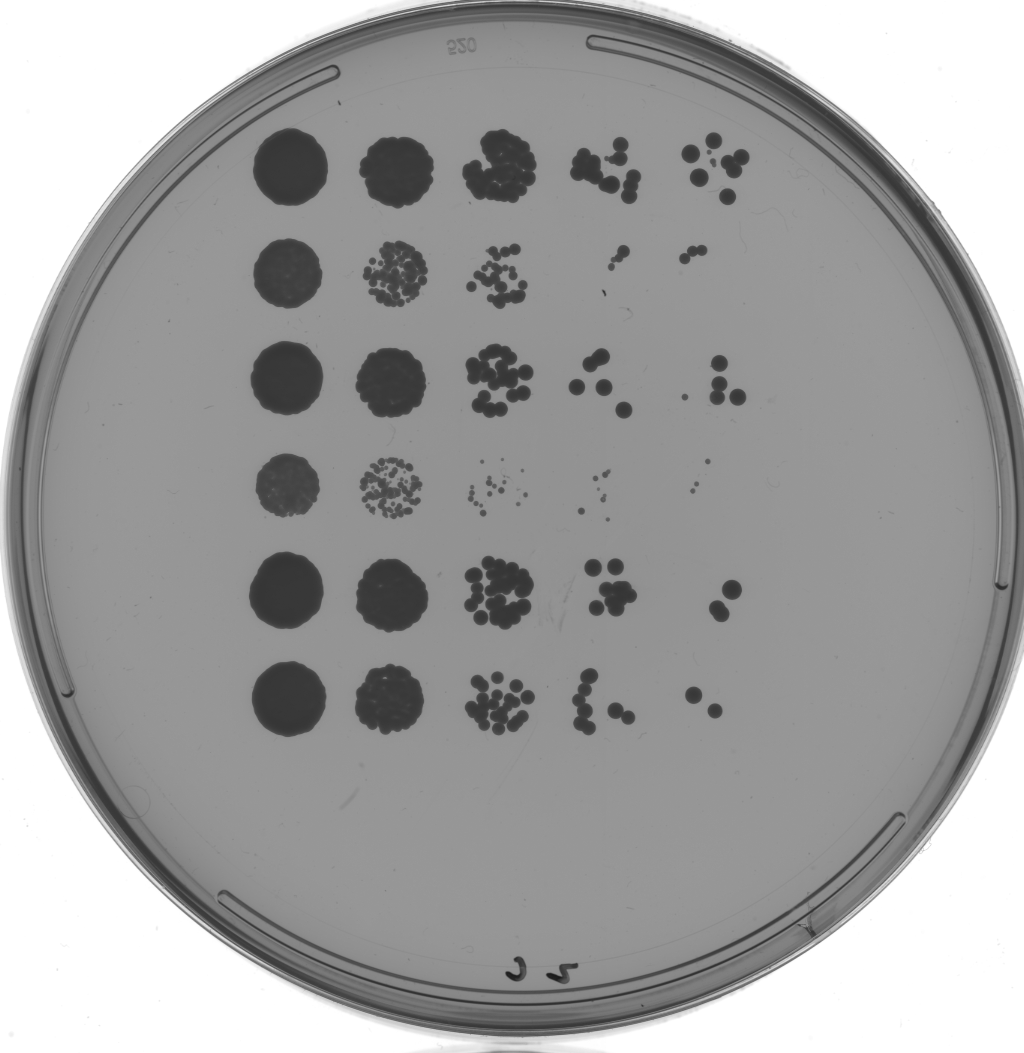

Supplement: Supplementary file 3 — Source data Fig. 1 [file 44318_2025_580_MOESM3_ESM.zip › Figure 1/B/30C.tif]

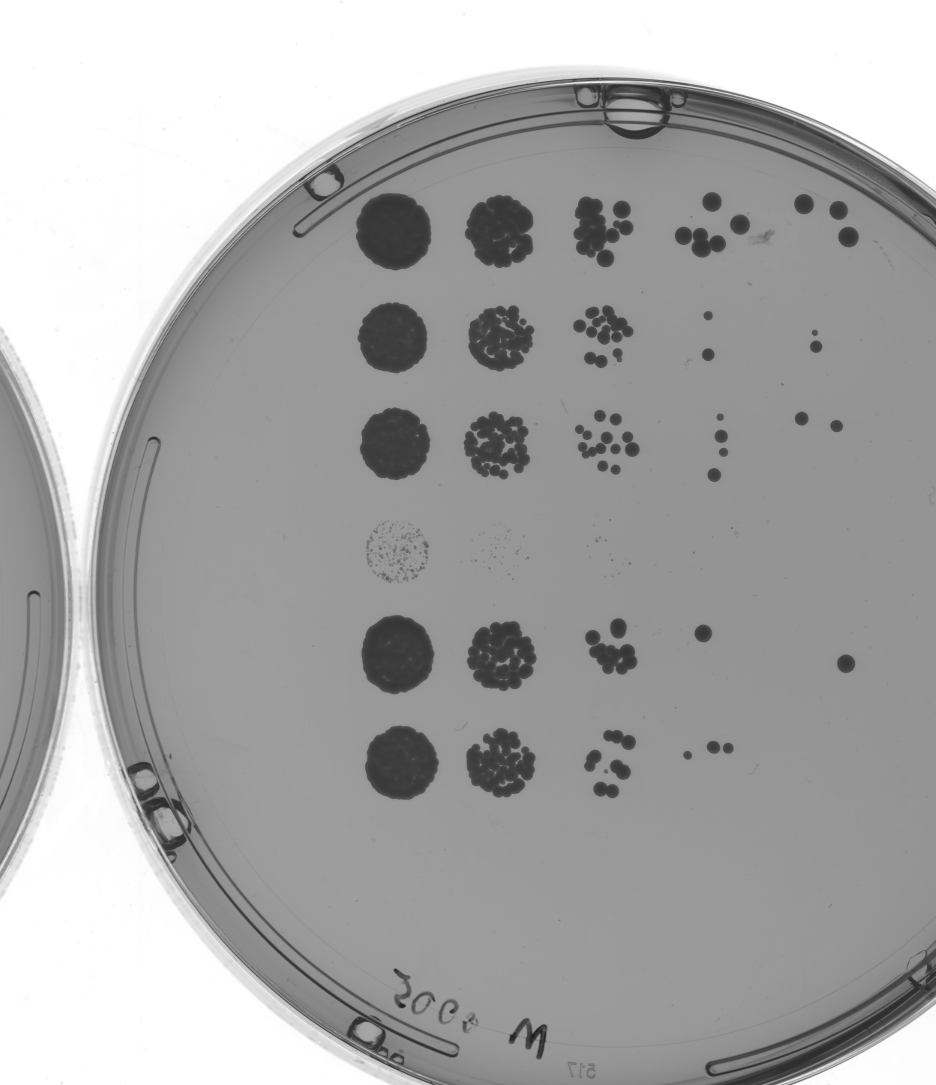

Supplement: Supplementary file 3 — Source data Fig. 1 [file 44318_2025_580_MOESM3_ESM.zip › Figure 1/C/MMS 0005.tif]

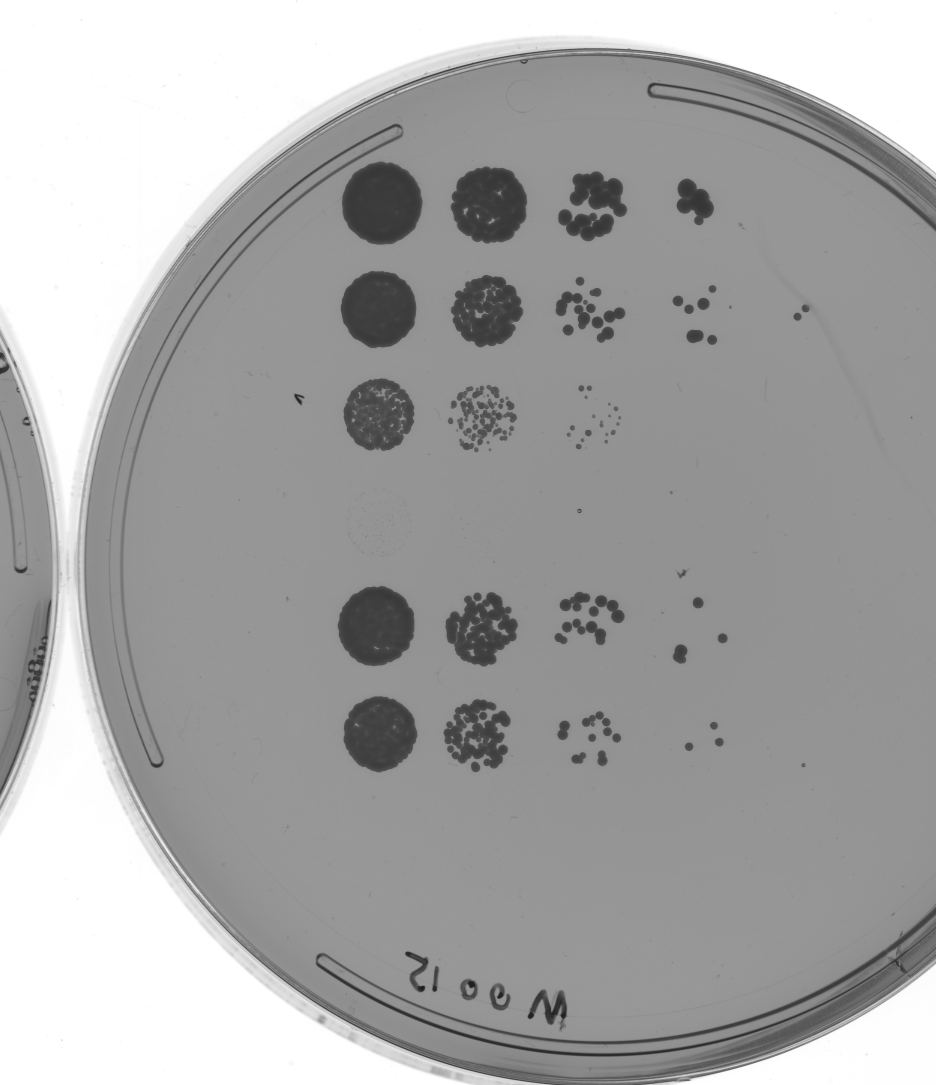

Supplement: Supplementary file 3 — Source data Fig. 1 [file 44318_2025_580_MOESM3_ESM.zip › Figure 1/C/MMS 0015.tif]

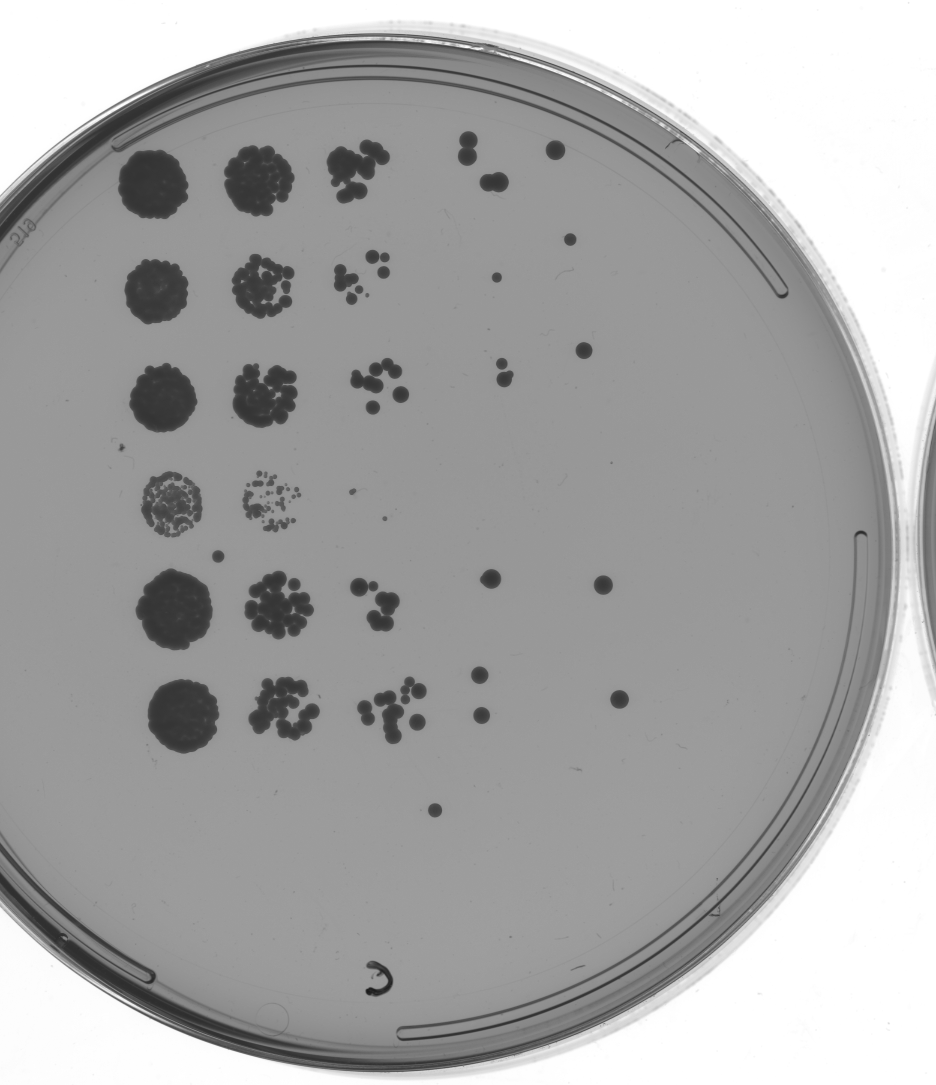

Supplement: Supplementary file 3 — Source data Fig. 1 [file 44318_2025_580_MOESM3_ESM.zip › Figure 1/C/untreated.tif]

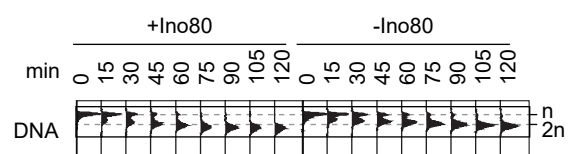

Supplement: Supplementary file 4 — Source data Fig. 2 [file 44318_2025_580_MOESM4_ESM.zip › Figure 2/B/FACS/Figure 2B FACS.pdf]

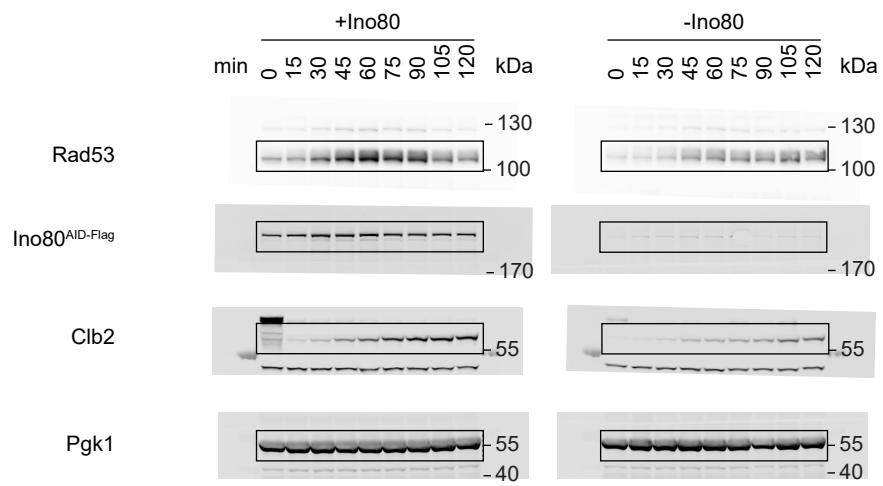

Supplement: Supplementary file 4 — Source data Fig. 2 [file 44318_2025_580_MOESM4_ESM.zip › Figure 2/B/Western/Figure 2B Western.pdf]

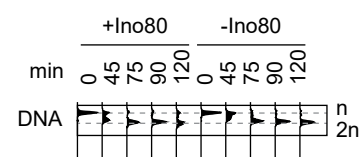

Supplement: Supplementary file 4 — Source data Fig. 2 [file 44318_2025_580_MOESM4_ESM.zip › Figure 2/C/FACS/FIgure 2C FACS.pdf]

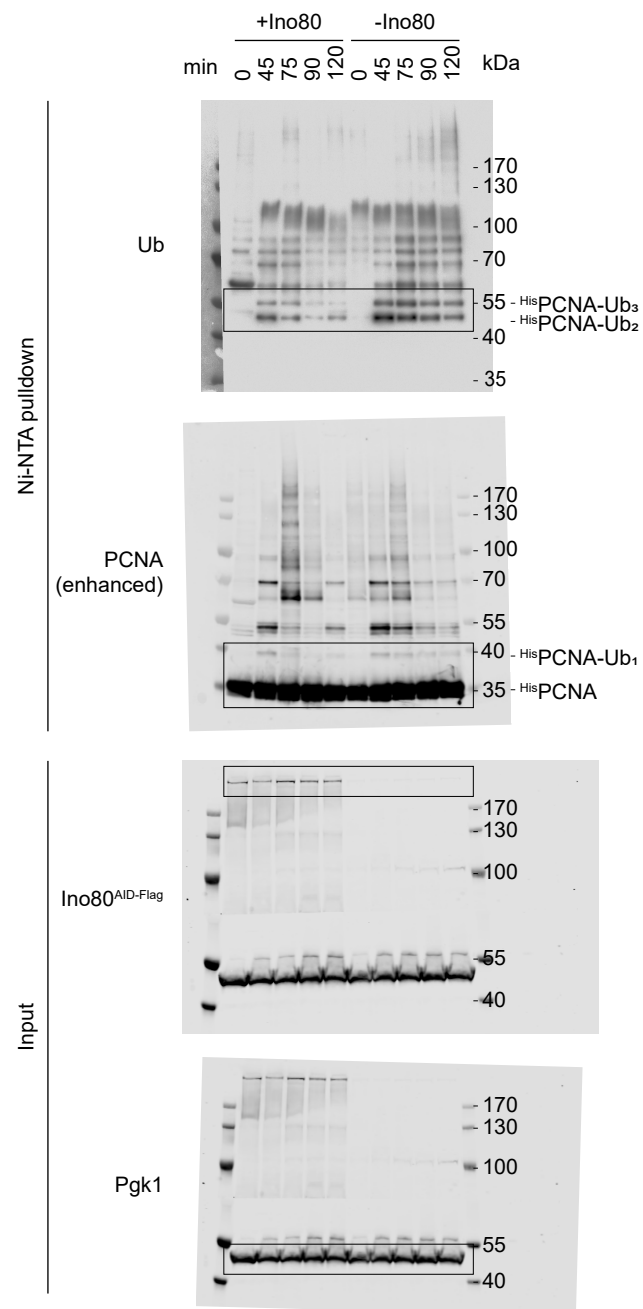

Supplement: Supplementary file 4 — Source data Fig. 2 [file 44318_2025_580_MOESM4_ESM.zip › Figure 2/C/Western/Figure 2C Western.pdf]

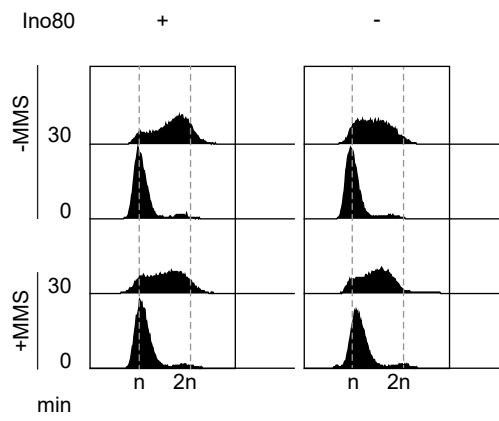

Supplement: Supplementary file 5 — Source data Fig. 3 [file 44318_2025_580_MOESM5_ESM.zip › Figure 3/B/FACS/Figure 3B FACS.pdf]

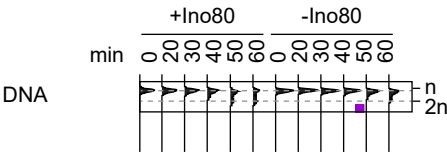

Supplement: Supplementary file 5 — Source data Fig. 3 [file 44318_2025_580_MOESM5_ESM.zip › Figure 3/F/FACS/Figure 3F FACS.pdf]

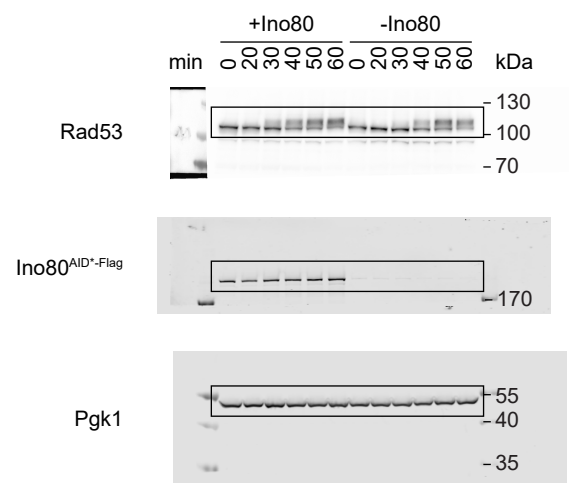

Supplement: Supplementary file 5 — Source data Fig. 3 [file 44318_2025_580_MOESM5_ESM.zip › Figure 3/F/Western/Figure 3F Western.pdf]

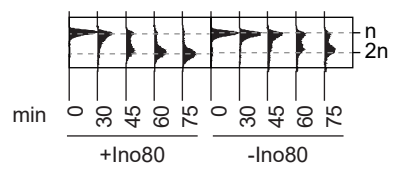

Supplement: Supplementary file 5 — Source data Fig. 3 [file 44318_2025_580_MOESM5_ESM.zip › Figure 3/G/FACS/Figure 3G FACS.pdf]

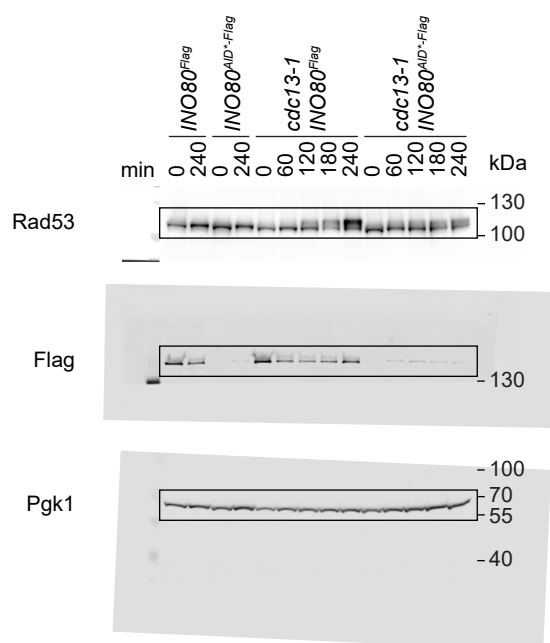

Supplement: Supplementary file 5 — Source data Fig. 3 [file 44318_2025_580_MOESM5_ESM.zip › Figure 3/H/Figure 3H Western.pdf]

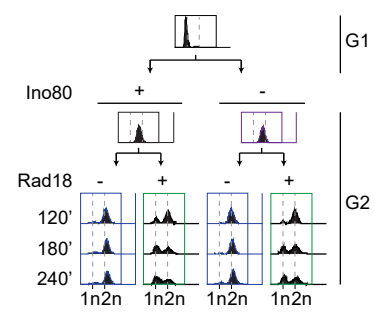

Supplement: Supplementary file 5 — Source data Fig. 3 [file 44318_2025_580_MOESM5_ESM.zip › Figure 3/K/FACS/Figure 3K_FACS.pdf]

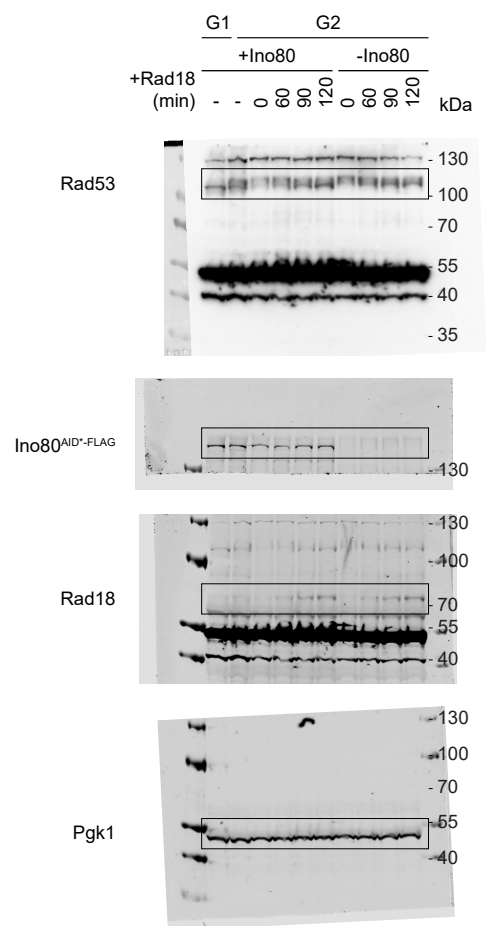

Supplement: Supplementary file 5 — Source data Fig. 3 [file 44318_2025_580_MOESM5_ESM.zip › Figure 3/L/Figure 3L_Western.pdf]

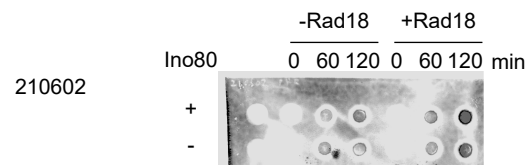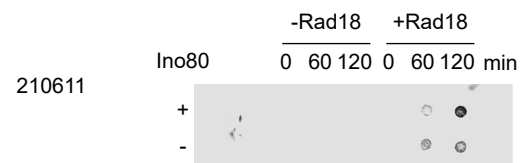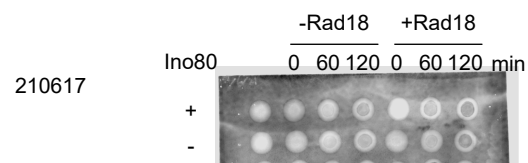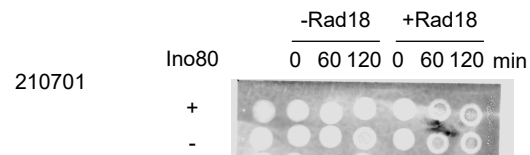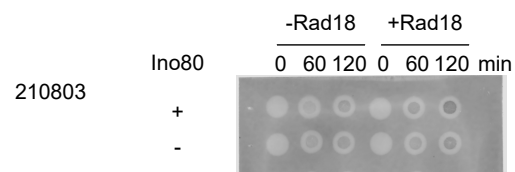

Supplement: Supplementary file 5 — Source data Fig. 3 [file 44318_2025_580_MOESM5_ESM.zip › Figure 3/N/dot blots/Figure 3N_dot blots.pdf]

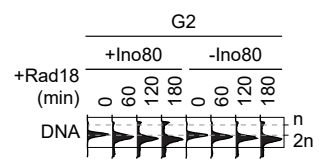

Supplement: Supplementary file 5 — Source data Fig. 3 [file 44318_2025_580_MOESM5_ESM.zip › Figure 3/O/FACS/Figure 3O_FACS.pdf]

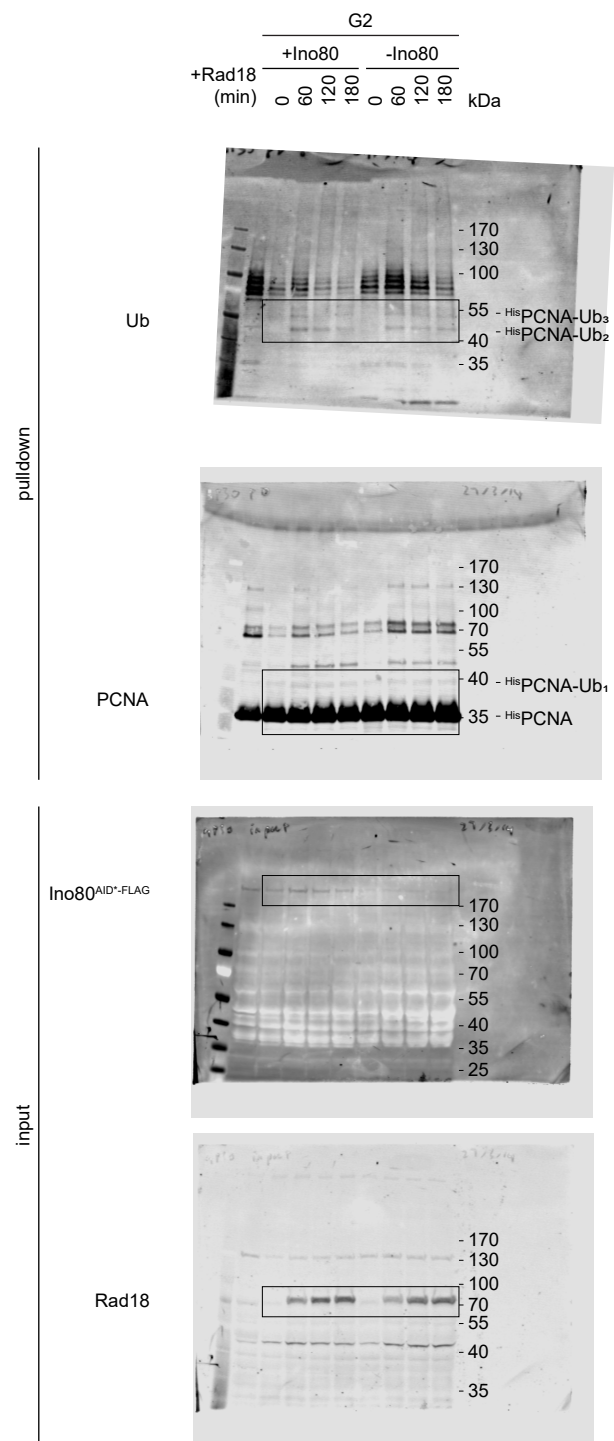

Supplement: Supplementary file 5 — Source data Fig. 3 [file 44318_2025_580_MOESM5_ESM.zip › Figure 3/O/Western/Figure 3O_Western.pdf]

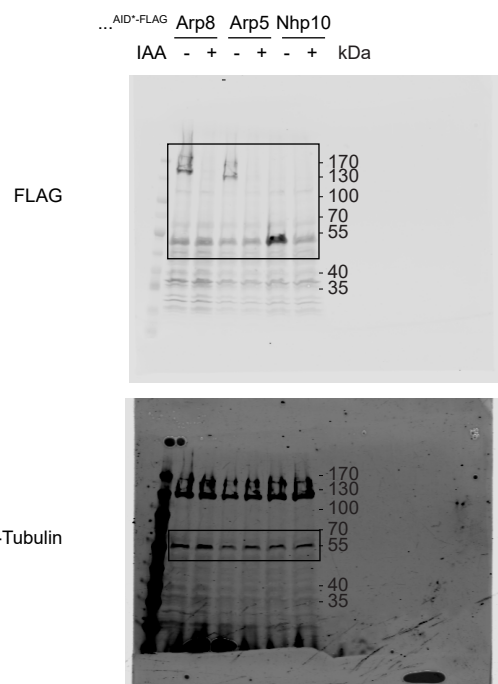

Supplement: Supplementary file 6 — Source data Fig. 4 [file 44318_2025_580_MOESM6_ESM.zip › Figure 4/B/Figure4B_Western.pdf]

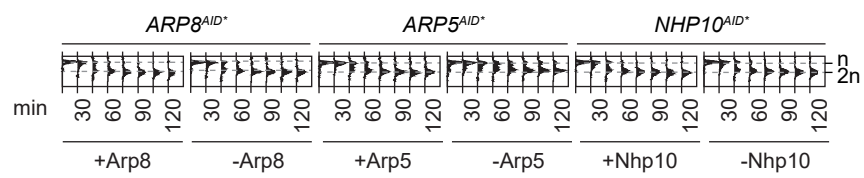

Supplement: Supplementary file 6 — Source data Fig. 4 [file 44318_2025_580_MOESM6_ESM.zip › Figure 4/C/FACS/Figure4C_FACS.pdf]

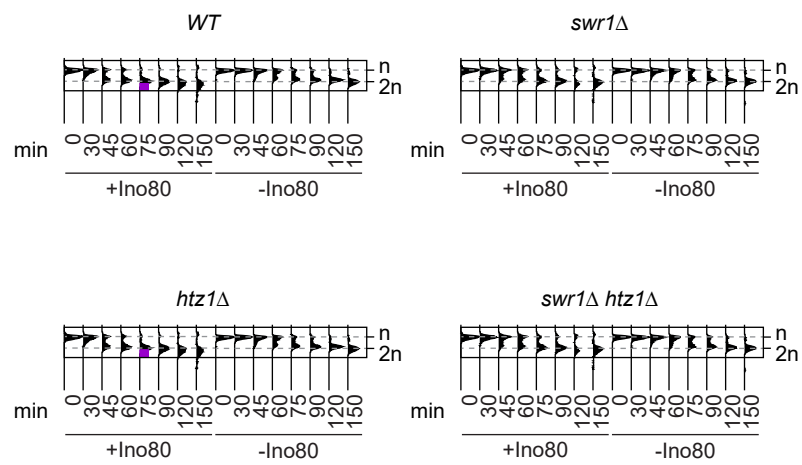

Supplement: Supplementary file 6 — Source data Fig. 4 [file 44318_2025_580_MOESM6_ESM.zip › Figure 4/E/FACS/Figure4E_FACS.pdf]

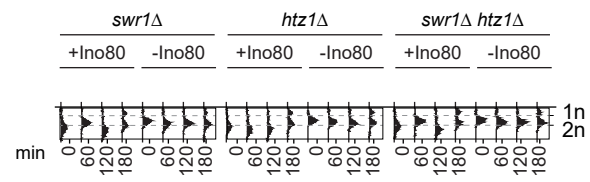

Supplement: Supplementary file 6 — Source data Fig. 4 [file 44318_2025_580_MOESM6_ESM.zip › Figure 4/F/FACS/Figure4F_FACS.pdf]

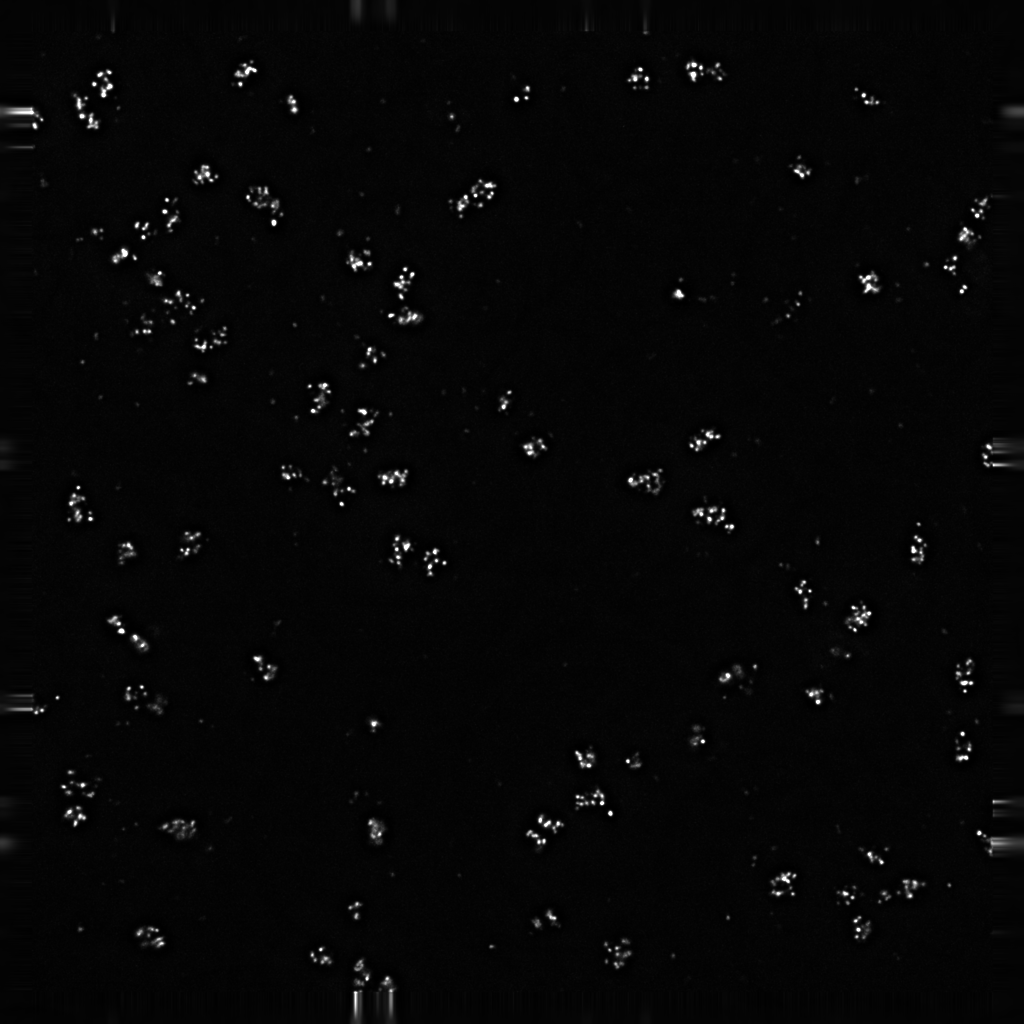

Supplement: Supplementary file 7 — Source data Fig. 5 [file 44318_2025_580_MOESM7_ESM.zip › Figure 5/A/+Rad18_0.tif]

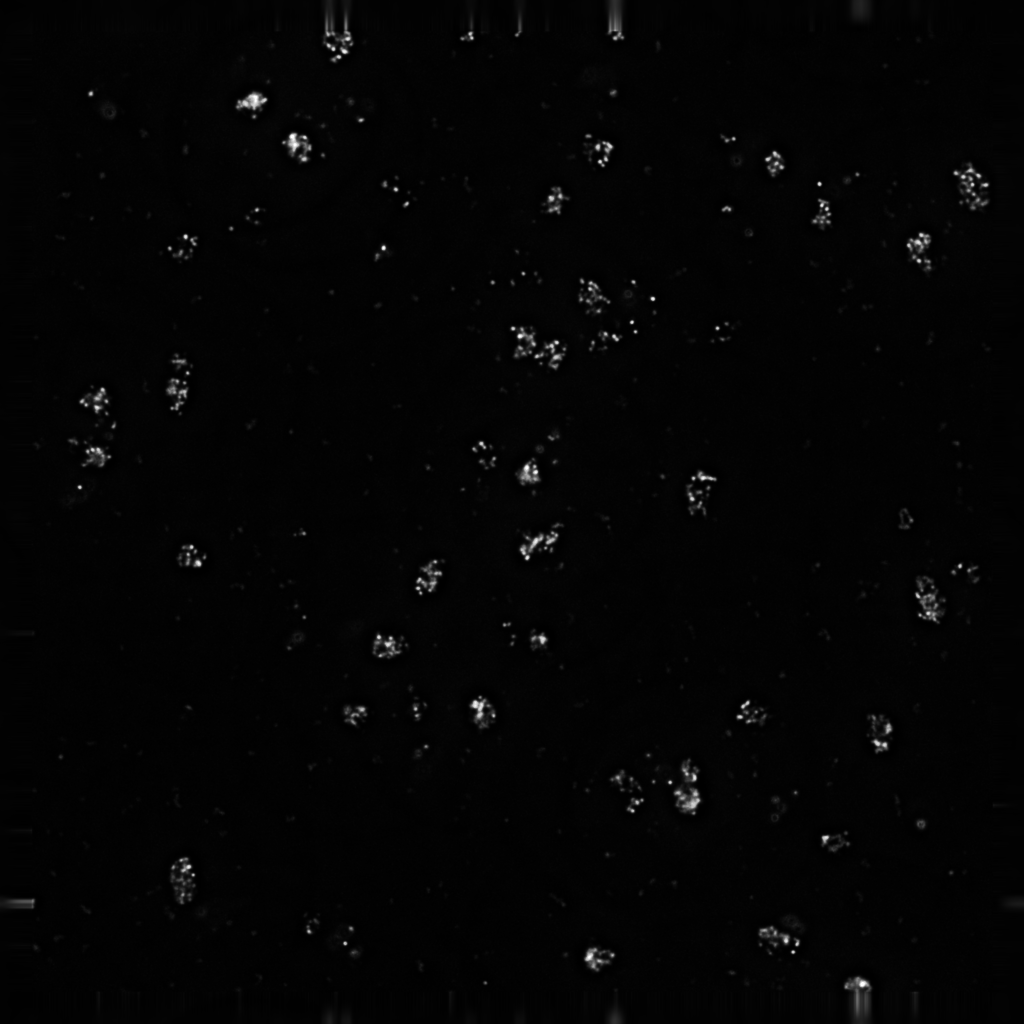

Supplement: Supplementary file 7 — Source data Fig. 5 [file 44318_2025_580_MOESM7_ESM.zip › Figure 5/A/+Rad18_120.tif]

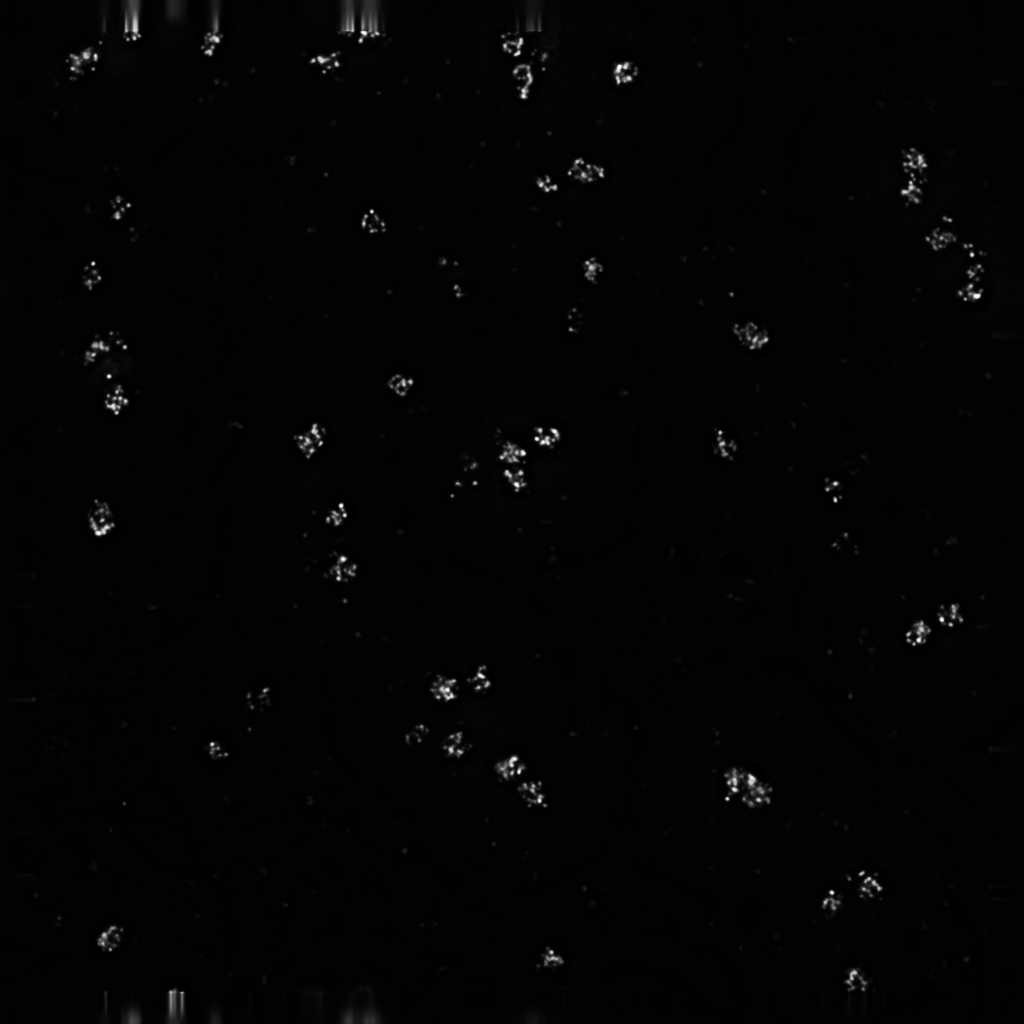

Supplement: Supplementary file 7 — Source data Fig. 5 [file 44318_2025_580_MOESM7_ESM.zip › Figure 5/A/+Rad18_40.tif]

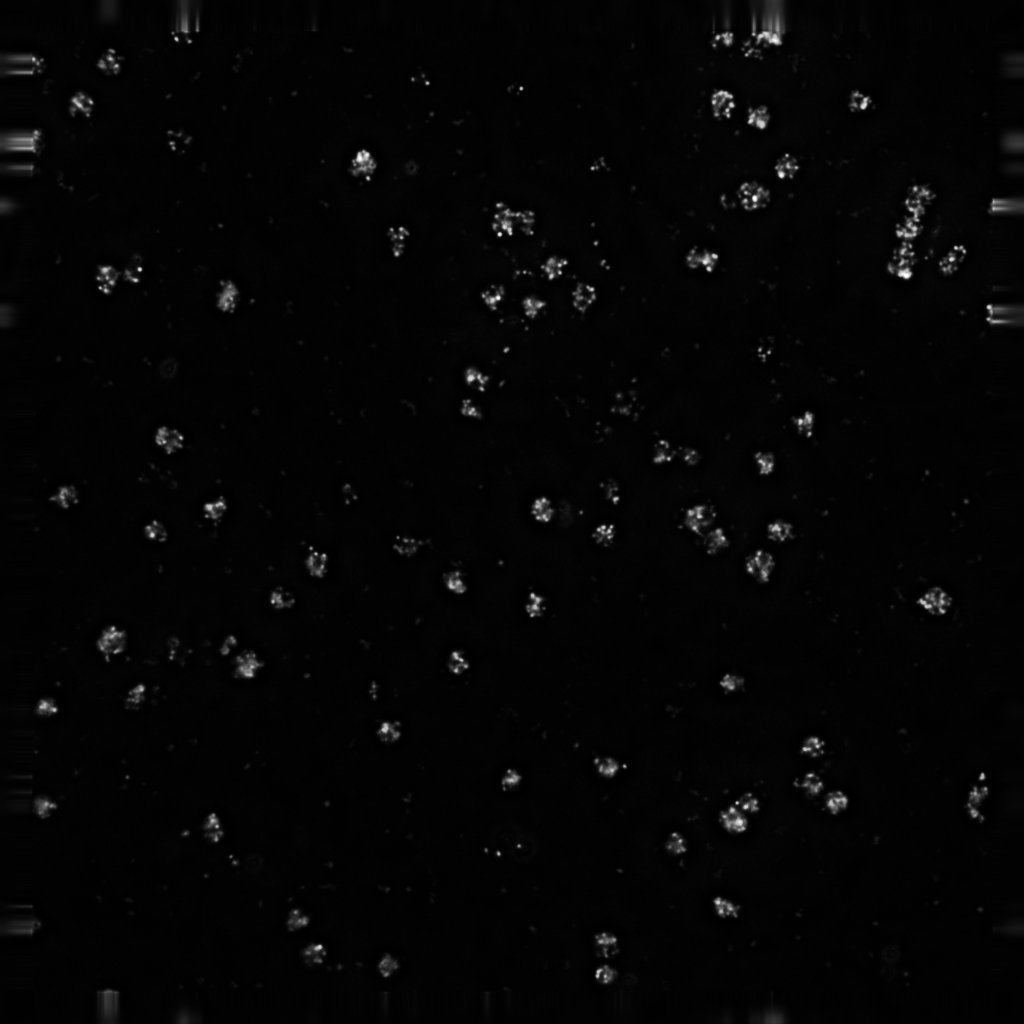

Supplement: Supplementary file 7 — Source data Fig. 5 [file 44318_2025_580_MOESM7_ESM.zip › Figure 5/A/+Rad18_80.tif]

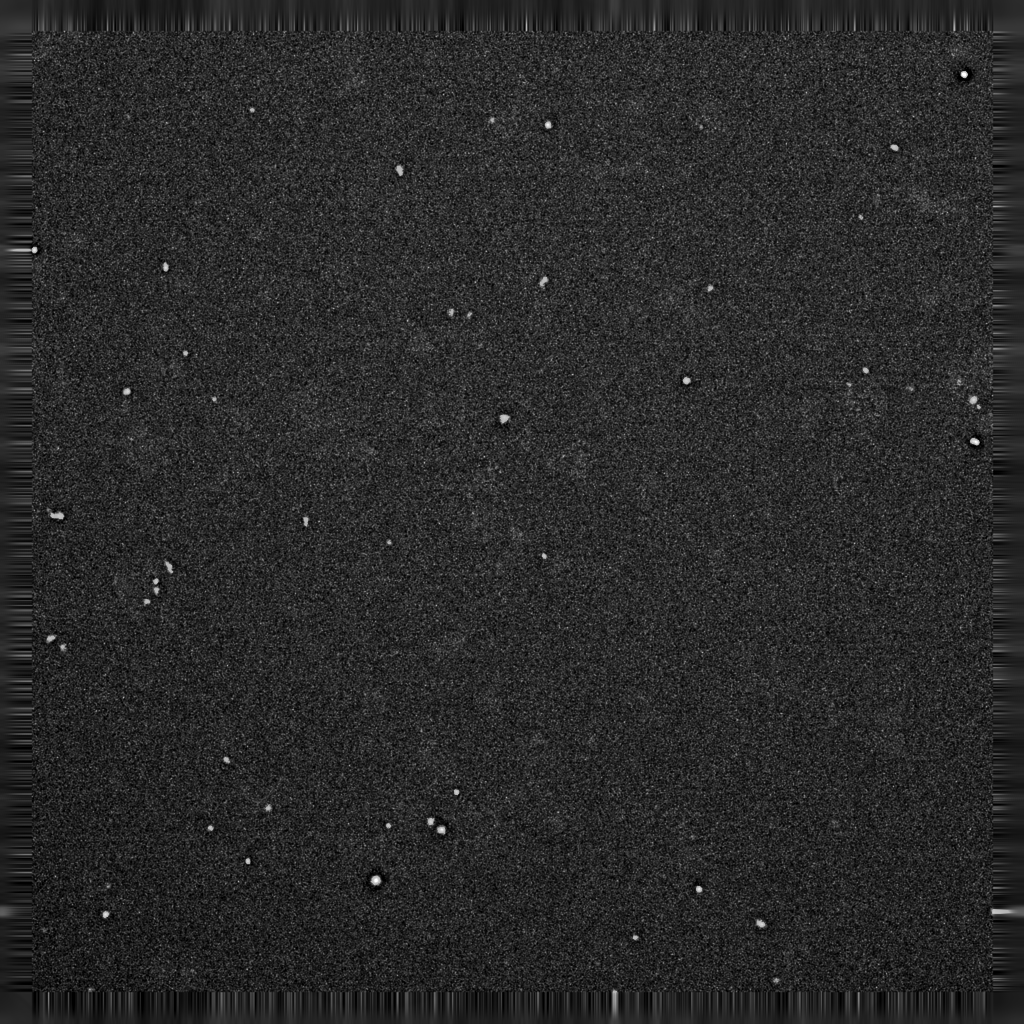

Supplement: Supplementary file 7 — Source data Fig. 5 [file 44318_2025_580_MOESM7_ESM.zip › Figure 5/A/no tag.tif]

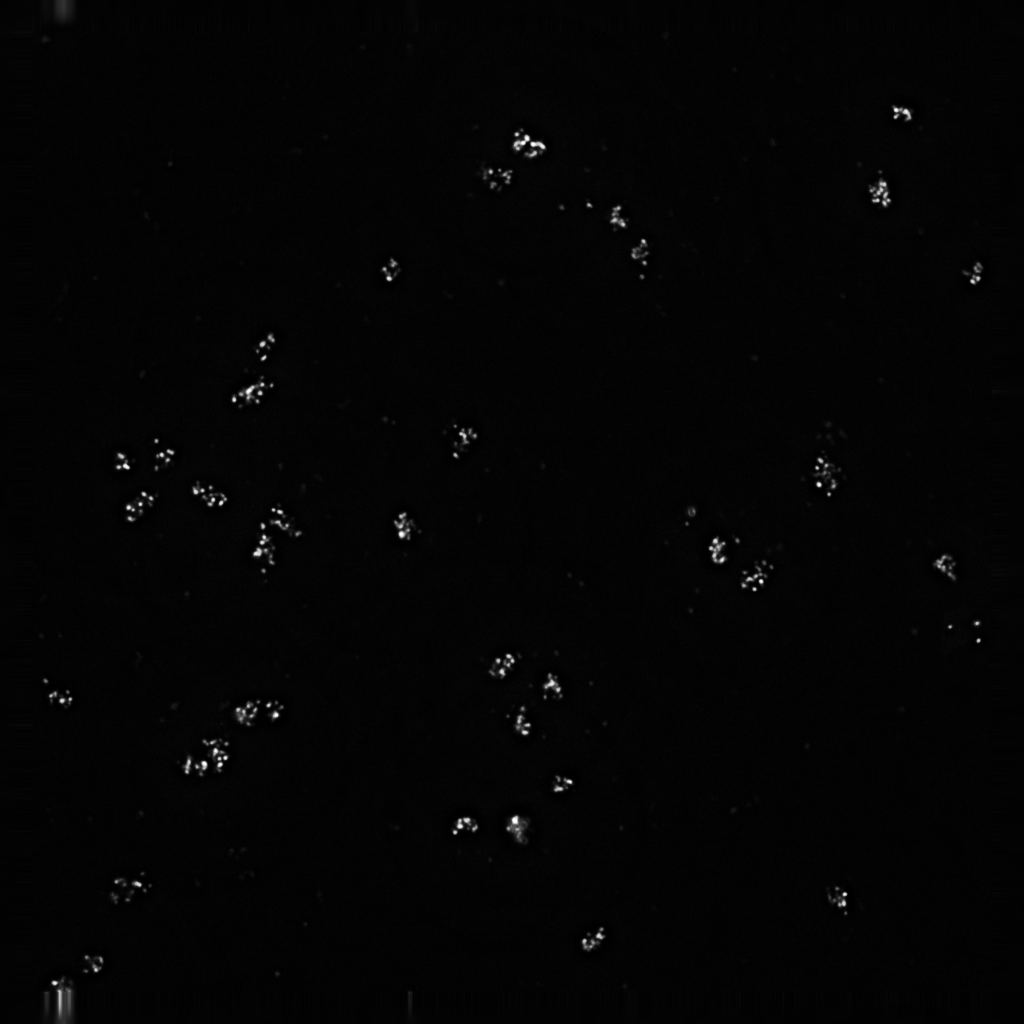

Supplement: Supplementary file 7 — Source data Fig. 5 [file 44318_2025_580_MOESM7_ESM.zip › Figure 5/A/-Rad18_0.tif]

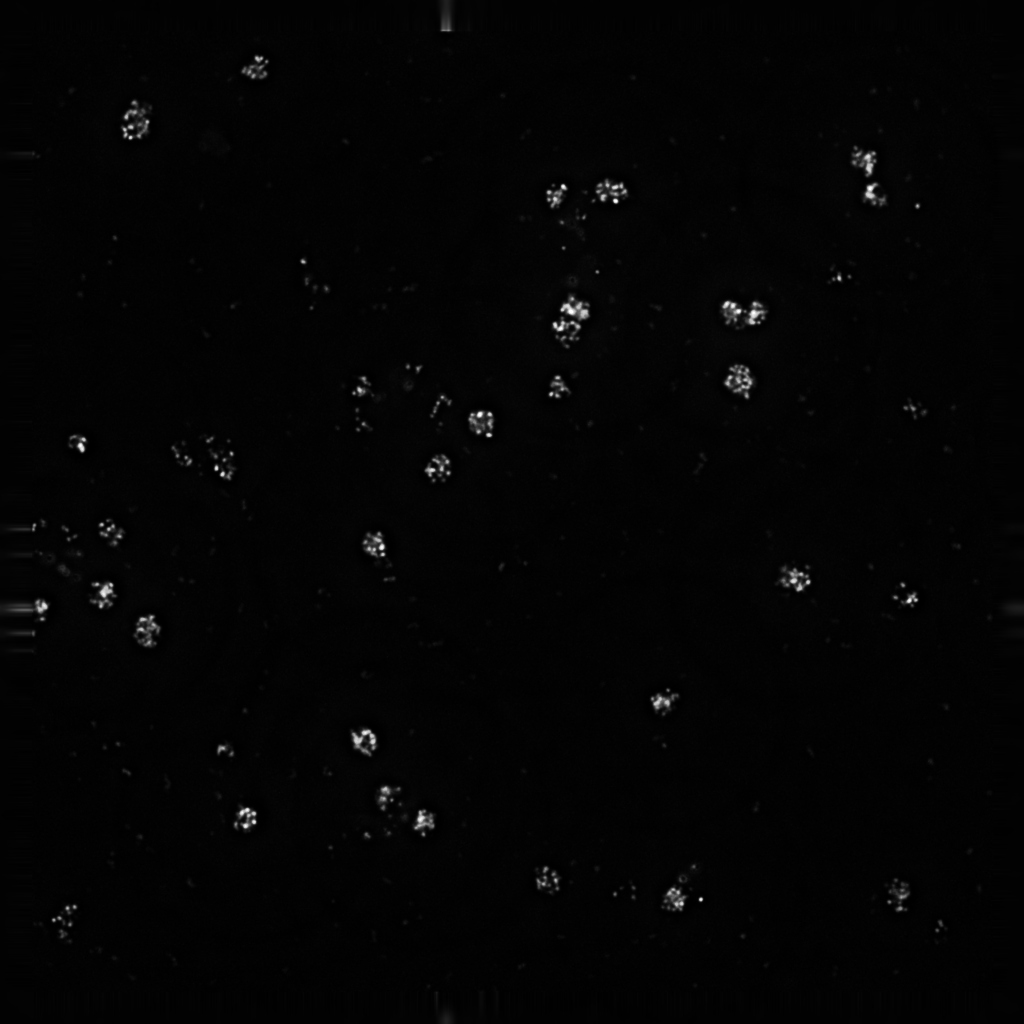

Supplement: Supplementary file 7 — Source data Fig. 5 [file 44318_2025_580_MOESM7_ESM.zip › Figure 5/A/-Rad18_120.tif]

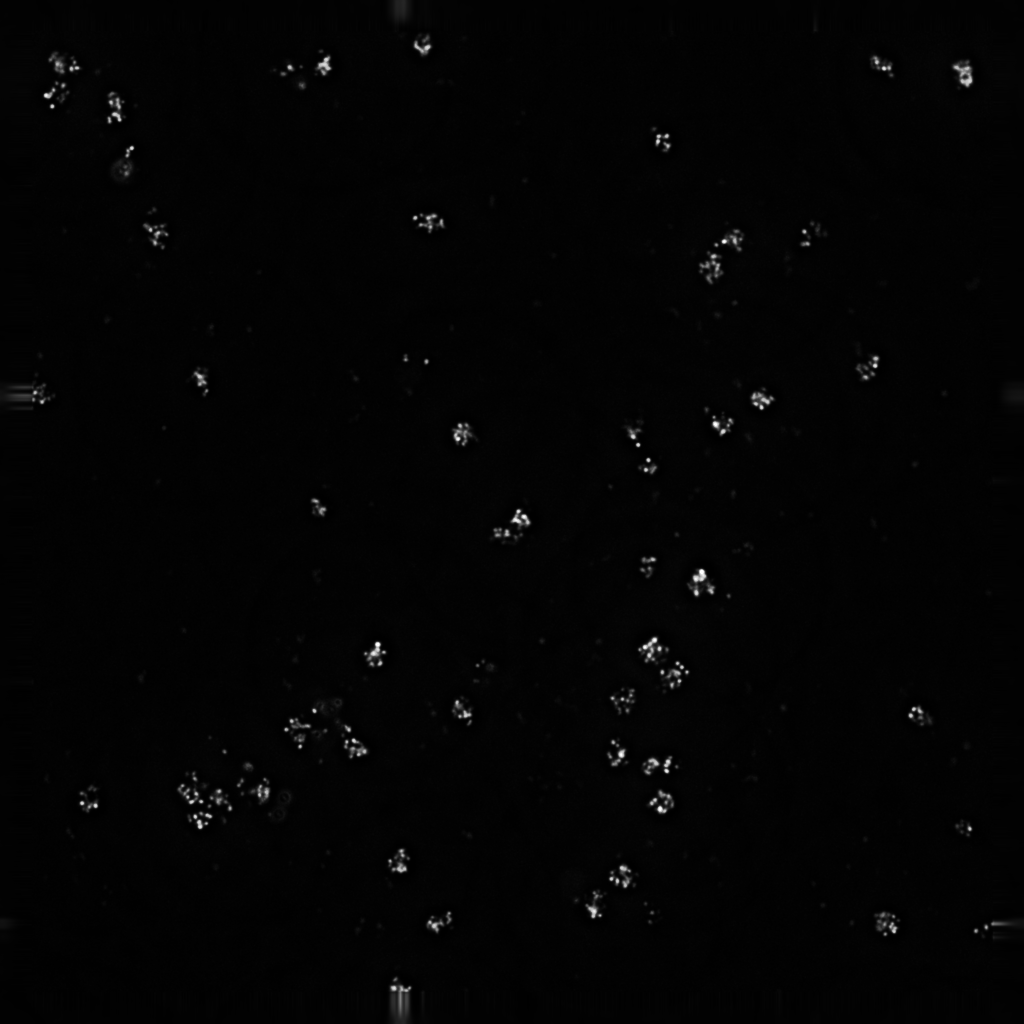

Supplement: Supplementary file 7 — Source data Fig. 5 [file 44318_2025_580_MOESM7_ESM.zip › Figure 5/A/-Rad18_40.tif]

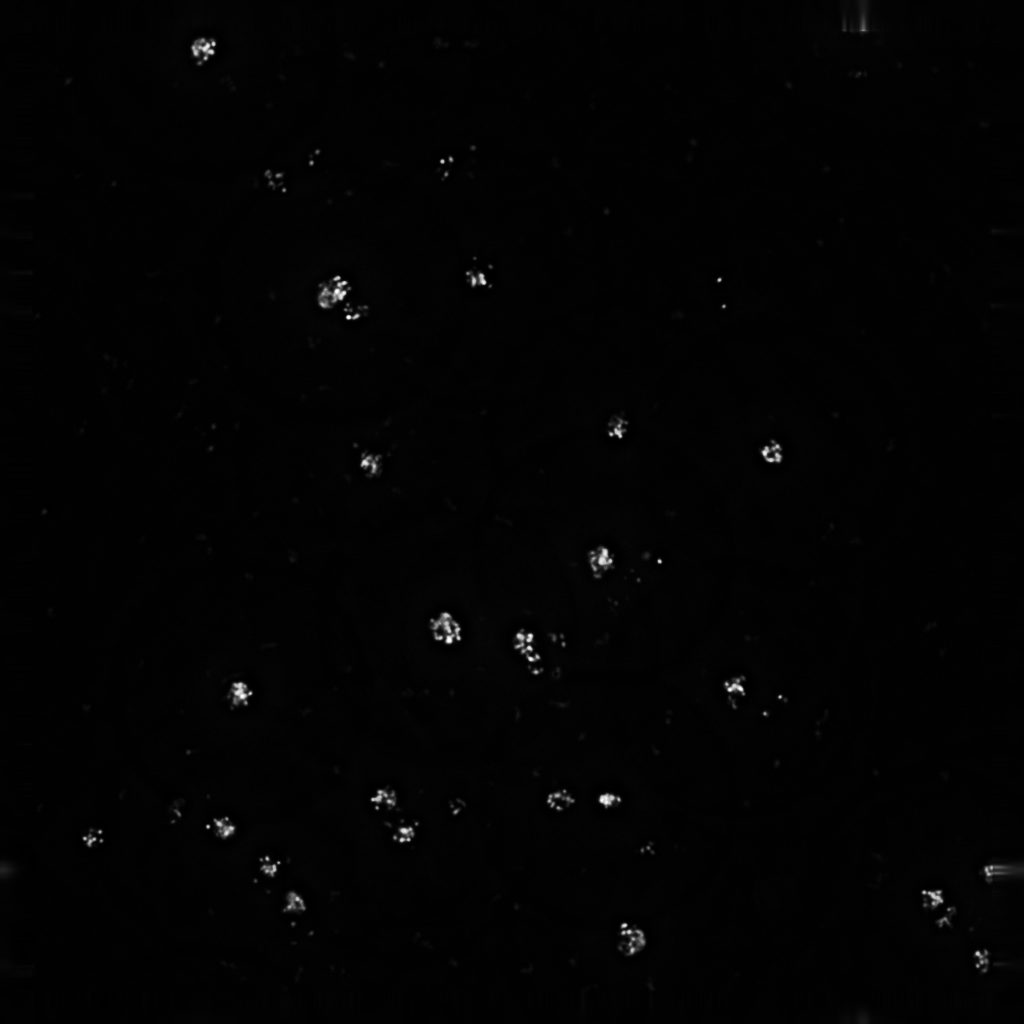

Supplement: Supplementary file 7 — Source data Fig. 5 [file 44318_2025_580_MOESM7_ESM.zip › Figure 5/A/-Rad18_80.tif]

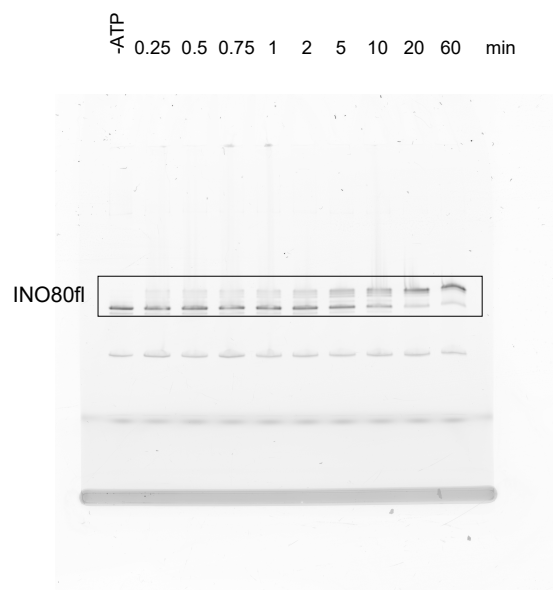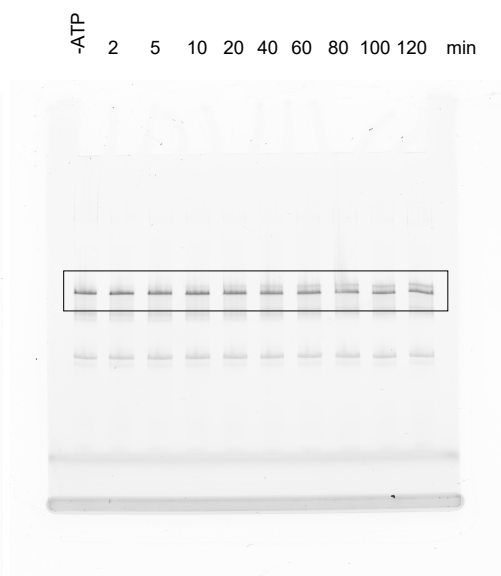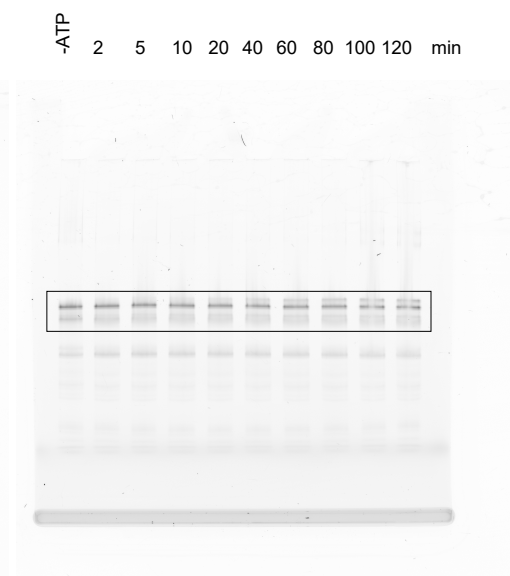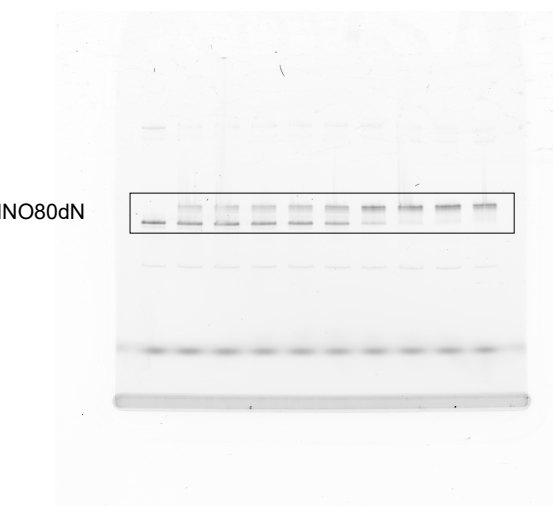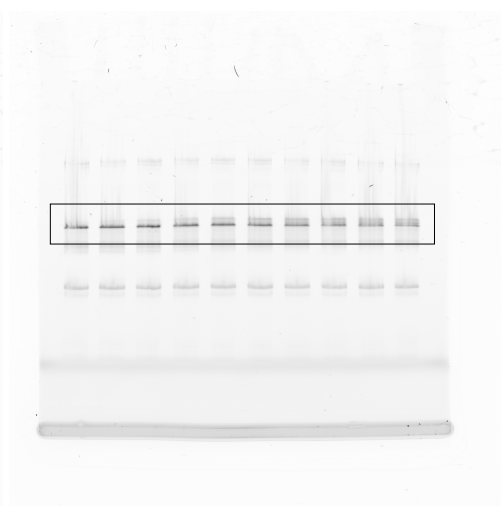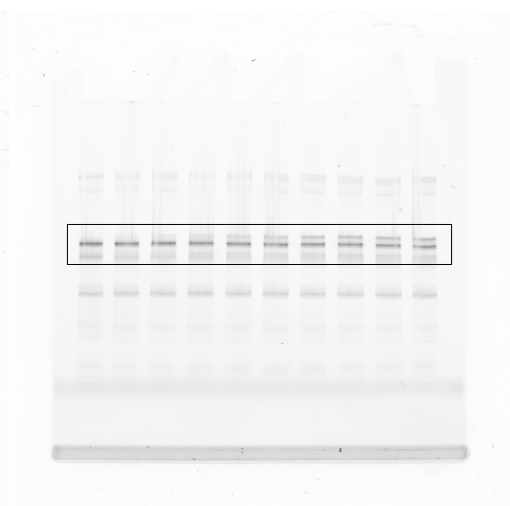

blunt

5'-overhang

3'-overhang

Supplement: Supplementary file 7 — Source data Fig. 5 [file 44318_2025_580_MOESM7_ESM.zip › Figure 5/D/Fig5D_gels.pdf]

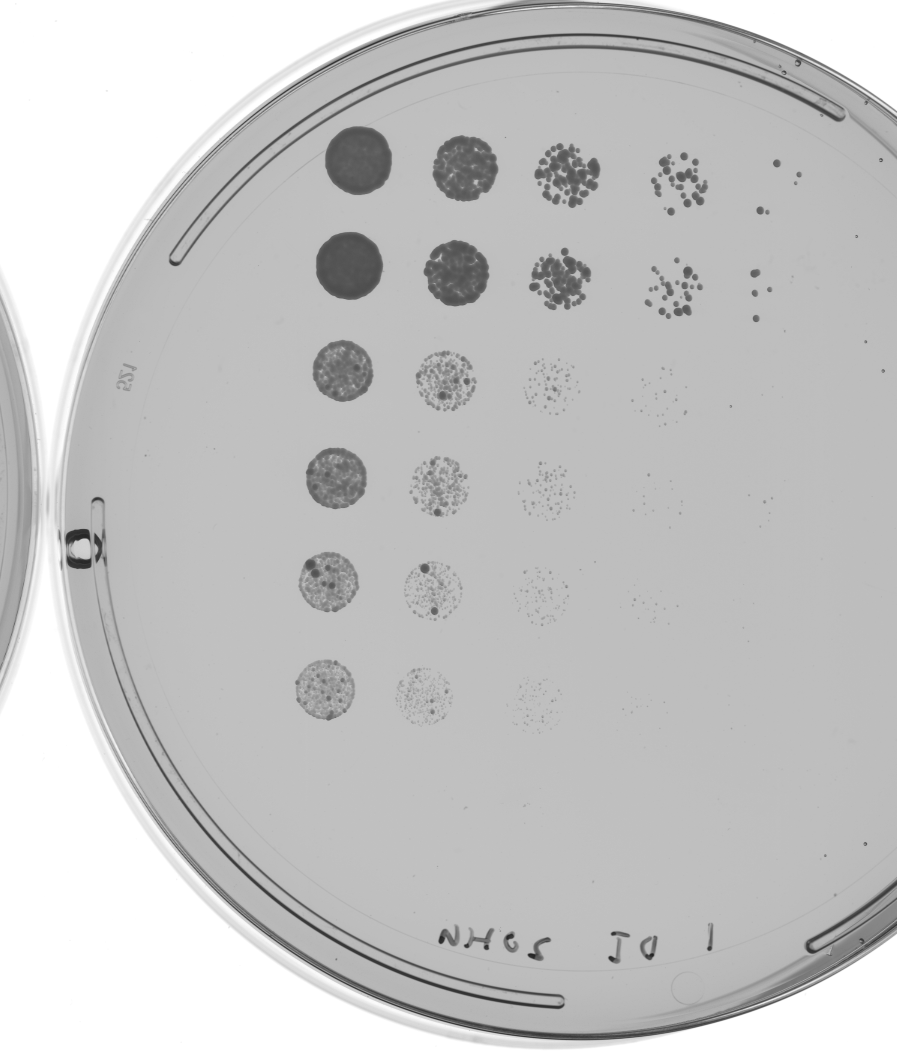

Supplement: Supplementary file 8 — EV Figure Source Data [file 44318_2025_580_MOESM8_ESM.zip › SD EV figures/Figure EV1/A/+IAA +Dox HU 20mM.tif]

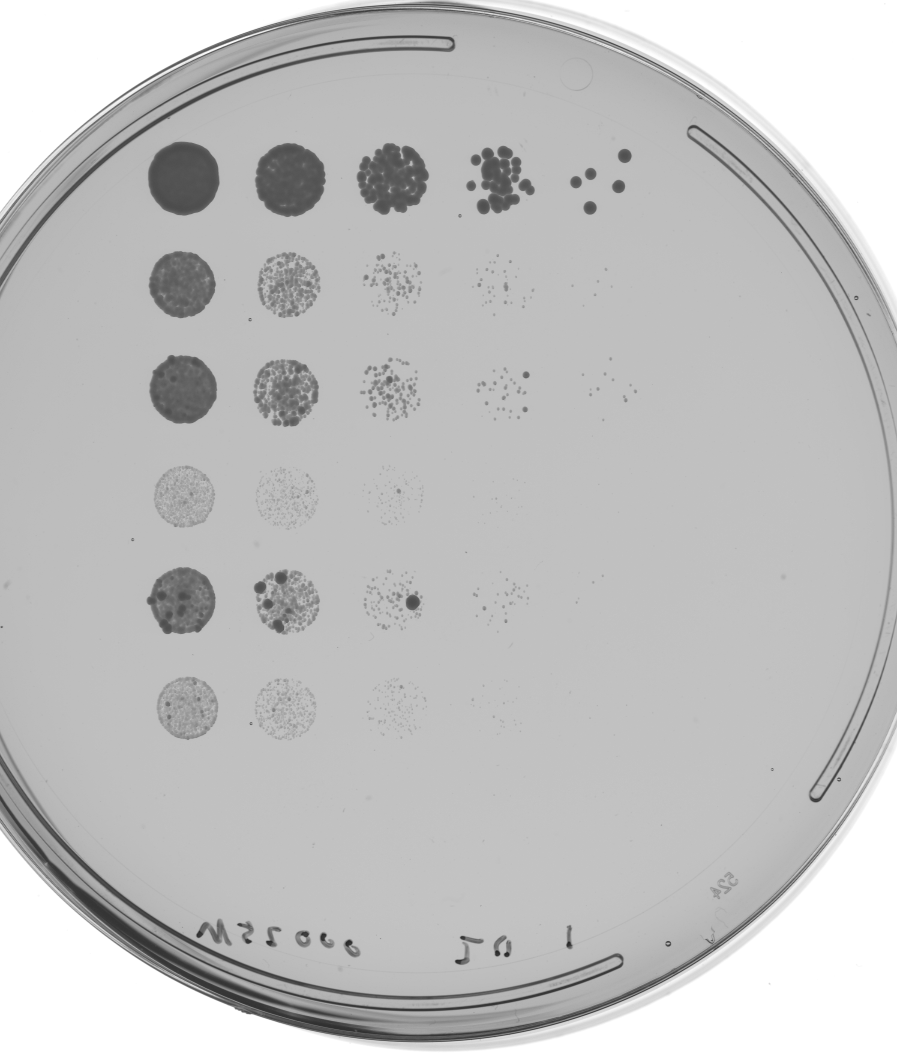

Supplement: Supplementary file 8 — EV Figure Source Data [file 44318_2025_580_MOESM8_ESM.zip › SD EV figures/Figure EV1/A/+IAA +Dox MMS 00025.tif]

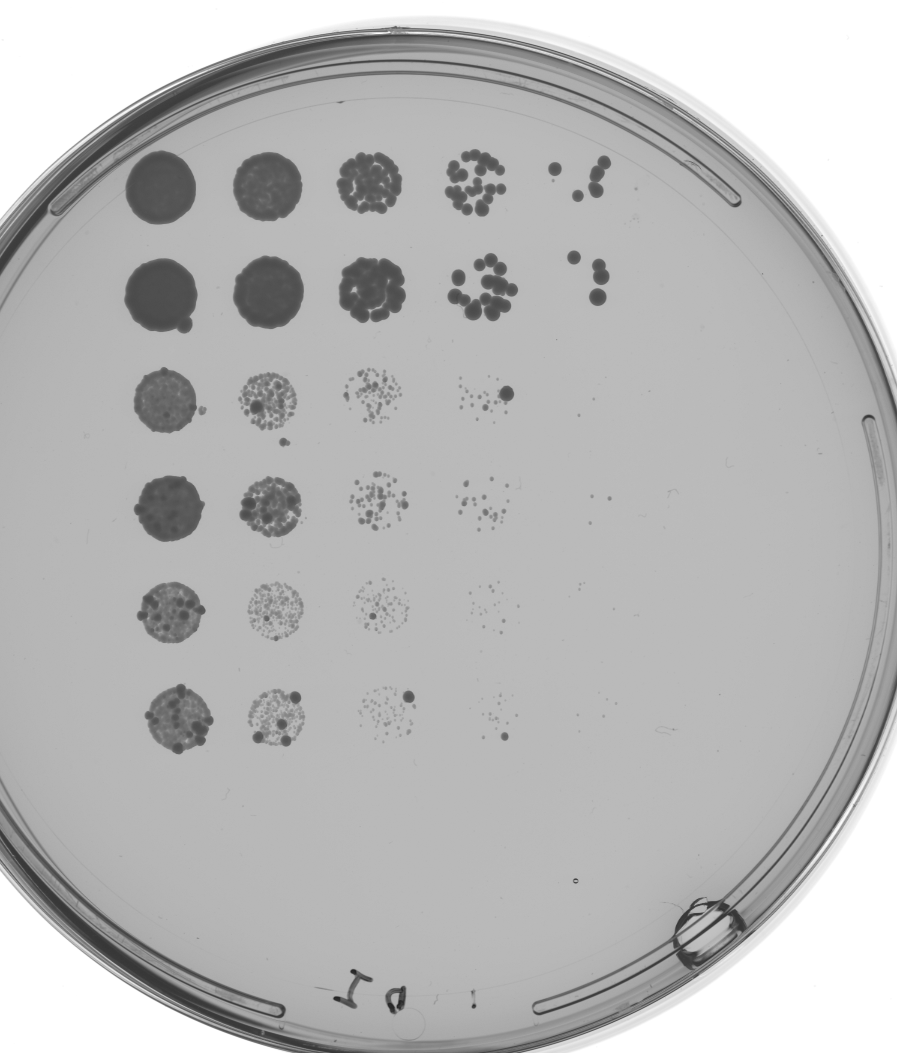

Supplement: Supplementary file 8 — EV Figure Source Data [file 44318_2025_580_MOESM8_ESM.zip › SD EV figures/Figure EV1/A/+IAA +Dox untreated.tif]

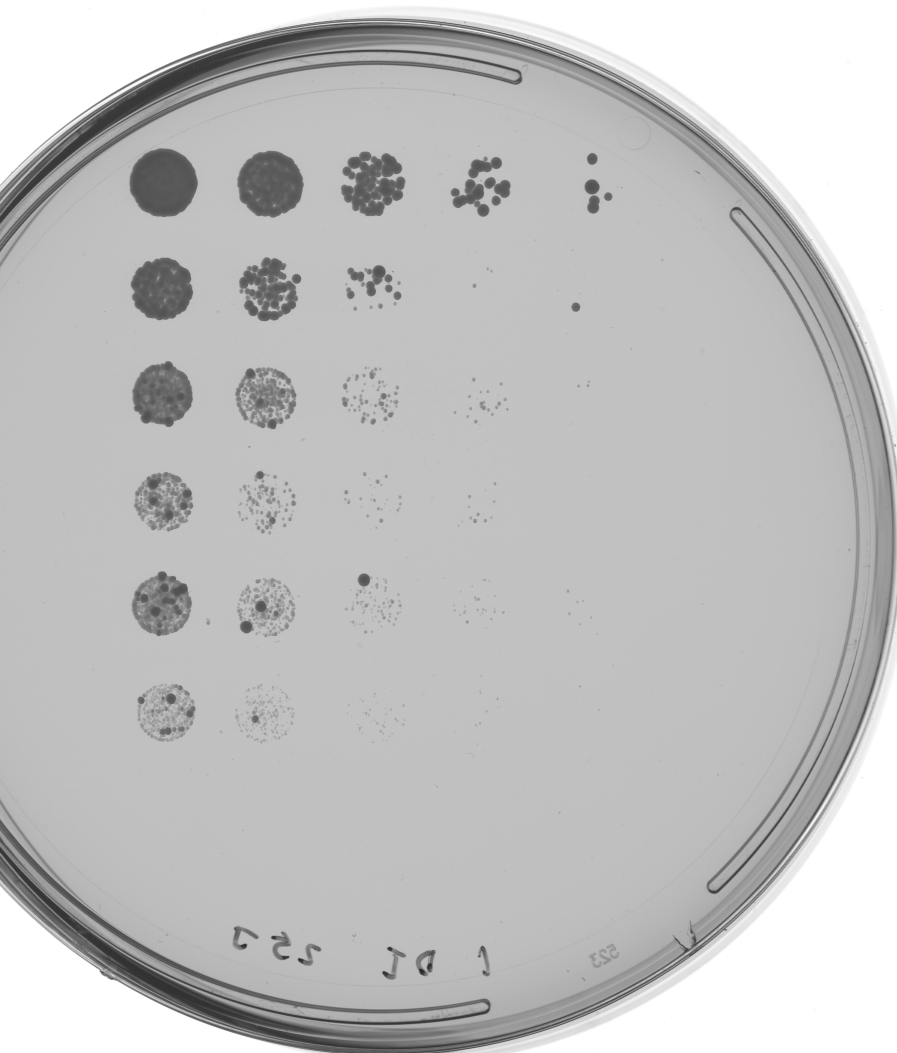

Supplement: Supplementary file 8 — EV Figure Source Data [file 44318_2025_580_MOESM8_ESM.zip › SD EV figures/Figure EV1/A/+IAA +Dox UV 25J.tif]

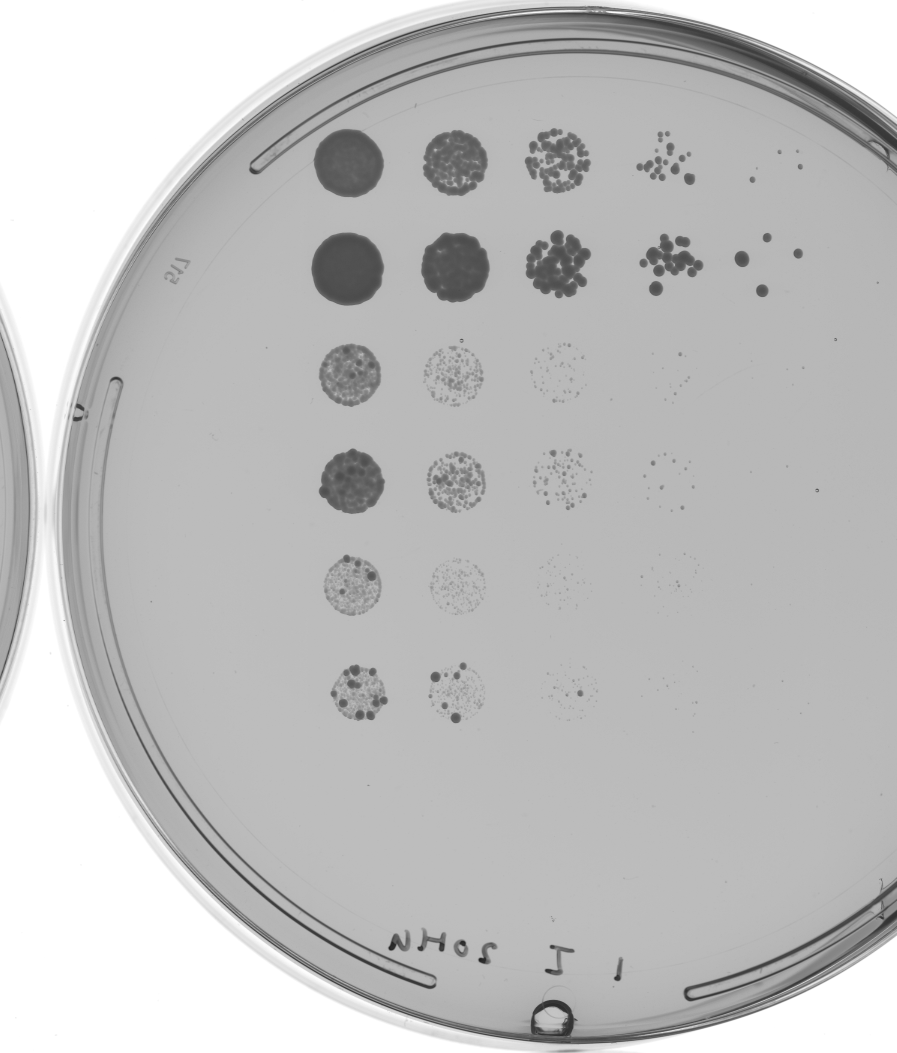

Supplement: Supplementary file 8 — EV Figure Source Data [file 44318_2025_580_MOESM8_ESM.zip › SD EV figures/Figure EV1/A/+IAA -Dox HU 20mM.tif]

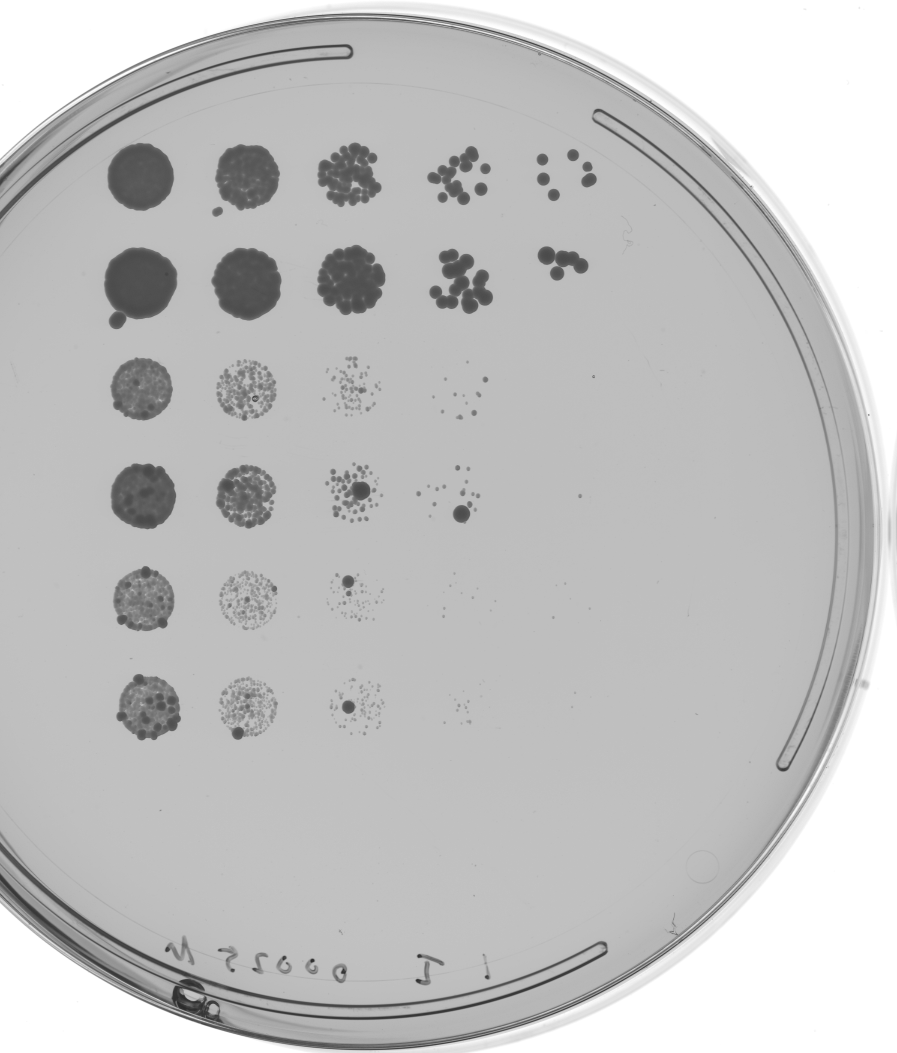

Supplement: Supplementary file 8 — EV Figure Source Data [file 44318_2025_580_MOESM8_ESM.zip › SD EV figures/Figure EV1/A/+IAA -Dox MMS 00025.tif]

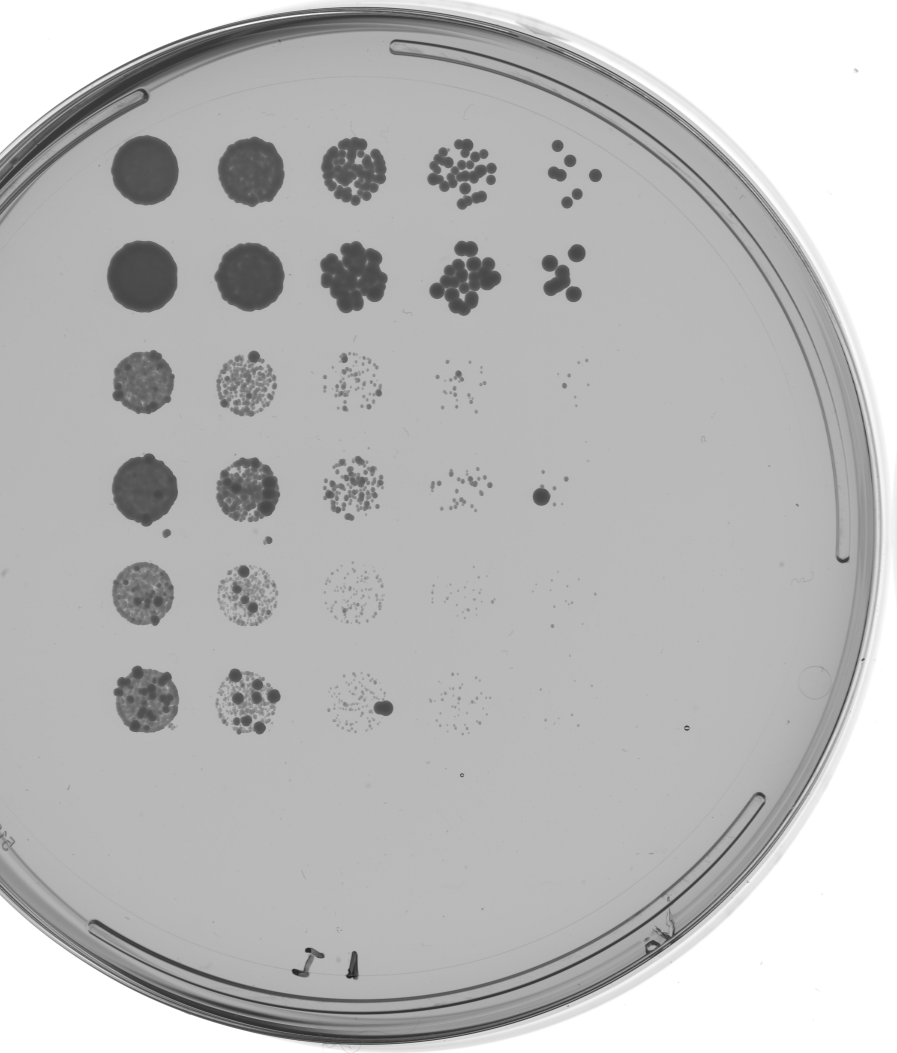

Supplement: Supplementary file 8 — EV Figure Source Data [file 44318_2025_580_MOESM8_ESM.zip › SD EV figures/Figure EV1/A/+IAA -Dox untreated.tif]

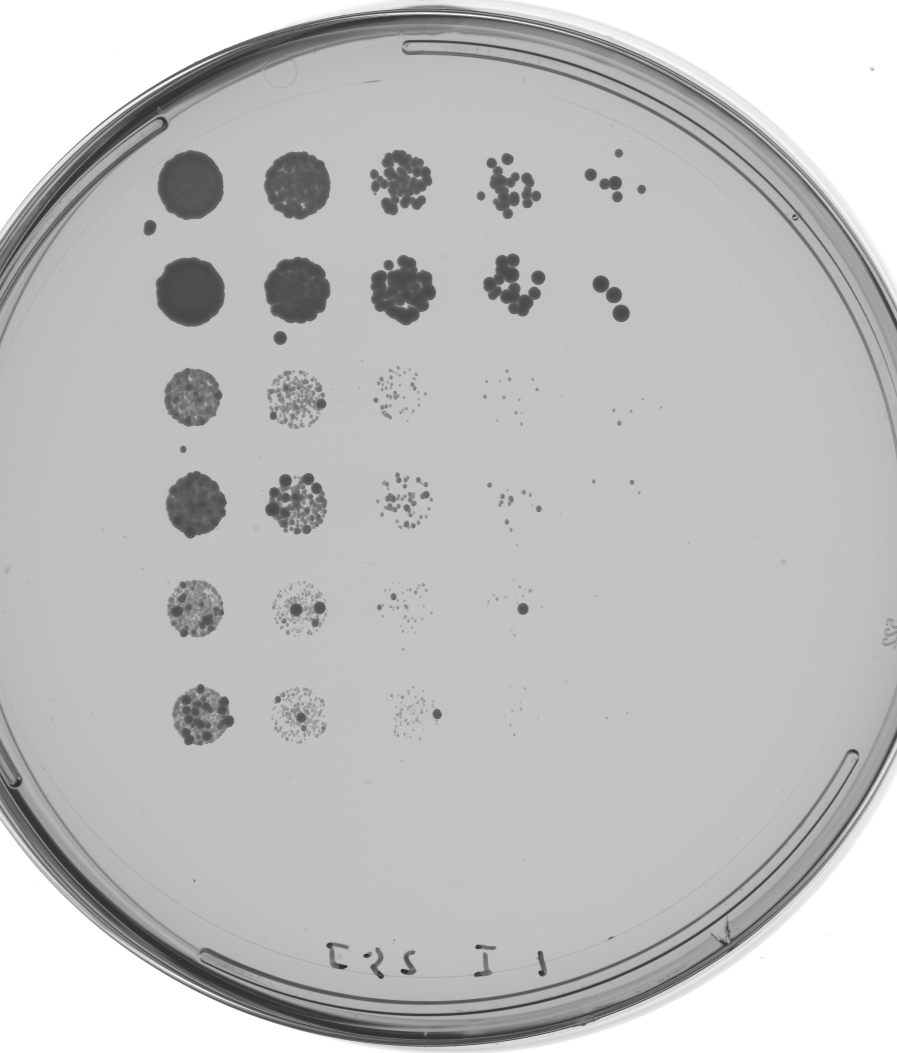

Supplement: Supplementary file 8 — EV Figure Source Data [file 44318_2025_580_MOESM8_ESM.zip › SD EV figures/Figure EV1/A/+IAA -Dox UV 25J.tif]

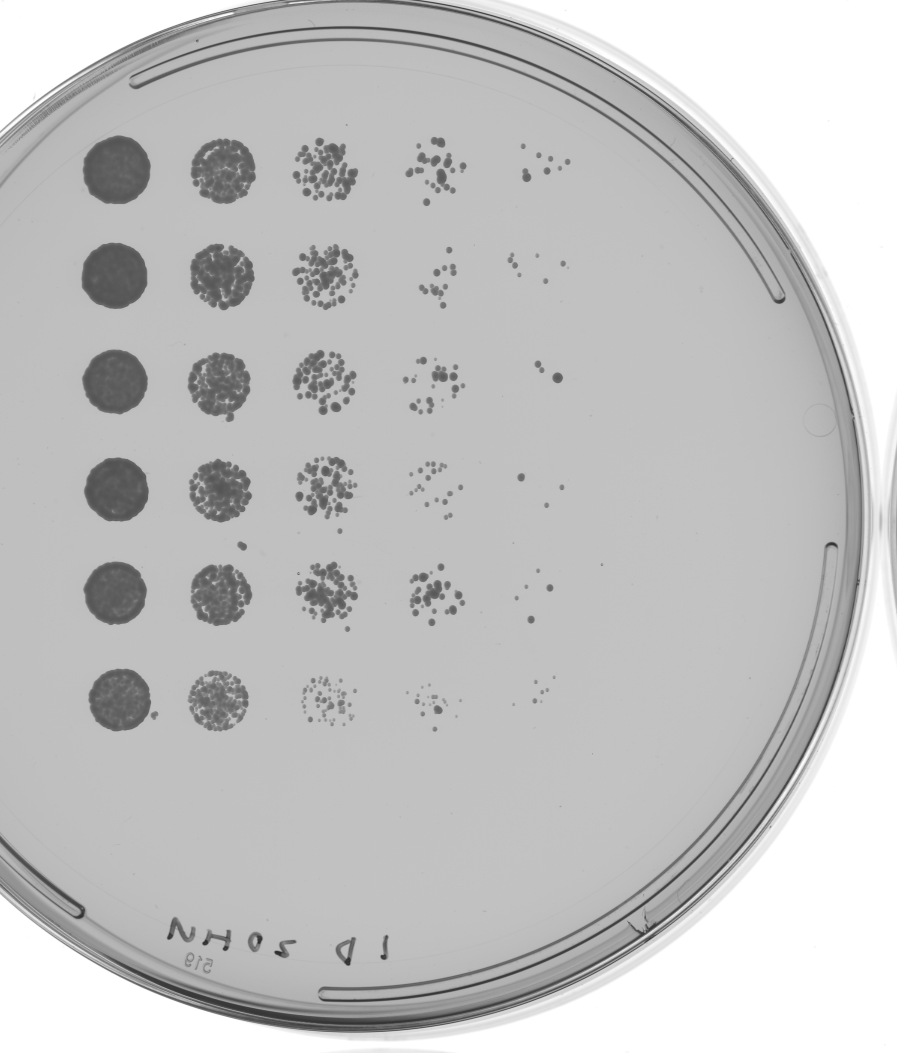

Supplement: Supplementary file 8 — EV Figure Source Data [file 44318_2025_580_MOESM8_ESM.zip › SD EV figures/Figure EV1/A/-IAA +Dox HU 20mM.tif]

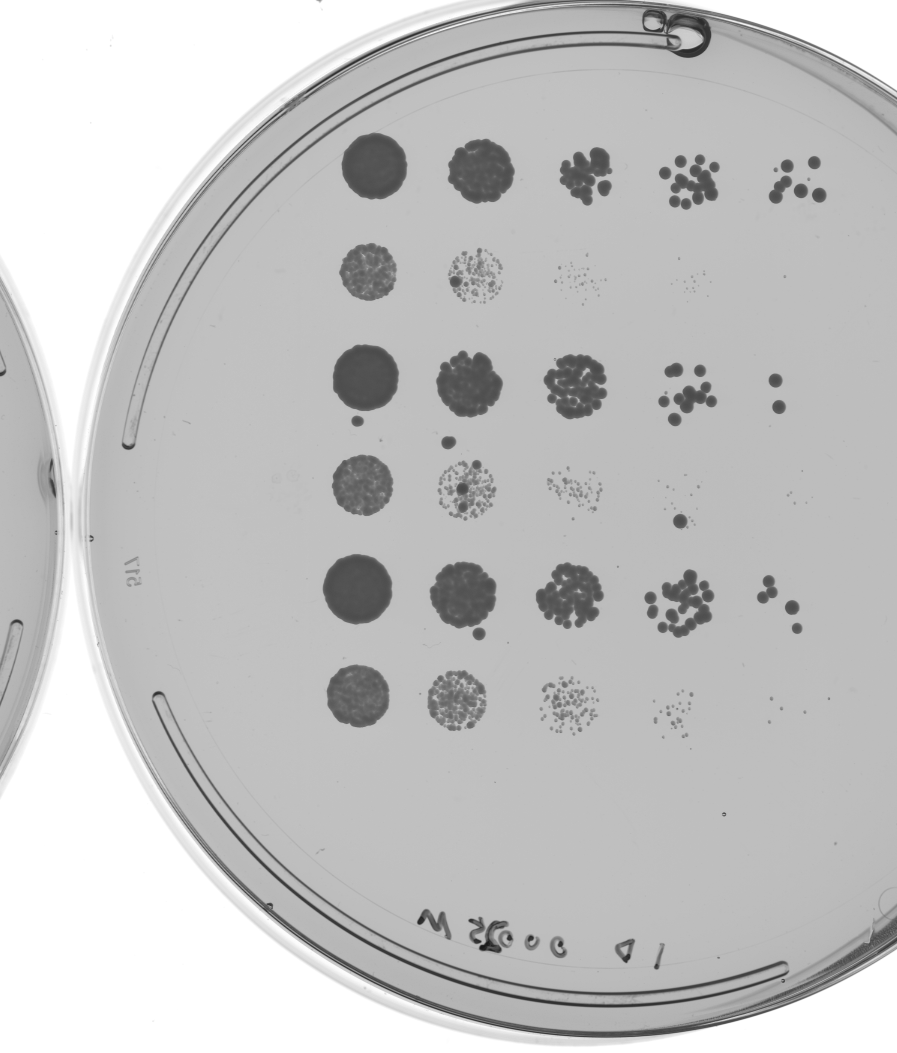

Supplement: Supplementary file 8 — EV Figure Source Data [file 44318_2025_580_MOESM8_ESM.zip › SD EV figures/Figure EV1/A/-IAA +Dox MMS 00025.tif]

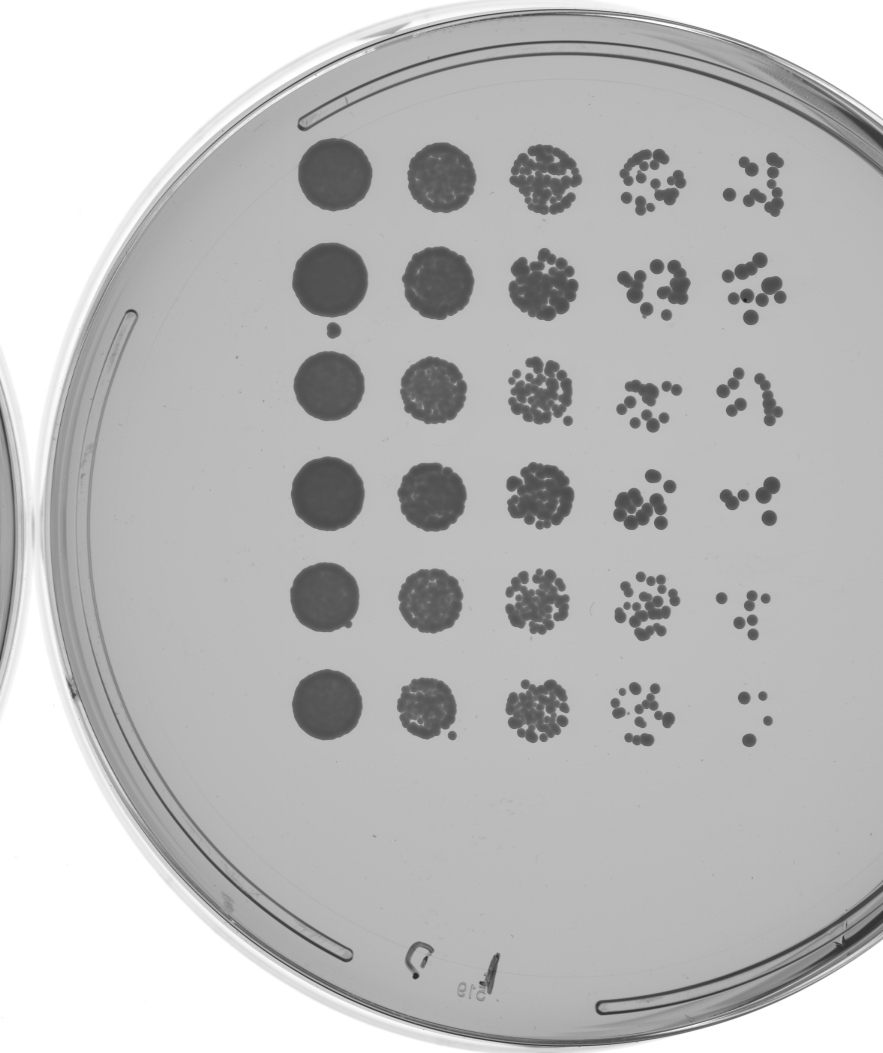

Supplement: Supplementary file 8 — EV Figure Source Data [file 44318_2025_580_MOESM8_ESM.zip › SD EV figures/Figure EV1/A/-IAA +Dox untreated.tif]

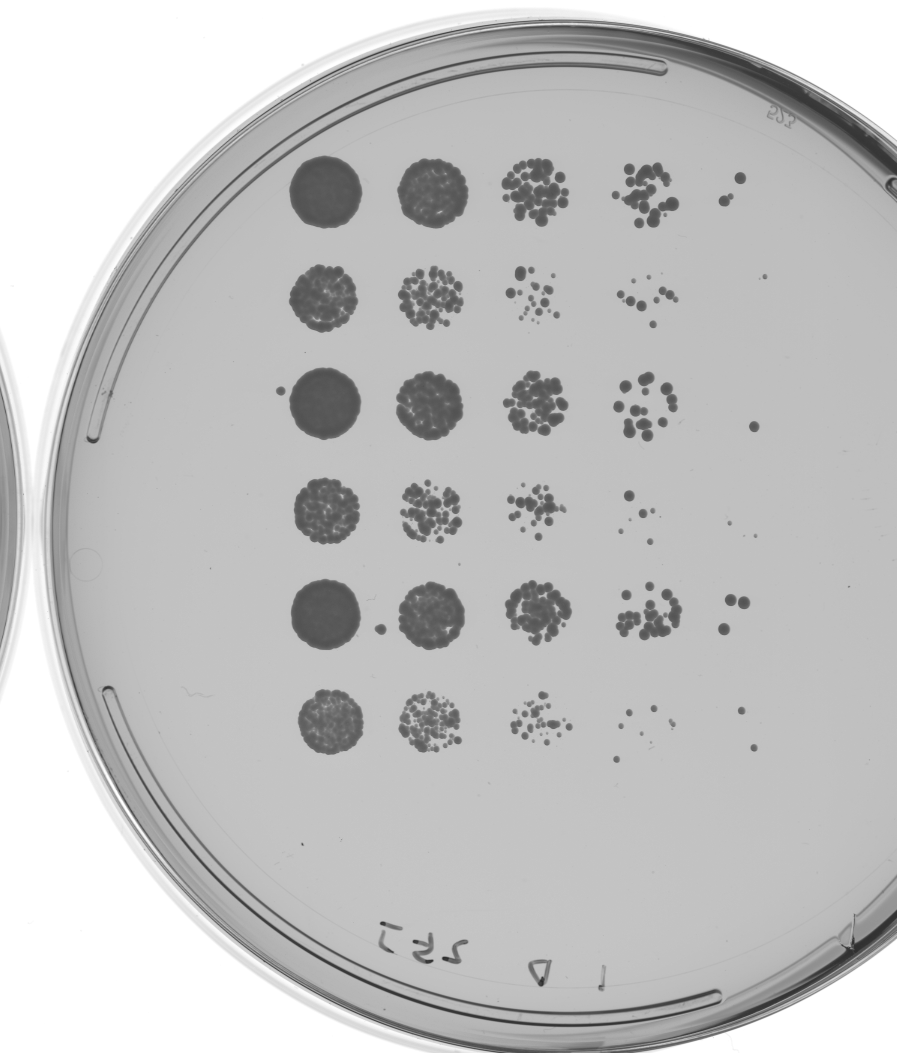

Supplement: Supplementary file 8 — EV Figure Source Data [file 44318_2025_580_MOESM8_ESM.zip › SD EV figures/Figure EV1/A/-IAA +Dox UV 25J.tif]

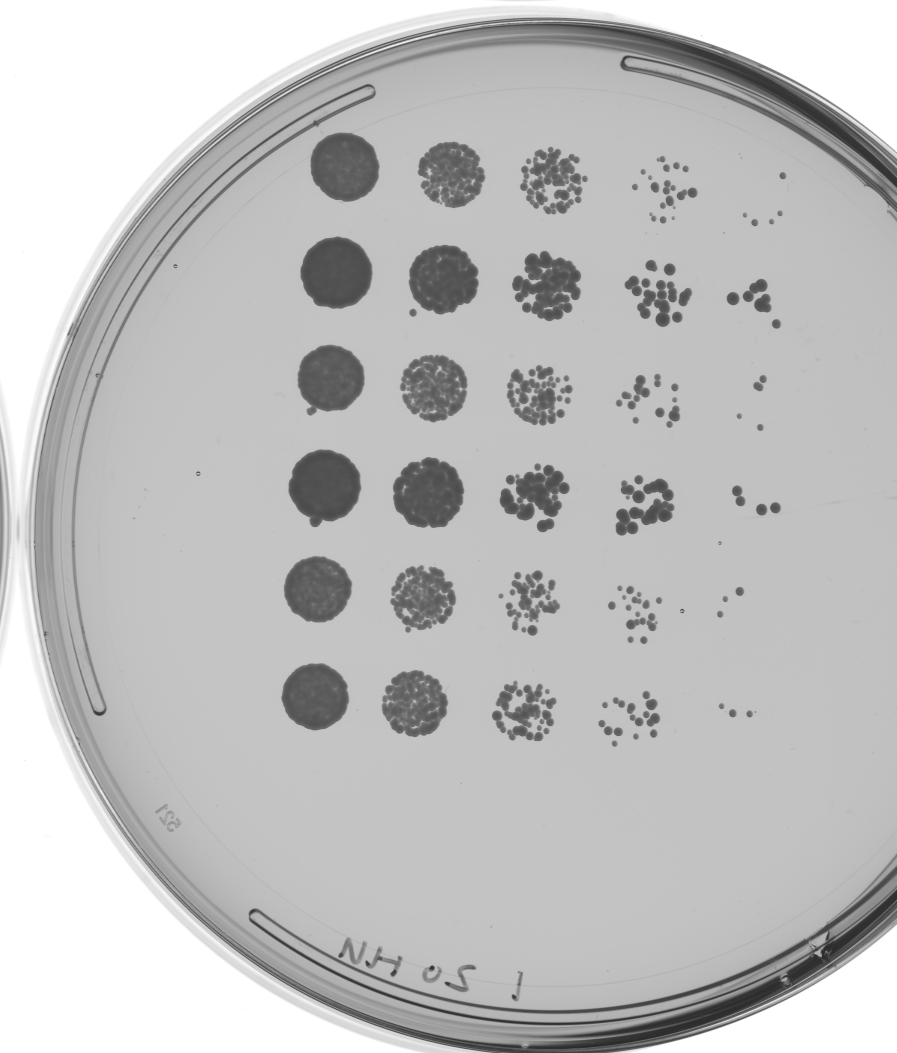

Supplement: Supplementary file 8 — EV Figure Source Data [file 44318_2025_580_MOESM8_ESM.zip › SD EV figures/Figure EV1/A/-IAA -Dox HU 20mM.tif]

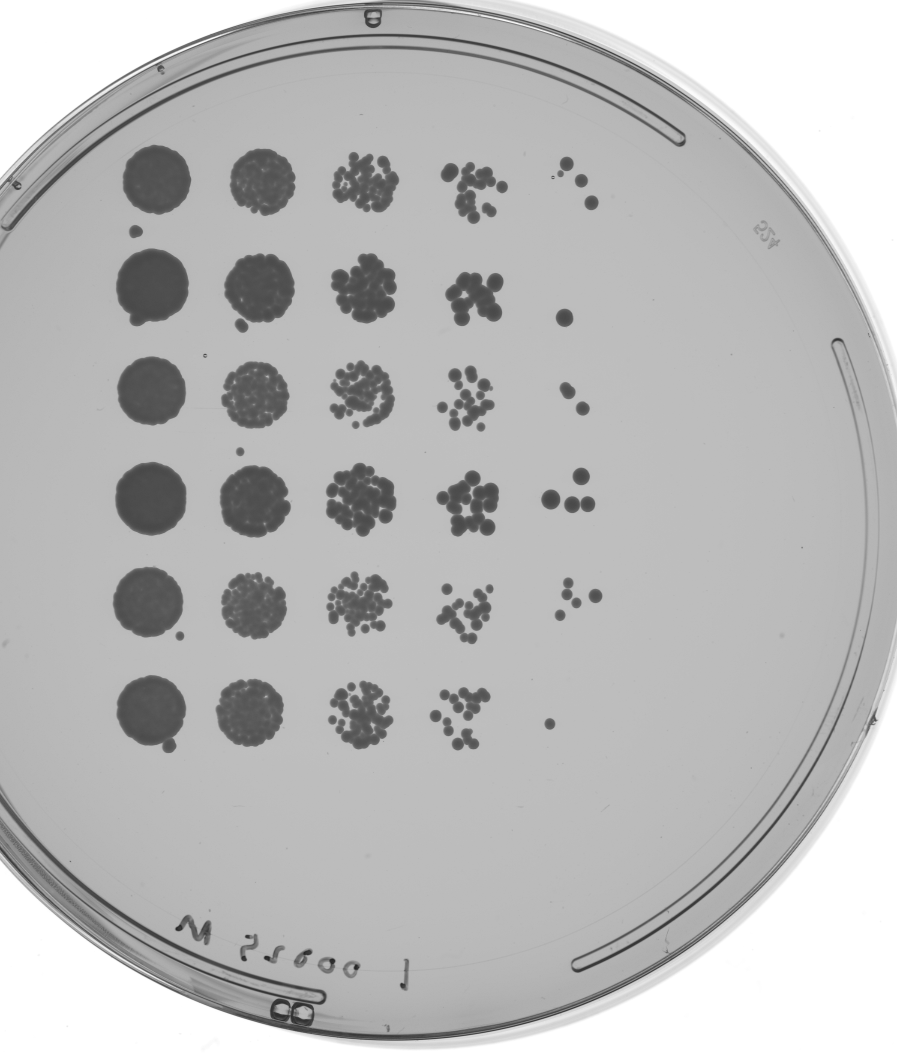

Supplement: Supplementary file 8 — EV Figure Source Data [file 44318_2025_580_MOESM8_ESM.zip › SD EV figures/Figure EV1/A/-IAA -Dox MMS 00025.tif]

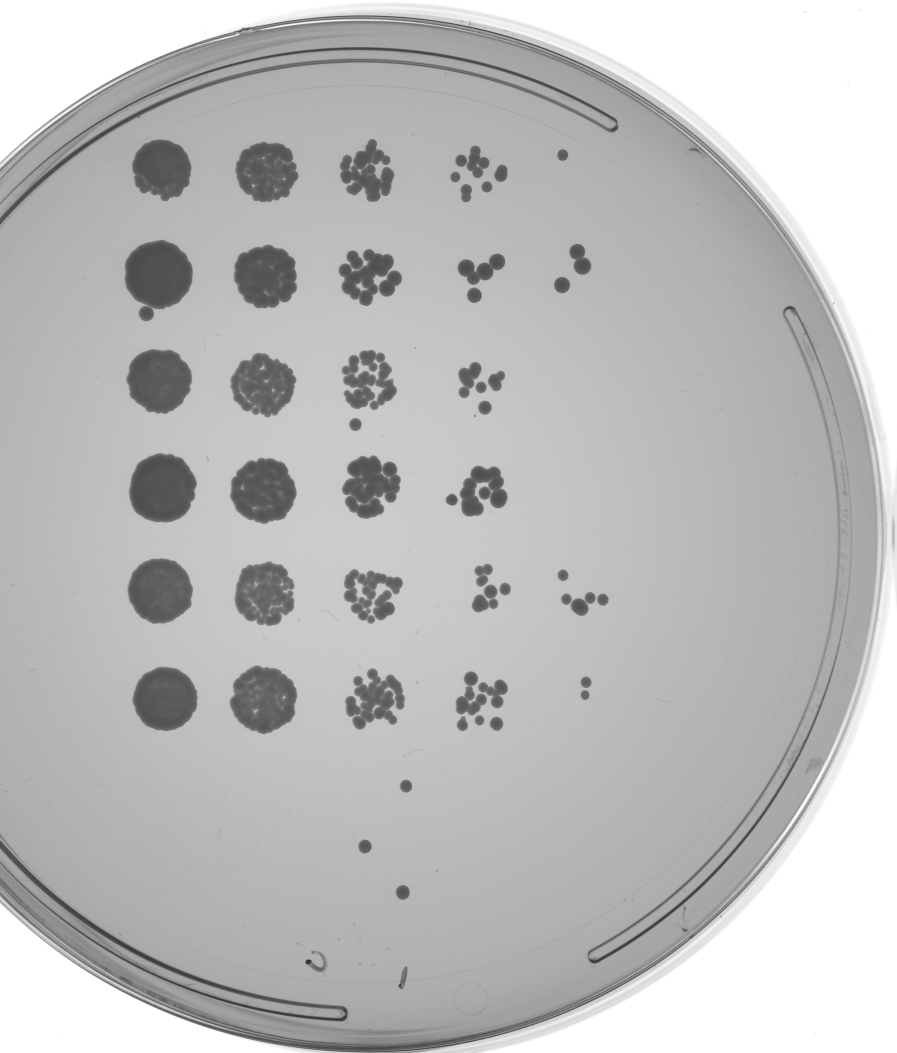

Supplement: Supplementary file 8 — EV Figure Source Data [file 44318_2025_580_MOESM8_ESM.zip › SD EV figures/Figure EV1/A/-IAA -Dox untreated.tif]

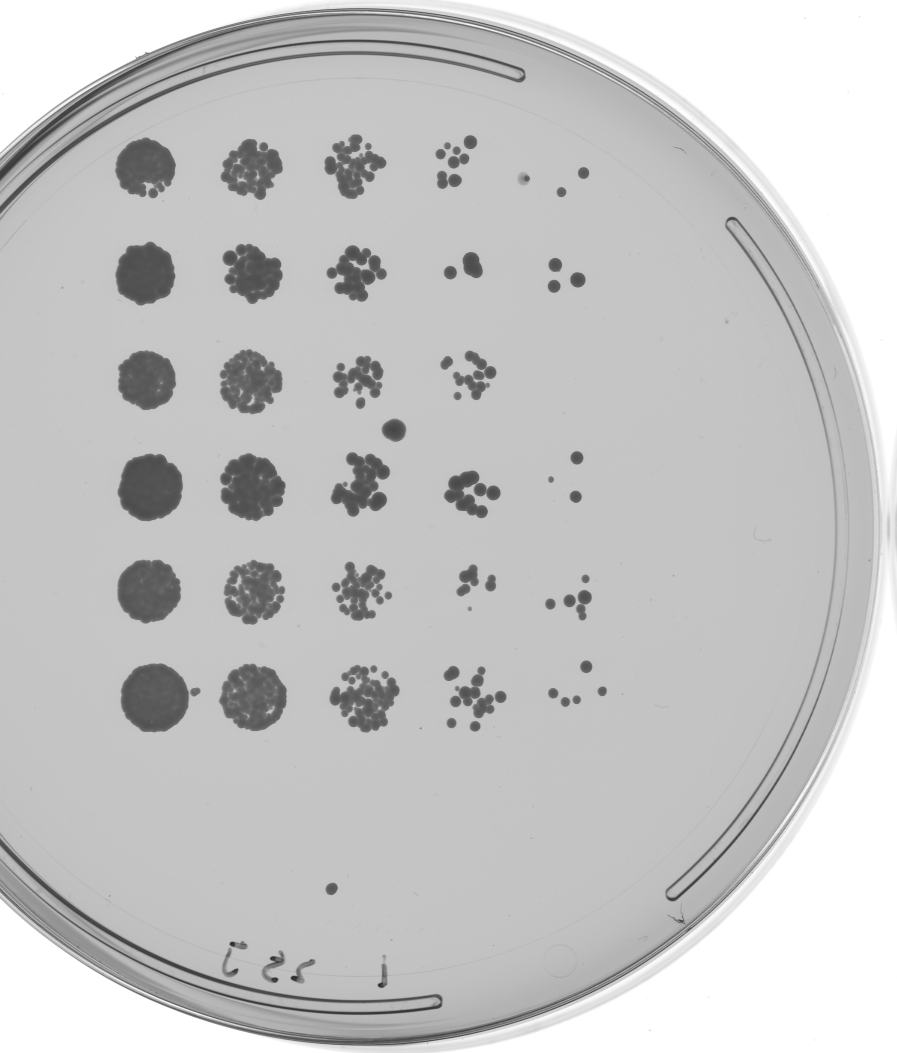

Supplement: Supplementary file 8 — EV Figure Source Data [file 44318_2025_580_MOESM8_ESM.zip › SD EV figures/Figure EV1/A/-IAA -Dox UV 25J.tif]

|     | WT |   |   |   | Ino80 <sup>ΔD</sup> -Flag |   |   |   | Arp5 <sup>ΔD</sup> -Flag |   |   |   | rad18Δ |   |     |
|-----|----|---|---|---|---------------------------|---|---|---|--------------------------|---|---|---|--------|---|-----|
| Dox | -  | - | - | + | +                         | - | - | + | -                        | - | + | + | -      | - |     |
| IAA | -  | - | + | - | +                         | - | + | - | -                        | + | - | + | -      | + | kDa |

Flag

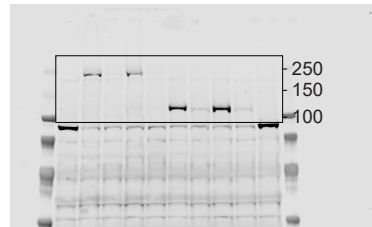

Rad18

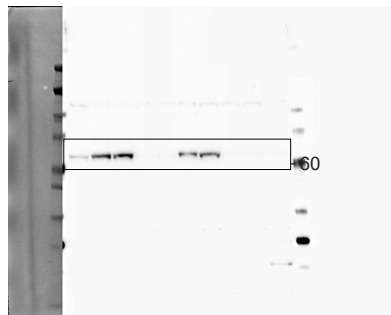

α-Tubulin

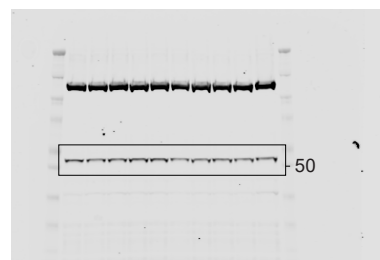

Supplement: Supplementary file 8 — EV Figure Source Data [file 44318_2025_580_MOESM8_ESM.zip › SD EV figures/Figure EV1/B/Western tetRad18 Ino80aid Arp5aid.pdf]

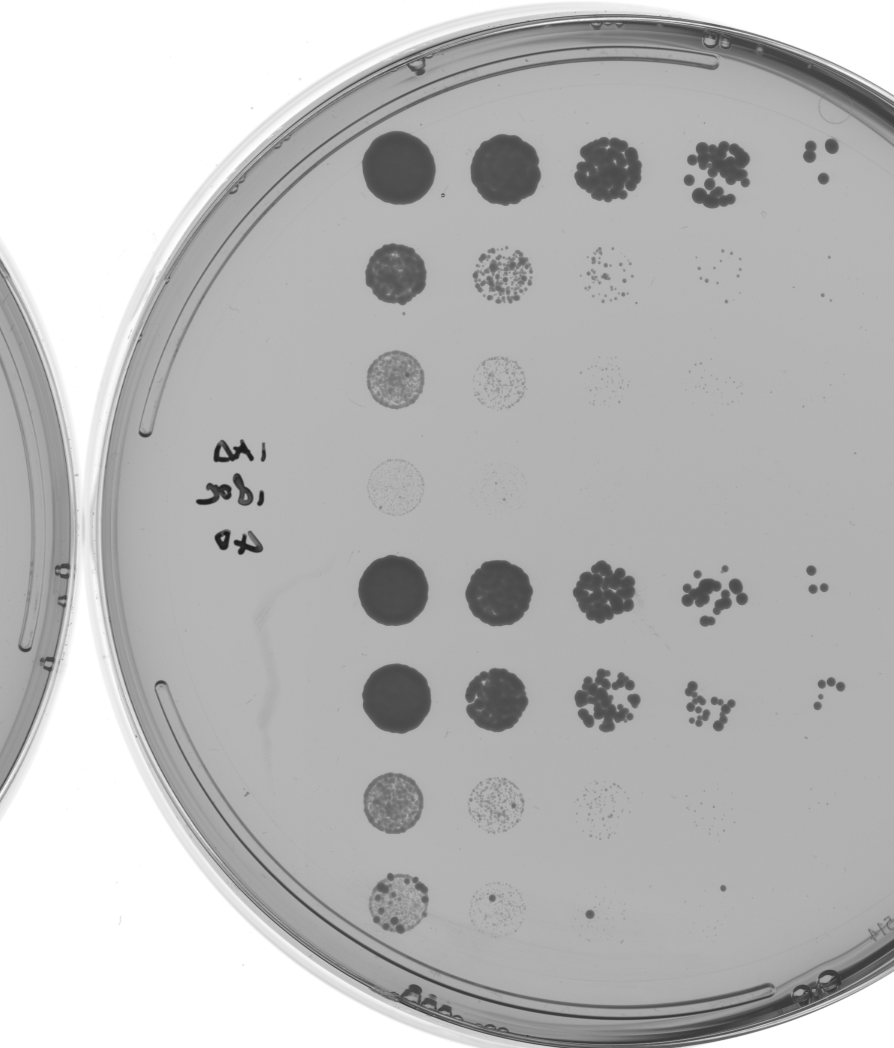

Supplement: Supplementary file 8 — EV Figure Source Data [file 44318_2025_580_MOESM8_ESM.zip › SD EV figures/Figure EV1/C/+IAA 18C.tif]

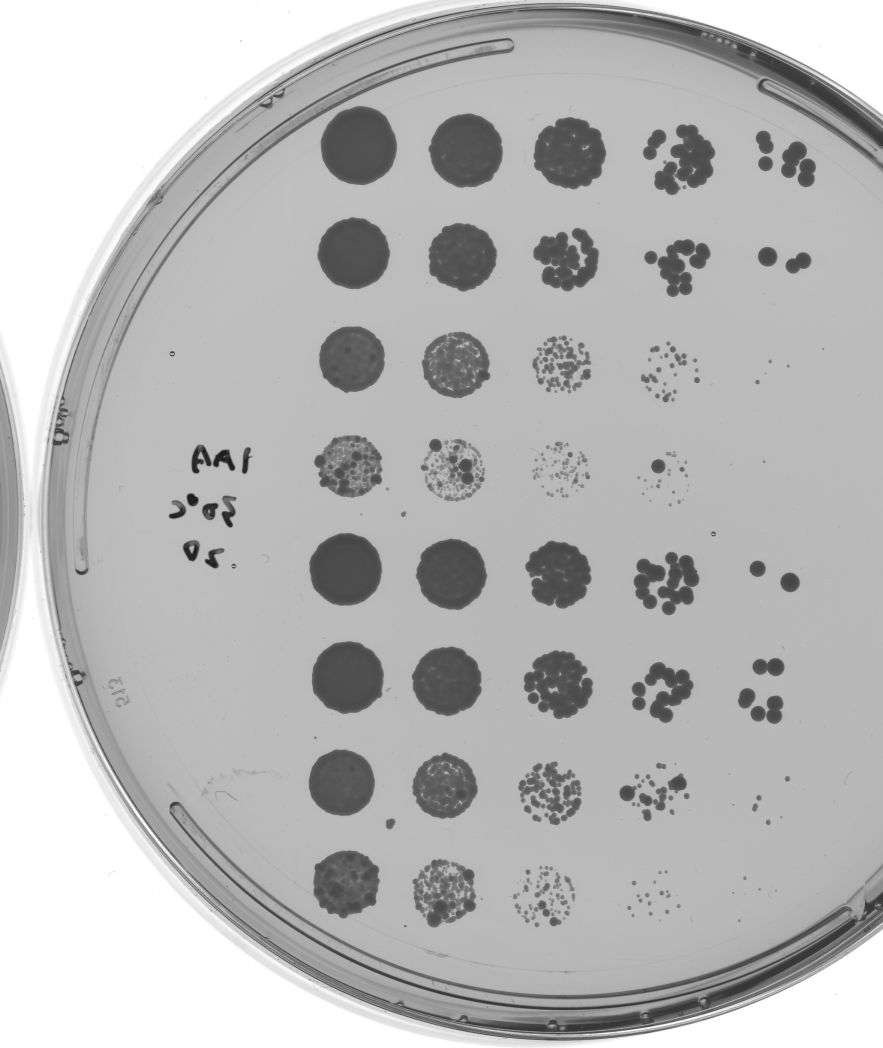

Supplement: Supplementary file 8 — EV Figure Source Data [file 44318_2025_580_MOESM8_ESM.zip › SD EV figures/Figure EV1/C/+IAA 30C.tif]

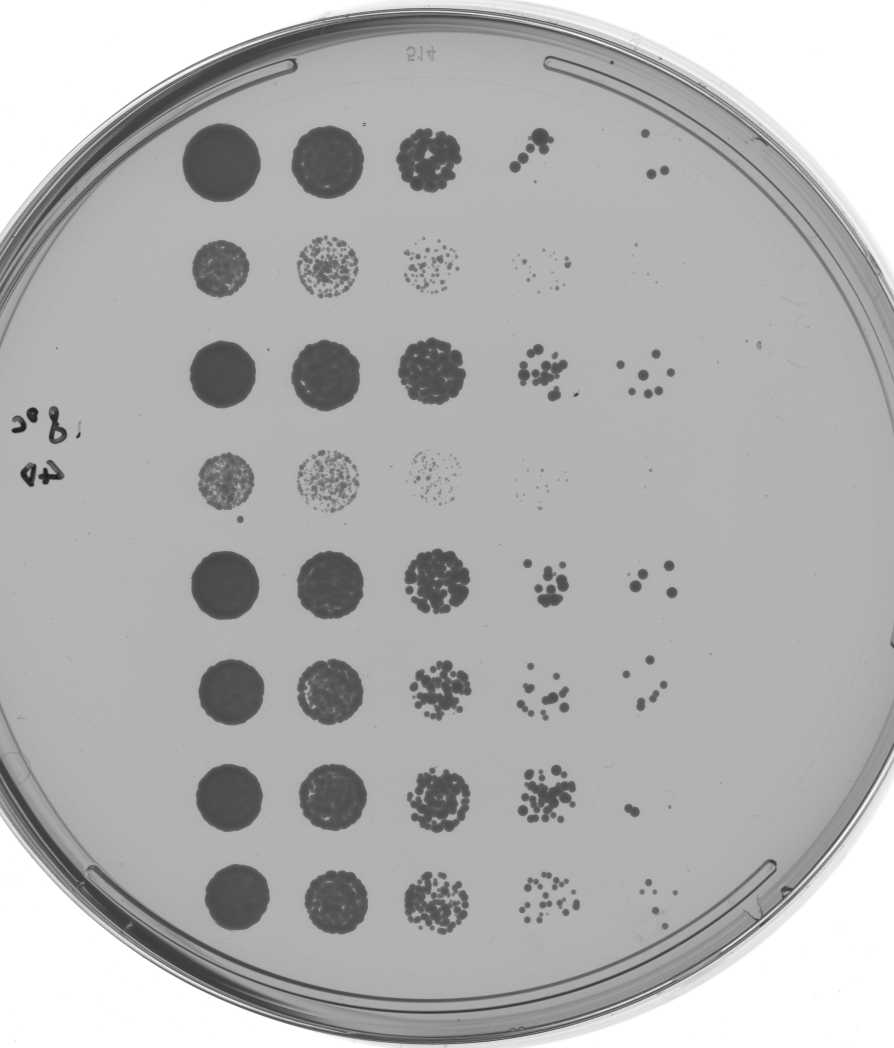

Supplement: Supplementary file 8 — EV Figure Source Data [file 44318_2025_580_MOESM8_ESM.zip › SD EV figures/Figure EV1/C/-IAA 18C.tif]

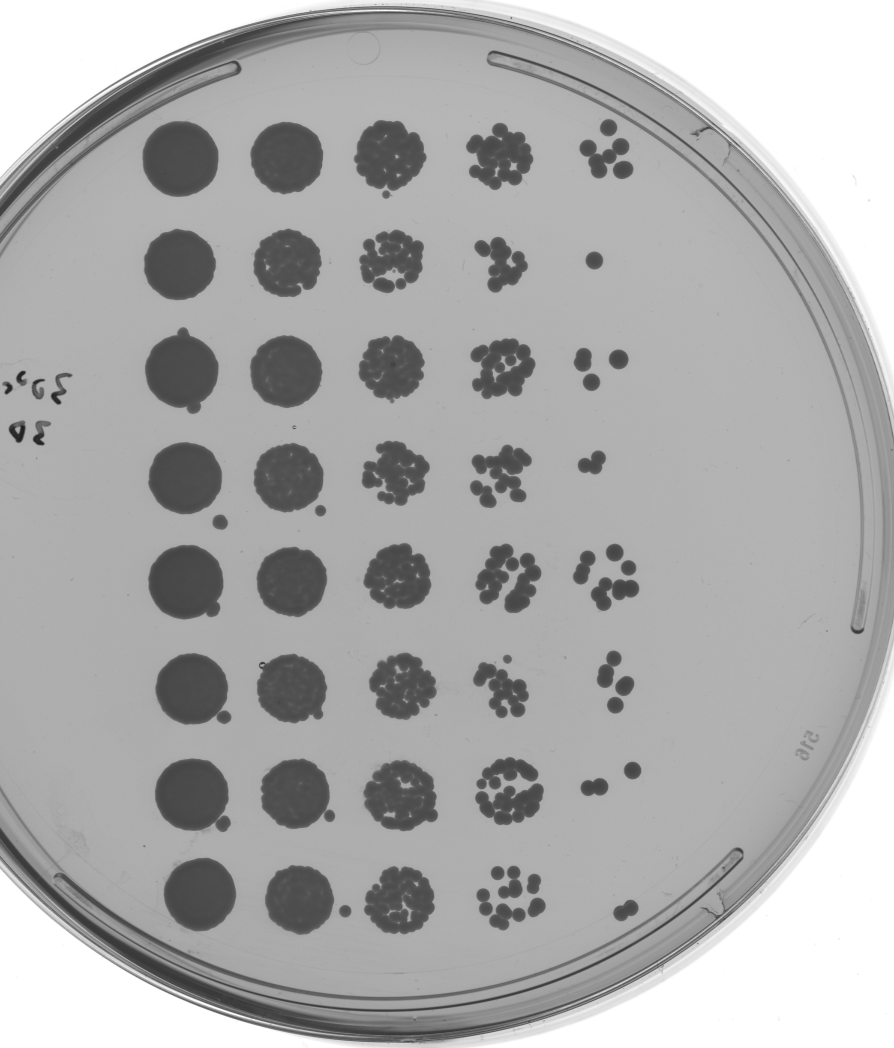

Supplement: Supplementary file 8 — EV Figure Source Data [file 44318_2025_580_MOESM8_ESM.zip › SD EV figures/Figure EV1/C/-IAA 30C.tif]

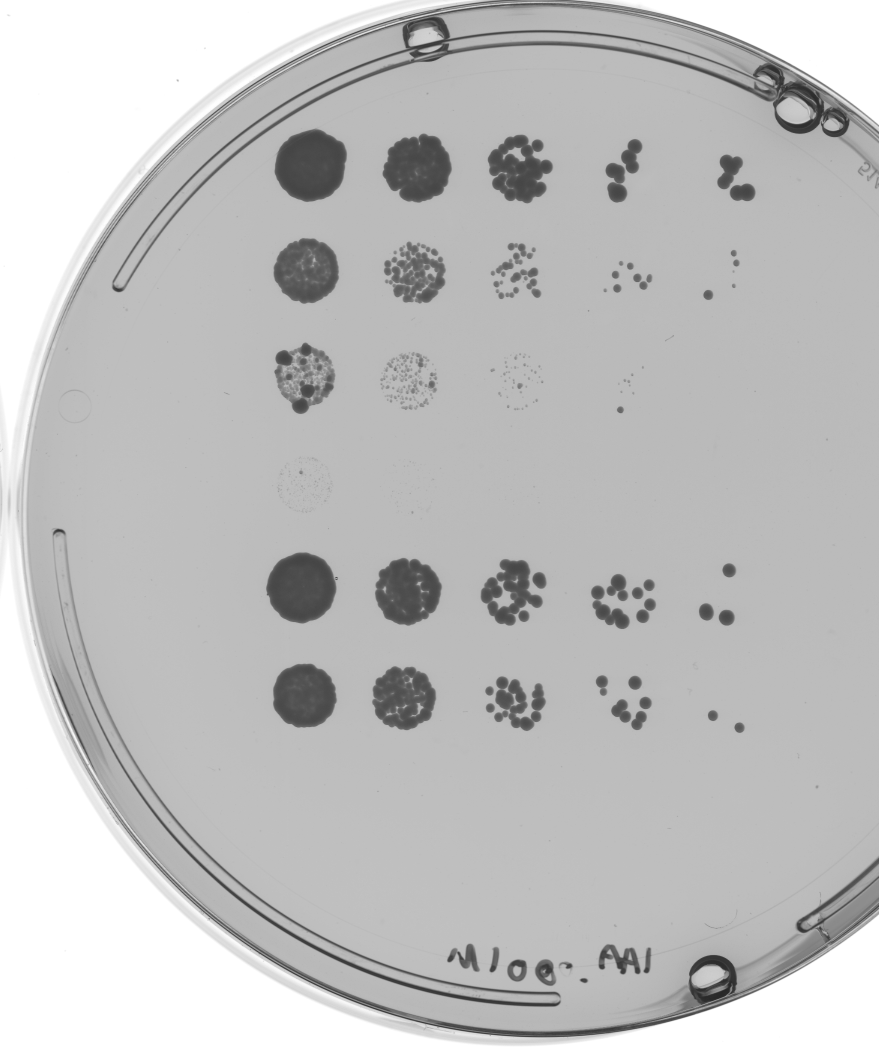

Supplement: Supplementary file 8 — EV Figure Source Data [file 44318_2025_580_MOESM8_ESM.zip › SD EV figures/Figure EV1/D/+IAA MMS 001.tif]

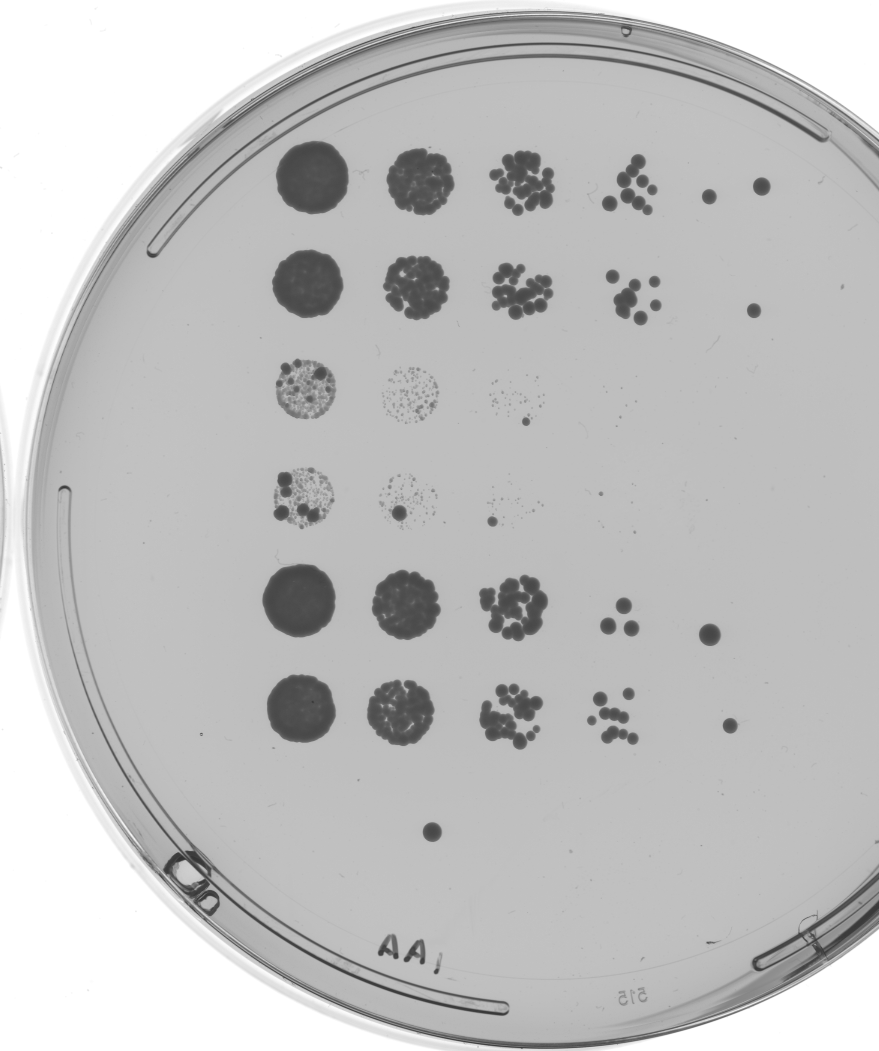

Supplement: Supplementary file 8 — EV Figure Source Data [file 44318_2025_580_MOESM8_ESM.zip › SD EV figures/Figure EV1/D/+IAA untreated.tif]

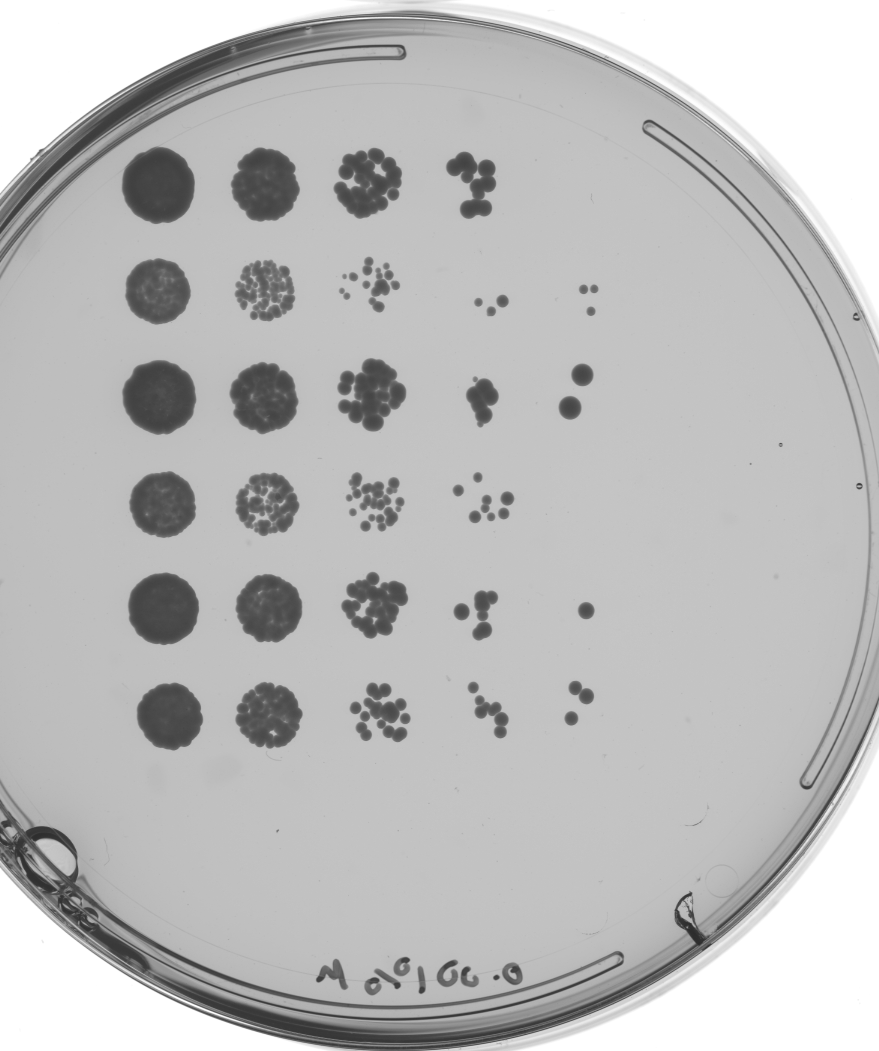

Supplement: Supplementary file 8 — EV Figure Source Data [file 44318_2025_580_MOESM8_ESM.zip › SD EV figures/Figure EV1/D/-IAA MMS 001.tif]

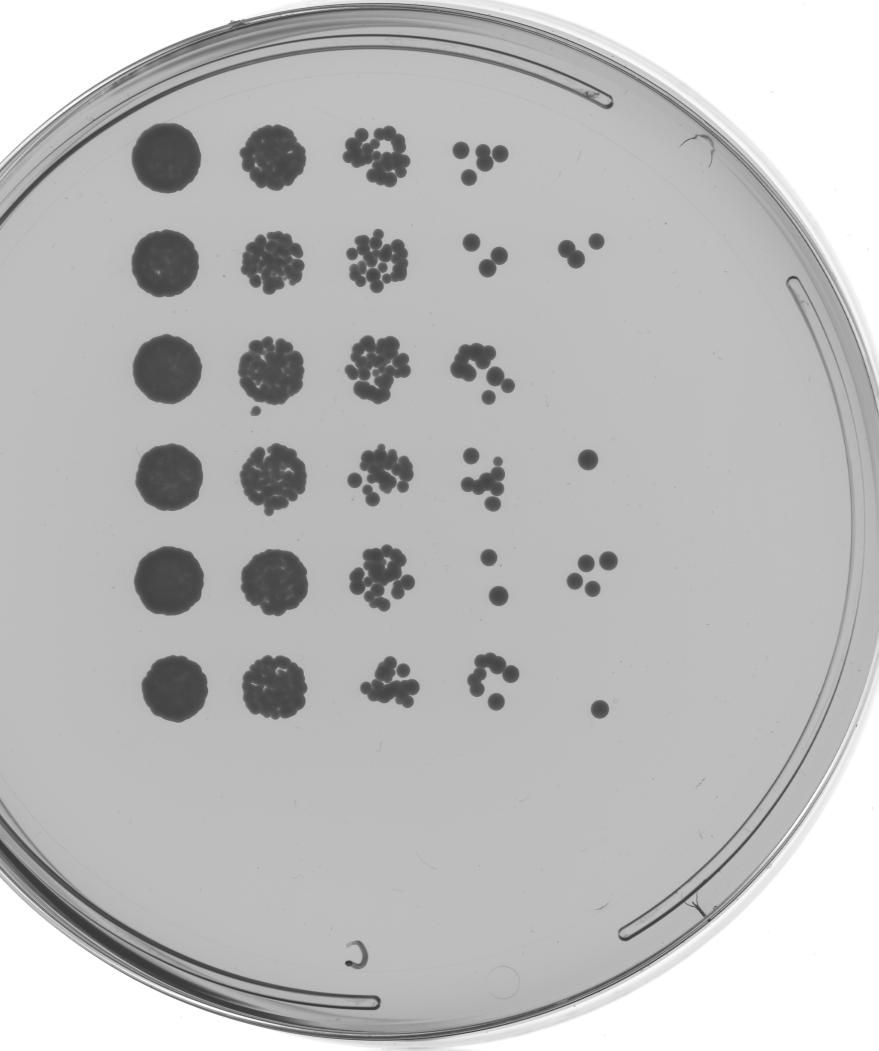

Supplement: Supplementary file 8 — EV Figure Source Data [file 44318_2025_580_MOESM8_ESM.zip › SD EV figures/Figure EV1/D/-IAA untreated.tif]

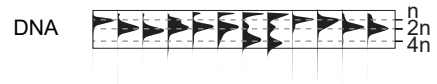

Supplement: Supplementary file 8 — EV Figure Source Data [file 44318_2025_580_MOESM8_ESM.zip › SD EV figures/Figure EV2/B/FACS/Figure EV2 B FACS.pdf]

NI-NTA  
pulldown

Ub

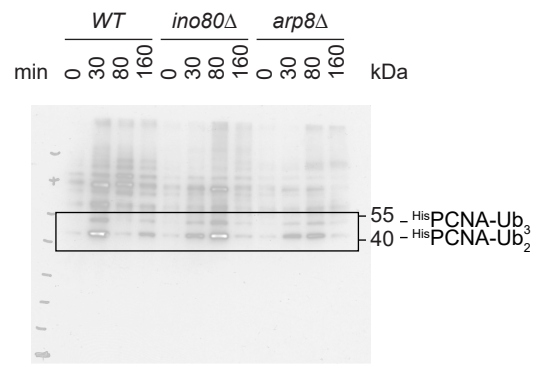

PCNA

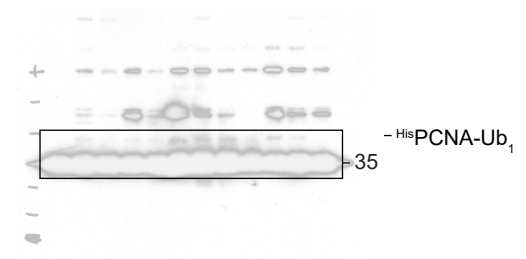

input

Rad53

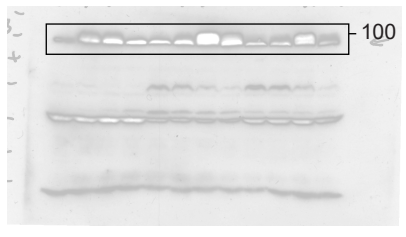

Pgk1

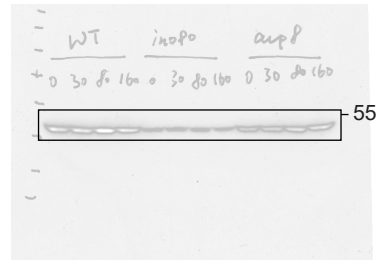

Supplement: Supplementary file 8 — EV Figure Source Data [file 44318_2025_580_MOESM8_ESM.zip › SD EV figures/Figure EV2/B/Western/Figure EV2B Western.pdf]

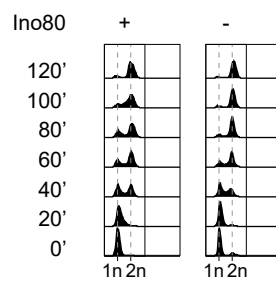

Supplement: Supplementary file 8 — EV Figure Source Data [file 44318_2025_580_MOESM8_ESM.zip › SD EV figures/Figure EV2/D/FigEV2D_FACS.pdf]

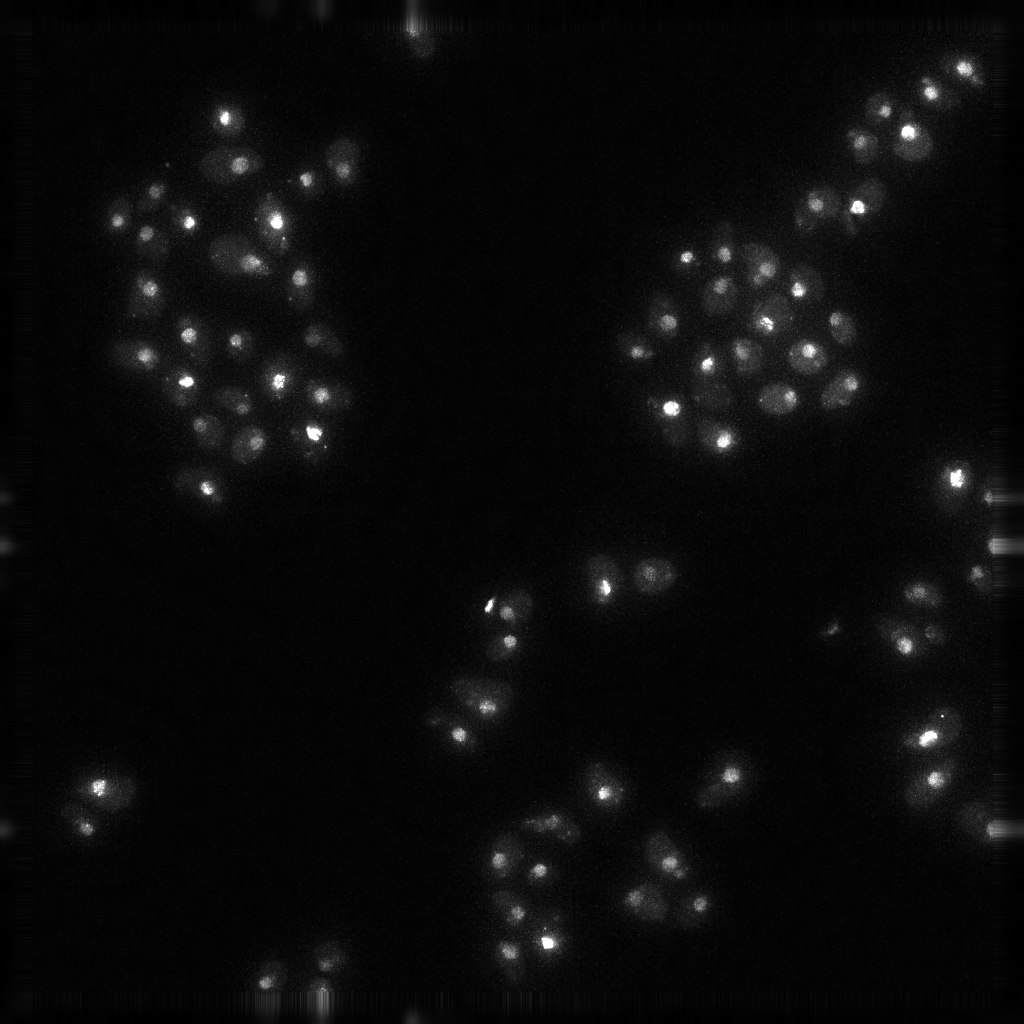

Supplement: Supplementary file 8 — EV Figure Source Data [file 44318_2025_580_MOESM8_ESM.zip › SD EV figures/Figure EV2/E/+Ino80_0.tif]

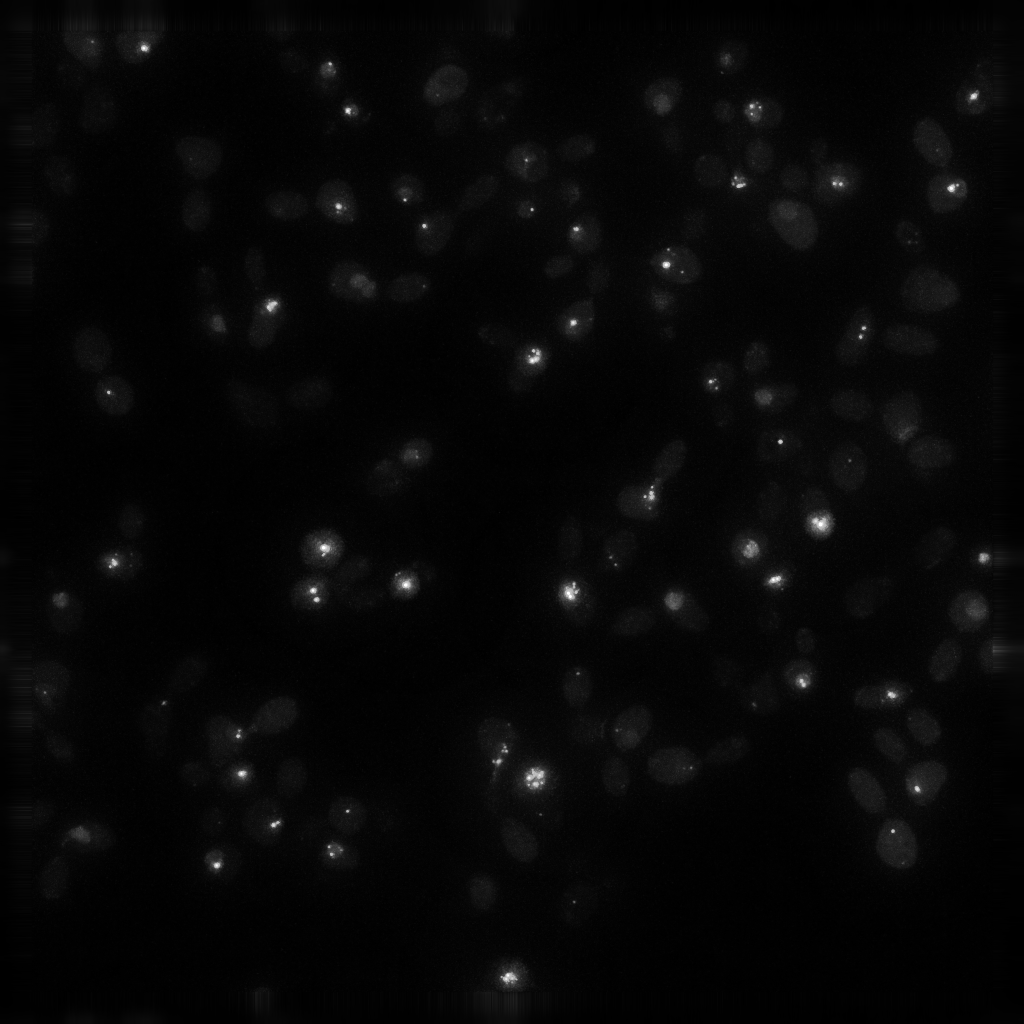

Supplement: Supplementary file 8 — EV Figure Source Data [file 44318_2025_580_MOESM8_ESM.zip › SD EV figures/Figure EV2/E/+Ino80_120.tif]

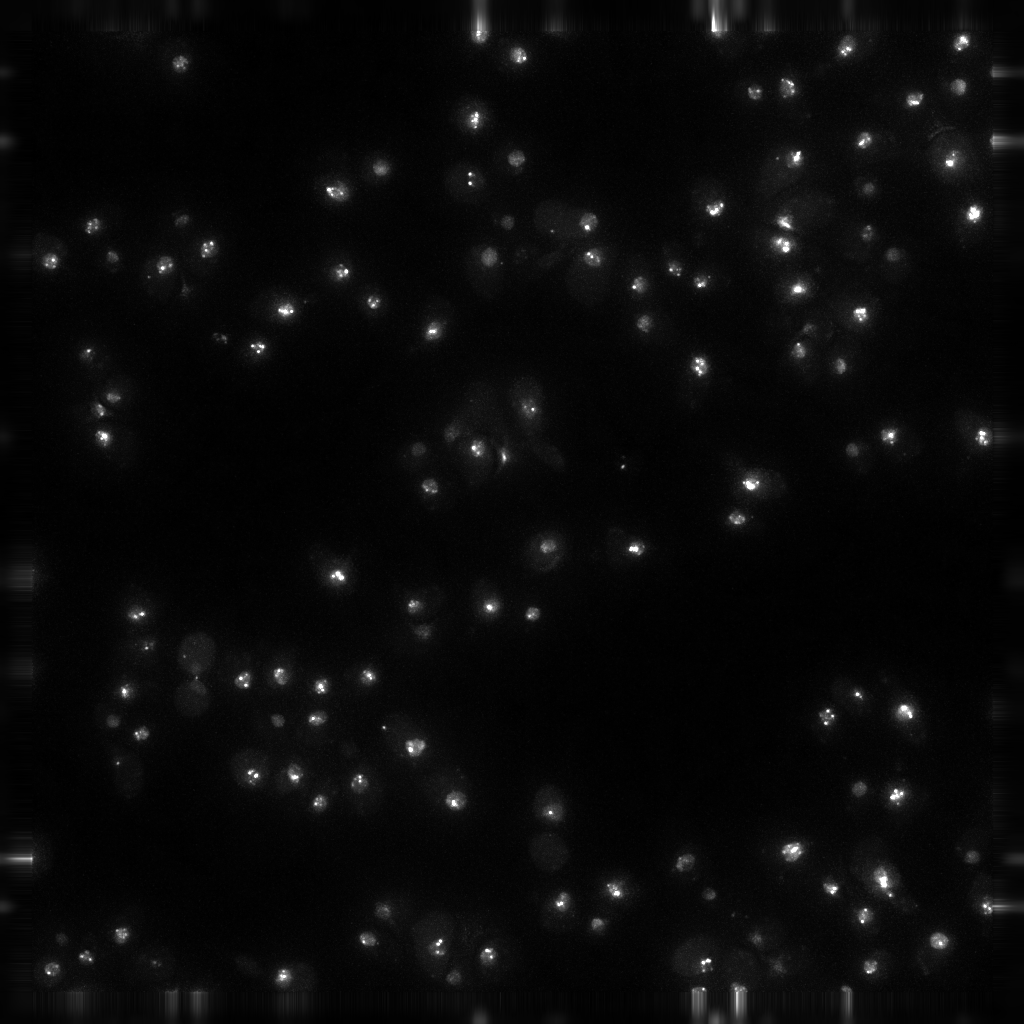

Supplement: Supplementary file 8 — EV Figure Source Data [file 44318_2025_580_MOESM8_ESM.zip › SD EV figures/Figure EV2/E/+Ino80_45.tif]

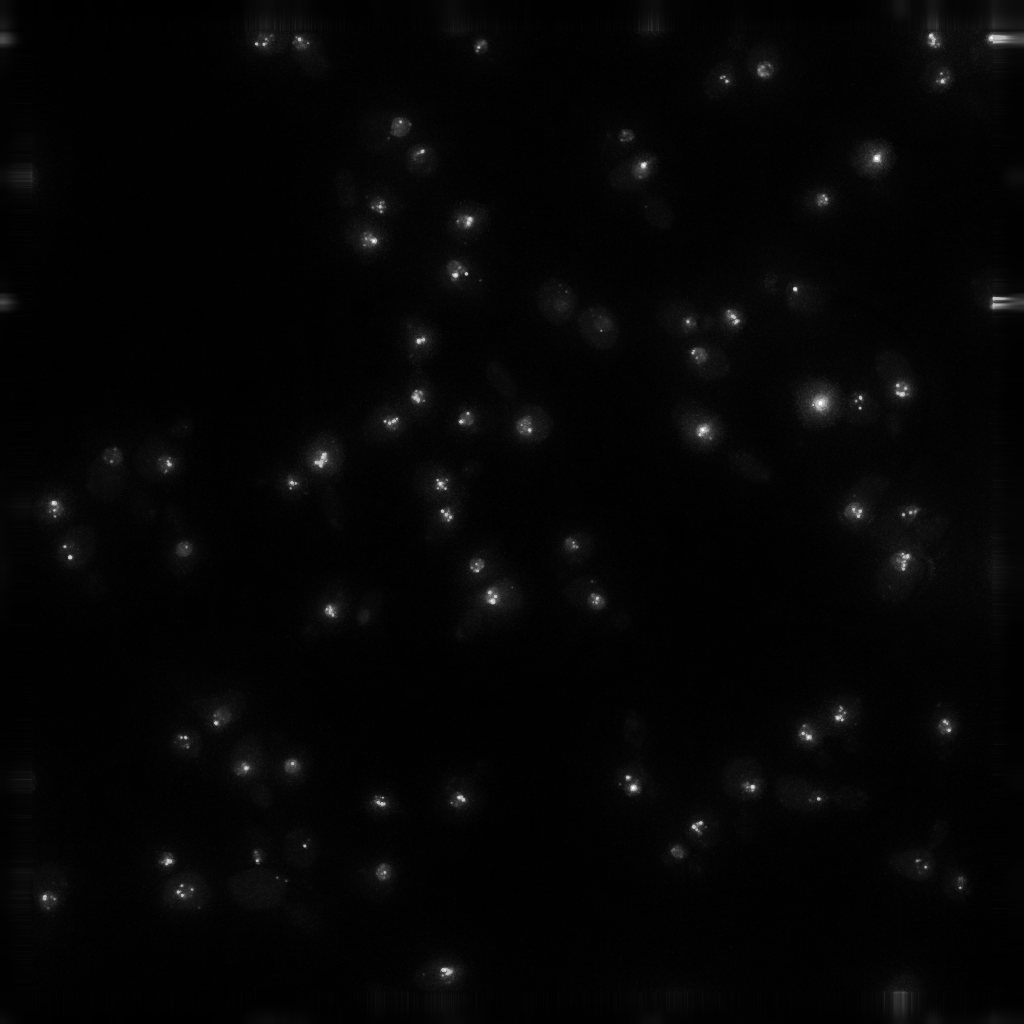

Supplement: Supplementary file 8 — EV Figure Source Data [file 44318_2025_580_MOESM8_ESM.zip › SD EV figures/Figure EV2/E/+Ino80_75.tif]

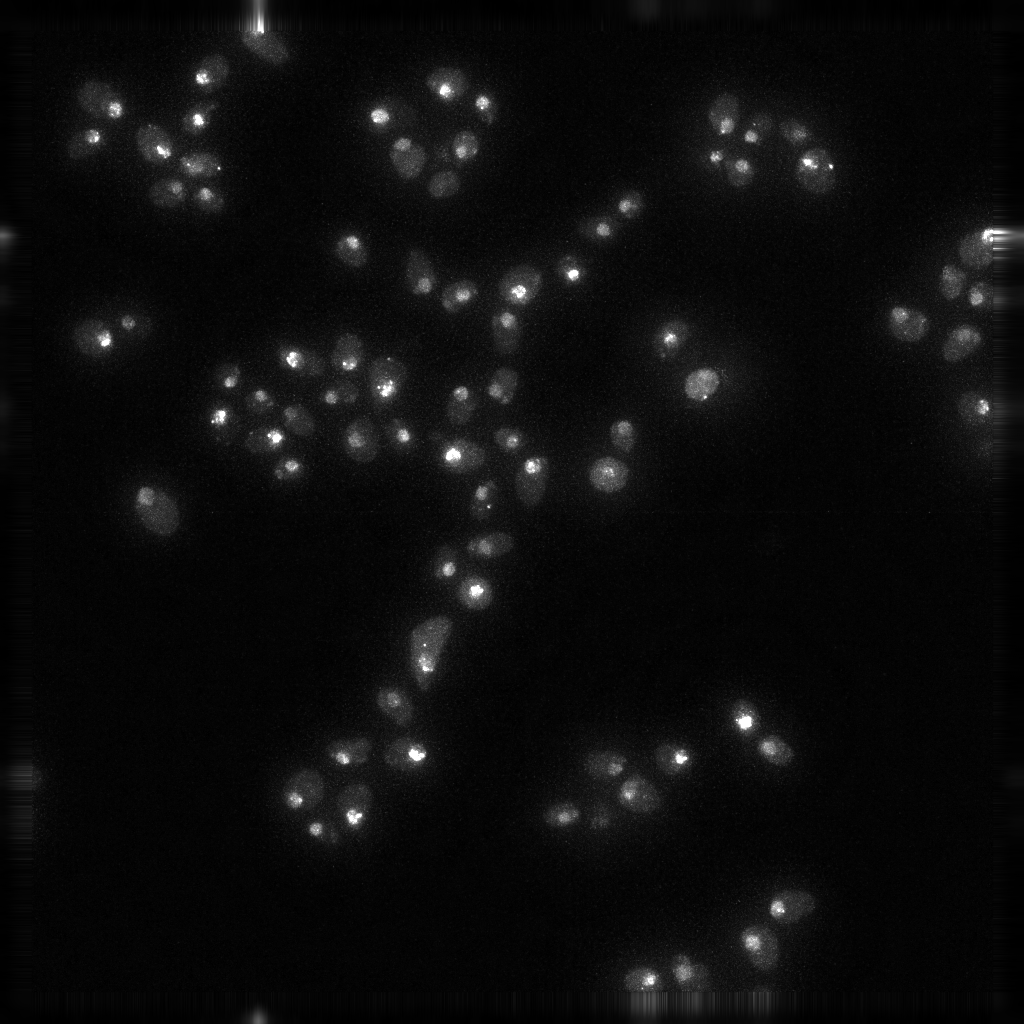

Supplement: Supplementary file 8 — EV Figure Source Data [file 44318_2025_580_MOESM8_ESM.zip › SD EV figures/Figure EV2/E/-Ino80_0.tif]

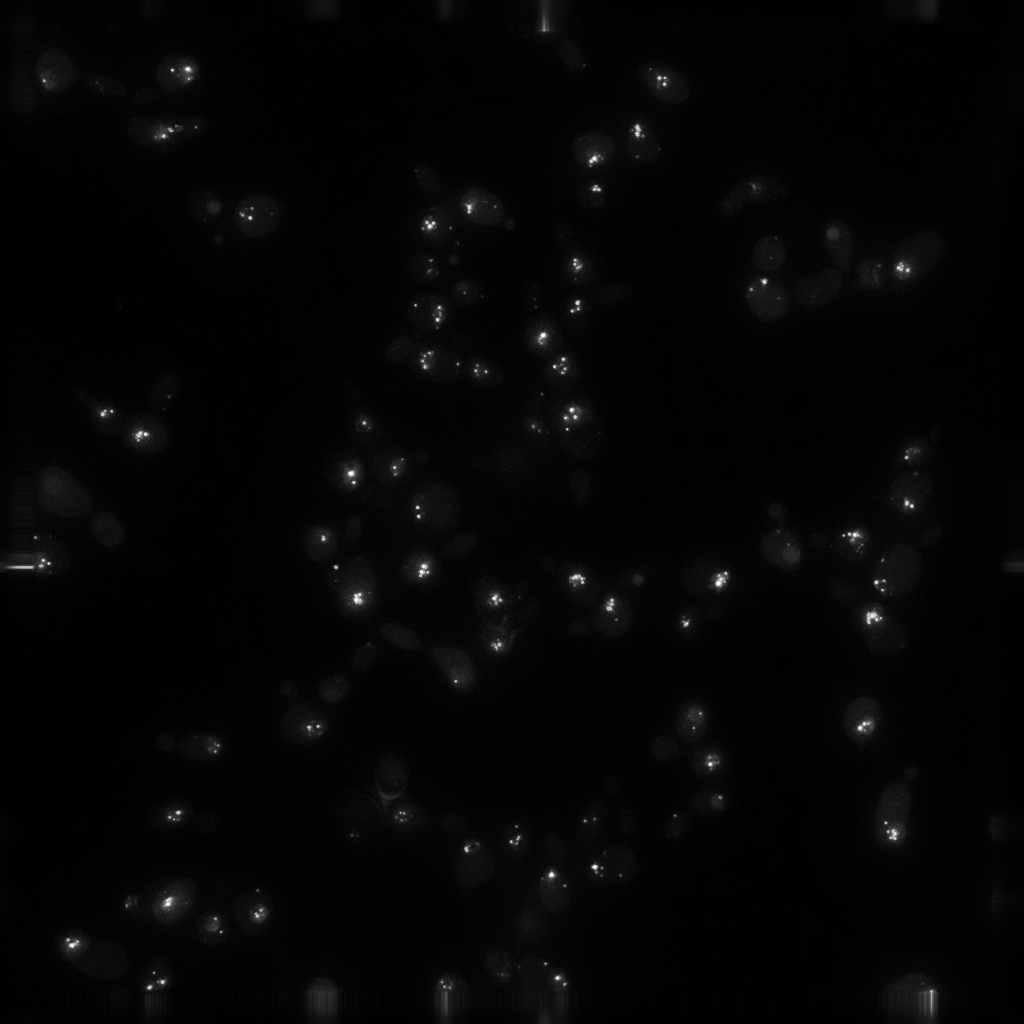

Supplement: Supplementary file 8 — EV Figure Source Data [file 44318_2025_580_MOESM8_ESM.zip › SD EV figures/Figure EV2/E/-Ino80_120.tif]

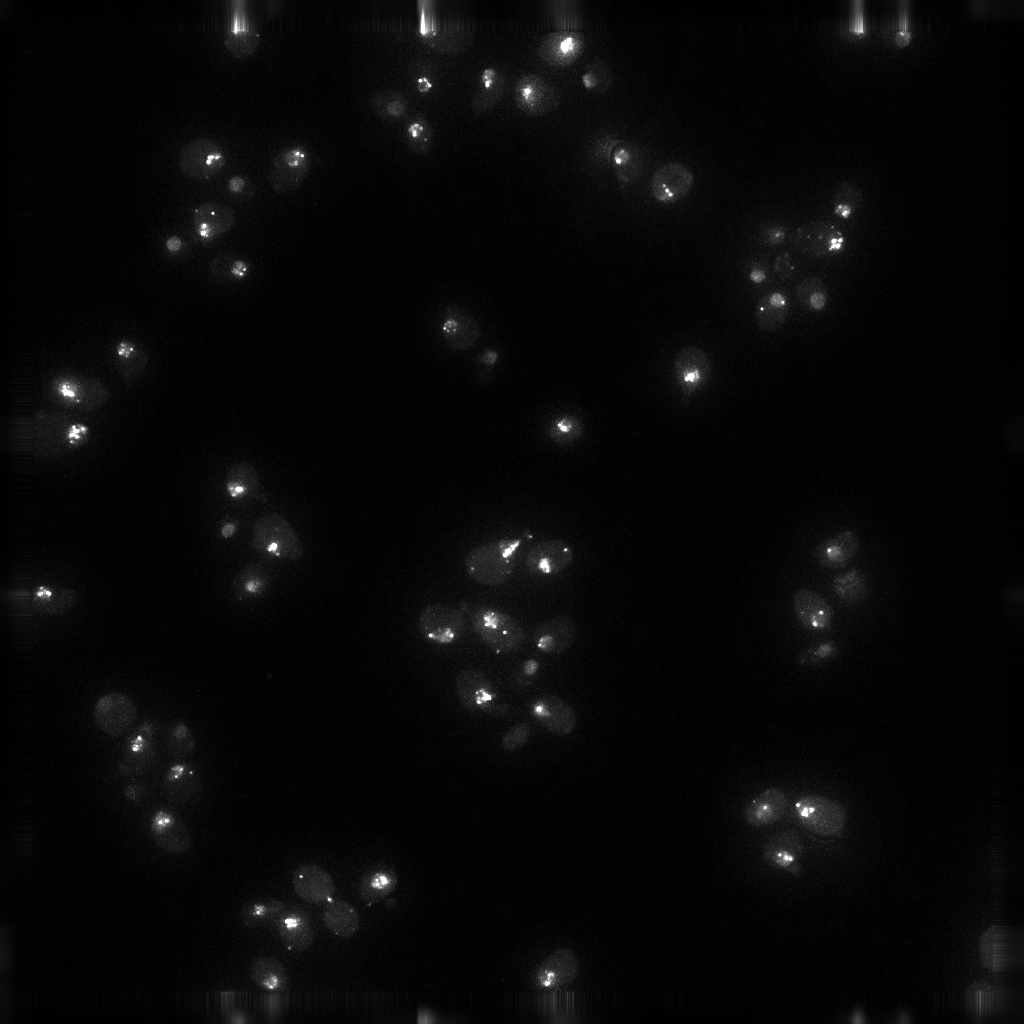

Supplement: Supplementary file 8 — EV Figure Source Data [file 44318_2025_580_MOESM8_ESM.zip › SD EV figures/Figure EV2/E/-Ino80_45.tif]

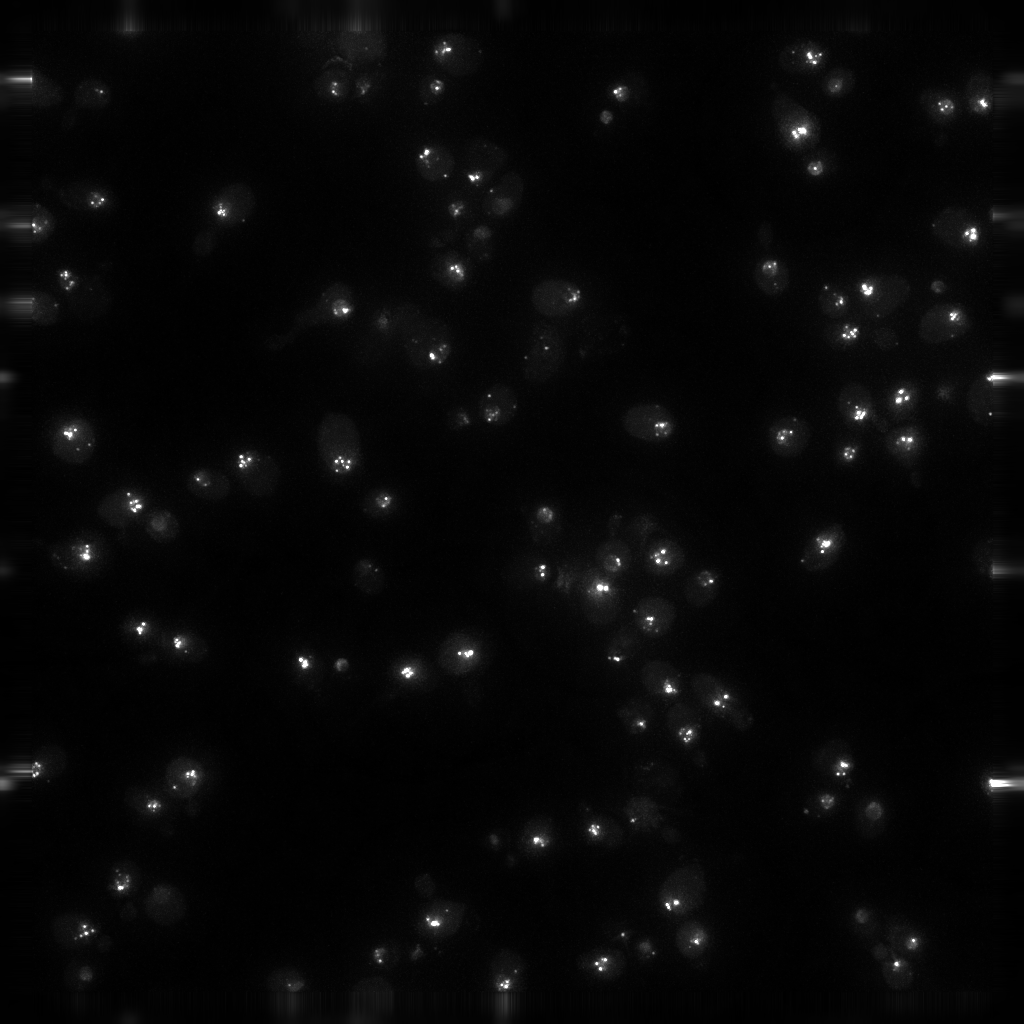

Supplement: Supplementary file 8 — EV Figure Source Data [file 44318_2025_580_MOESM8_ESM.zip › SD EV figures/Figure EV2/E/-Ino80_75.tif]

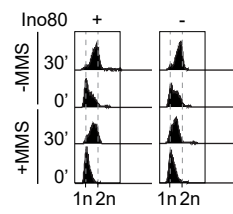

Supplement: Supplementary file 8 — EV Figure Source Data [file 44318_2025_580_MOESM8_ESM.zip › SD EV figures/Figure EV3/A/Figure EV3A_FACS.pdf]

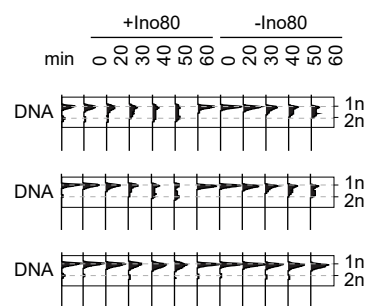

Supplement: Supplementary file 8 — EV Figure Source Data [file 44318_2025_580_MOESM8_ESM.zip › SD EV figures/Figure EV3/D/FACS/Figure EV3D_FACS.pdf]

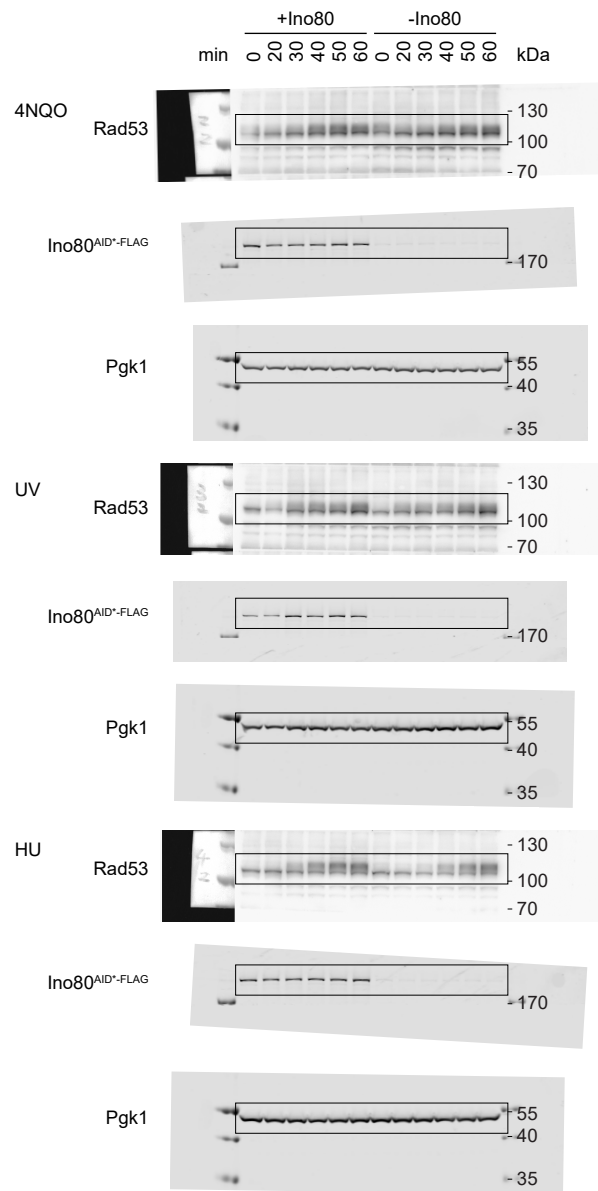

Supplement: Supplementary file 8 — EV Figure Source Data [file 44318_2025_580_MOESM8_ESM.zip › SD EV figures/Figure EV3/D/Western/Figure EV3D_Western.pdf]

|                 | G1                                                                                  |                                                                                     | G2                                                                                  |                                                                                     |                                                                                     |                                                                                     |                                                                                     |                                                                                     |                                                                                     |                                                                                      |         |
|-----------------|-------------------------------------------------------------------------------------|-------------------------------------------------------------------------------------|-------------------------------------------------------------------------------------|-------------------------------------------------------------------------------------|-------------------------------------------------------------------------------------|-------------------------------------------------------------------------------------|-------------------------------------------------------------------------------------|-------------------------------------------------------------------------------------|-------------------------------------------------------------------------------------|--------------------------------------------------------------------------------------|---------|
|                 |                                                                                     |                                                                                     | +Ino80                                                                              |                                                                                     |                                                                                     |                                                                                     | -Ino80                                                                              |                                                                                     |                                                                                     |                                                                                      |         |
| +Rad18<br>(min) | -                                                                                   | -                                                                                   | 0                                                                                   | 0                                                                                   | 60                                                                                  | 120                                                                                 | 0                                                                                   | 0                                                                                   | 60                                                                                  | 120                                                                                  |         |
| DNA             | 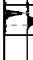 | 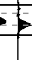 | 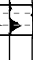 | 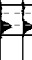 | 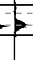 | 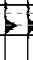 | 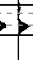 | 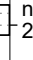 | 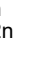 | 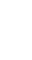 | n<br>2n |

Supplement: Supplementary file 8 — EV Figure Source Data [file 44318_2025_580_MOESM8_ESM.zip › SD EV figures/Figure EV3/G/FACS/FigureEV3G_FACS.pdf]

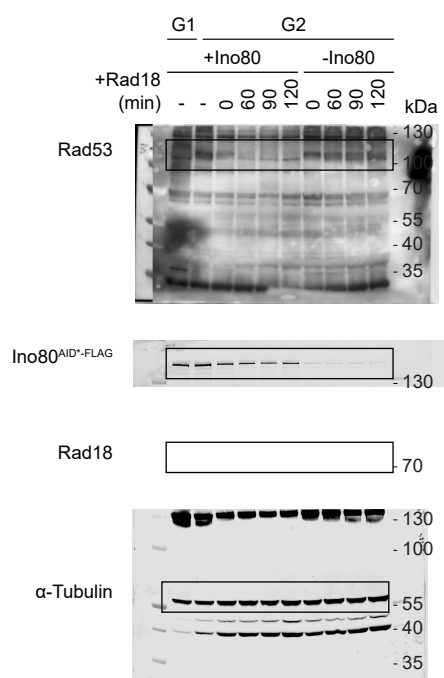

Supplement: Supplementary file 8 — EV Figure Source Data [file 44318_2025_580_MOESM8_ESM.zip › SD EV figures/Figure EV3/G/FigureEV3G_Western.pdf]

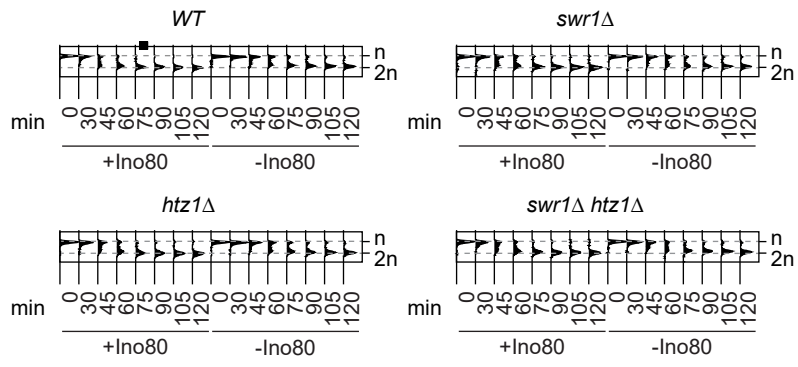

Supplement: Supplementary file 8 — EV Figure Source Data [file 44318_2025_580_MOESM8_ESM.zip › SD EV figures/Figure EV4/A/FACS/FigureEV4A_FACS.pdf]

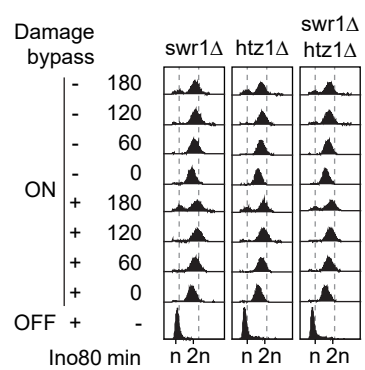

Supplement: Supplementary file 8 — EV Figure Source Data [file 44318_2025_580_MOESM8_ESM.zip › SD EV figures/Figure EV4/B/FACS/FigureEV4B_FACS.pdf]

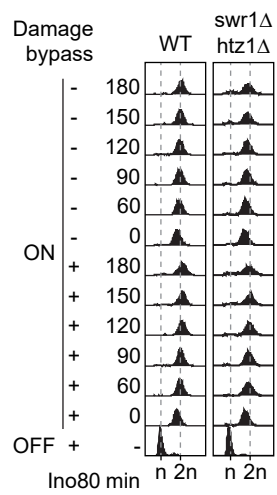

Supplement: Supplementary file 8 — EV Figure Source Data [file 44318_2025_580_MOESM8_ESM.zip › SD EV figures/Figure EV4/C/FACS/FigureEV4C_FACS.pdf]

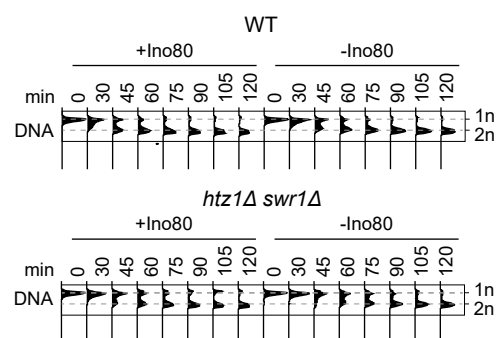

Supplement: Supplementary file 8 — EV Figure Source Data [file 44318_2025_580_MOESM8_ESM.zip › SD EV figures/Figure EV4/D/FACS/FigEV4D_FACS.pdf]

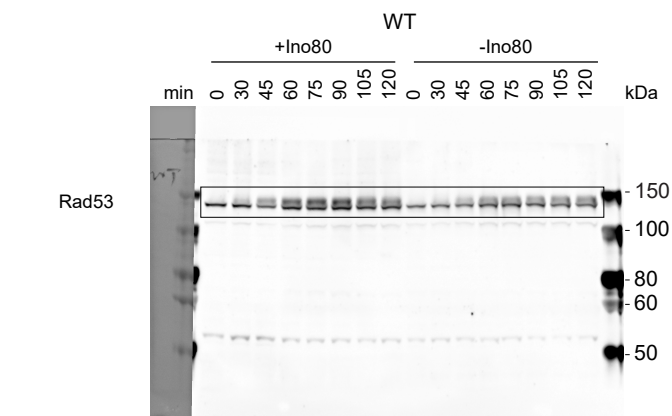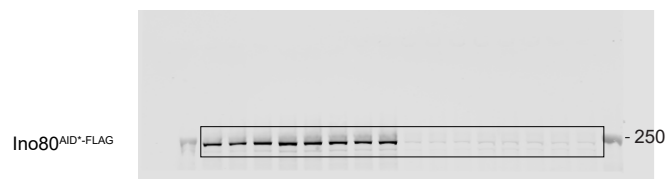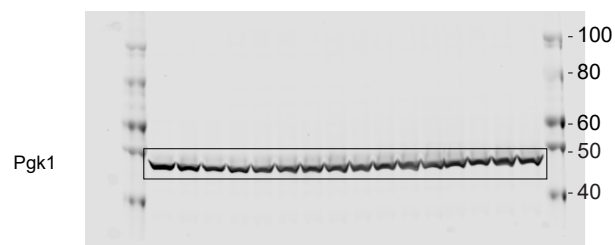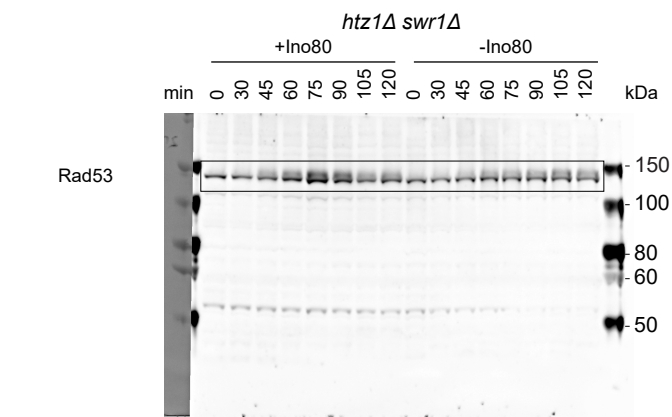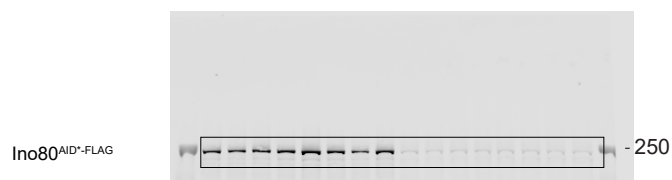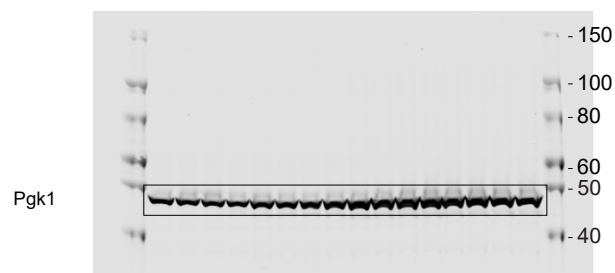

Supplement: Supplementary file 8 — EV Figure Source Data [file 44318_2025_580_MOESM8_ESM.zip › SD EV figures/Figure EV4/D/Western/FigEV4E_Western.pdf]

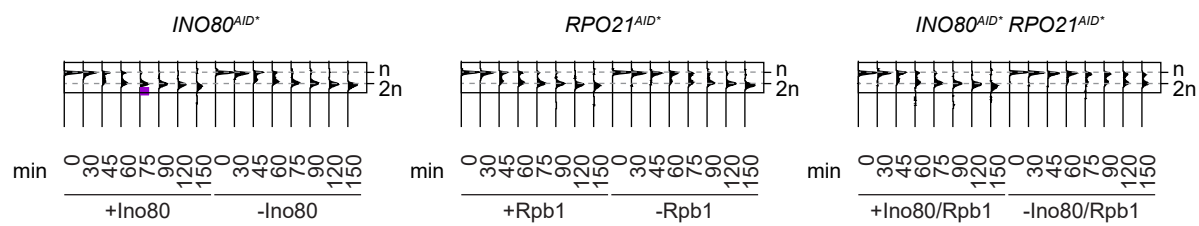

Supplement: Supplementary file 8 — EV Figure Source Data [file 44318_2025_580_MOESM8_ESM.zip › SD EV figures/Figure EV4/E/FACS/FigureEV4E_FACS.pdf]

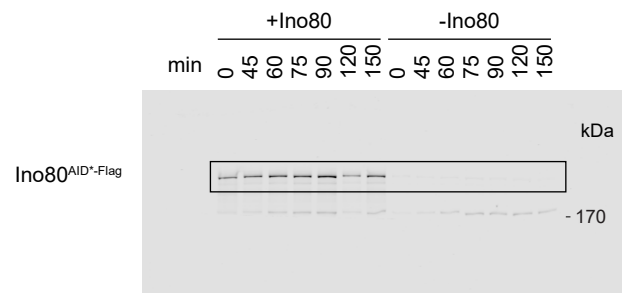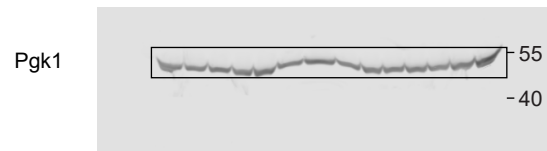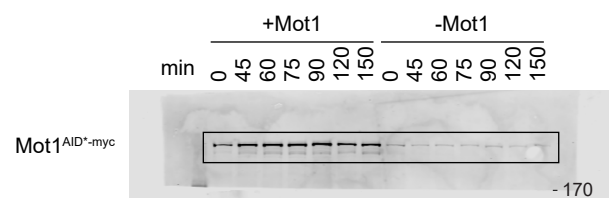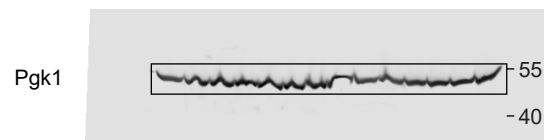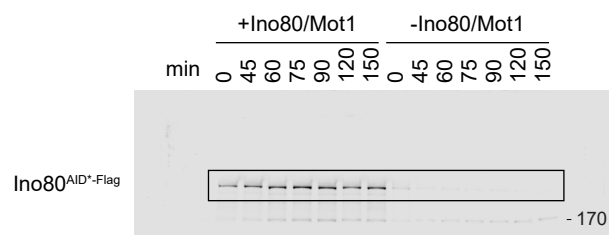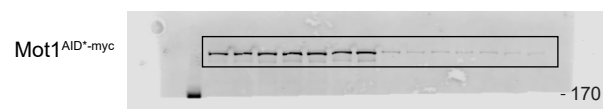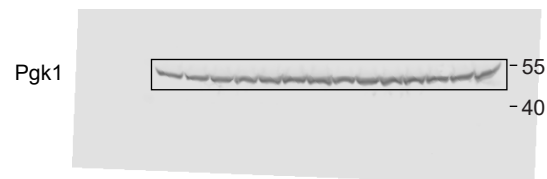

Supplement: Supplementary file 8 — EV Figure Source Data [file 44318_2025_580_MOESM8_ESM.zip › SD EV figures/Figure EV4/F/FigureEV4F_Western.pdf]

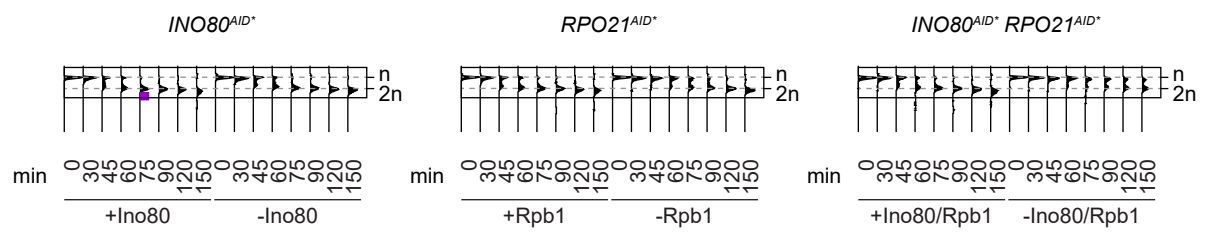

Supplement: Supplementary file 8 — EV Figure Source Data [file 44318_2025_580_MOESM8_ESM.zip › SD EV figures/Figure EV4/G/FACS/FigureEV4G_FACS.pdf]

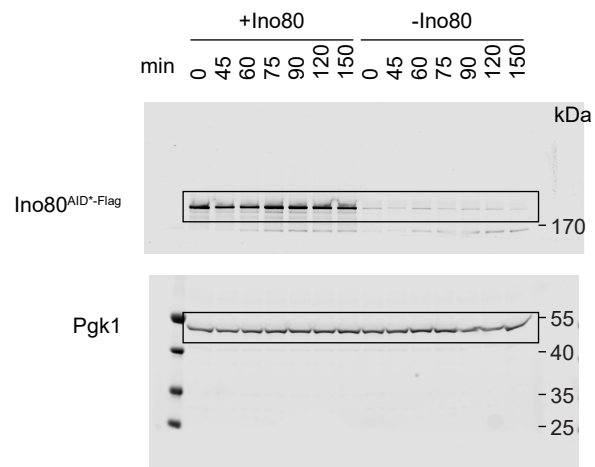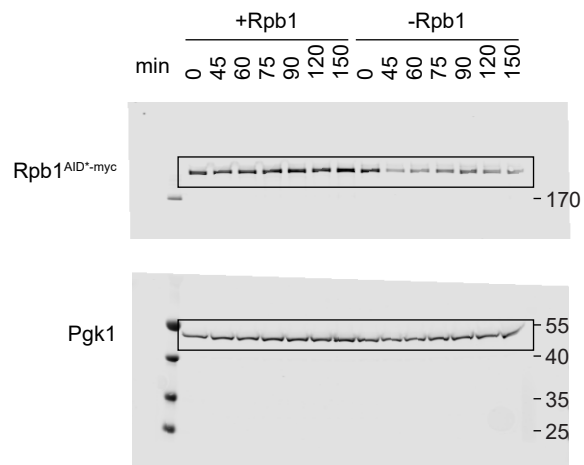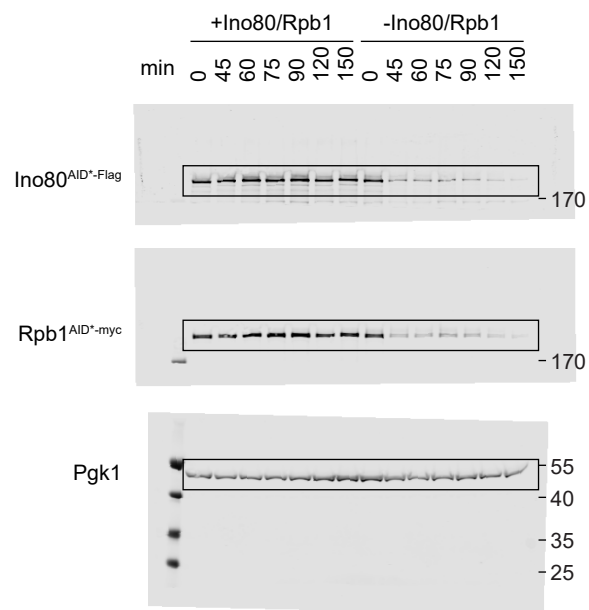

Supplement: Supplementary file 8 — EV Figure Source Data [file 44318_2025_580_MOESM8_ESM.zip › SD EV figures/Figure EV4/H/FigureEV4H_Western.pdf]
