# Supplementary material for: The INO80 chromatin remodeller facilitates DNA damage bypass via postreplicative gap repair
Source: EMBO J. 2025 Oct 13;44(22):6626–48. doi: 10.1038/s44318-025-00580-4 (PMC12624141; doi:10.1038/s44318-025-00580-4)
Supplement: Supplementary file 9 — Expanded View Figures [file 44318_2025_580_MOESM9_ESM.pdf]

# Expanded View Figures

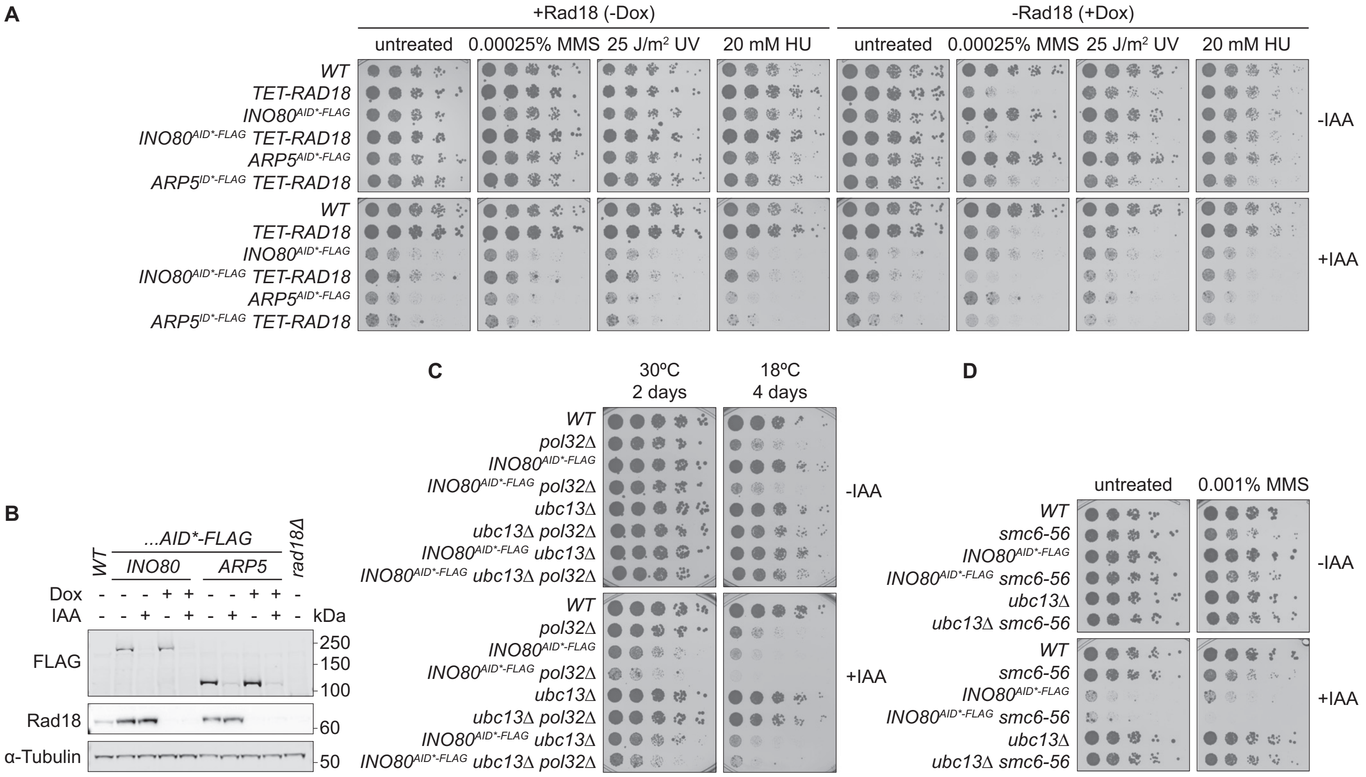


**Figure EV1. The INO80 complex contributes to *RAD6*-independent pathways of damage resistance.**

(**A**) Depletion of Ino80 or Arp5 via an auxin-inducible degron (AID*-FLAG) aggravates the DNA damage sensitivity induced by doxycycline-mediated repression of *RAD18* expression in the *TET-RAD18* strain background (Dox doxycycline, IAA auxin; biological replicates: *N* = 1). (**B**) Regulation of Ino80$^{AID*-FLAG}$, Arp5$^{AID*-FLAG}$ and Rad18 protein levels by auxin and doxycycline. Proteins were detected by western blotting in the strains used for the experiment shown in panel (**A**). A blot against α-tubulin served as a loading control (biological replicates: *N* = 1). (**C**, **D**) *INO80* does not act specifically in the TS pathway. Suppression of the cold sensitivity of *pol32Δ* was assessed upon auxin-mediated depletion of Ino80$^{AID*-FLAG}$ at the indicated temperatures (**C**; biological replicates: *N* = 2), and suppression of the damage sensitivity of *smc6-56* was assessed for mutant combinations by growth in the presence of MMS (**D**; biological replicates: *N* = 1). A *ubc13Δ* mutant served as a control for a TS-specific defect. Source data are available online for this figure.

 © The Author(s)

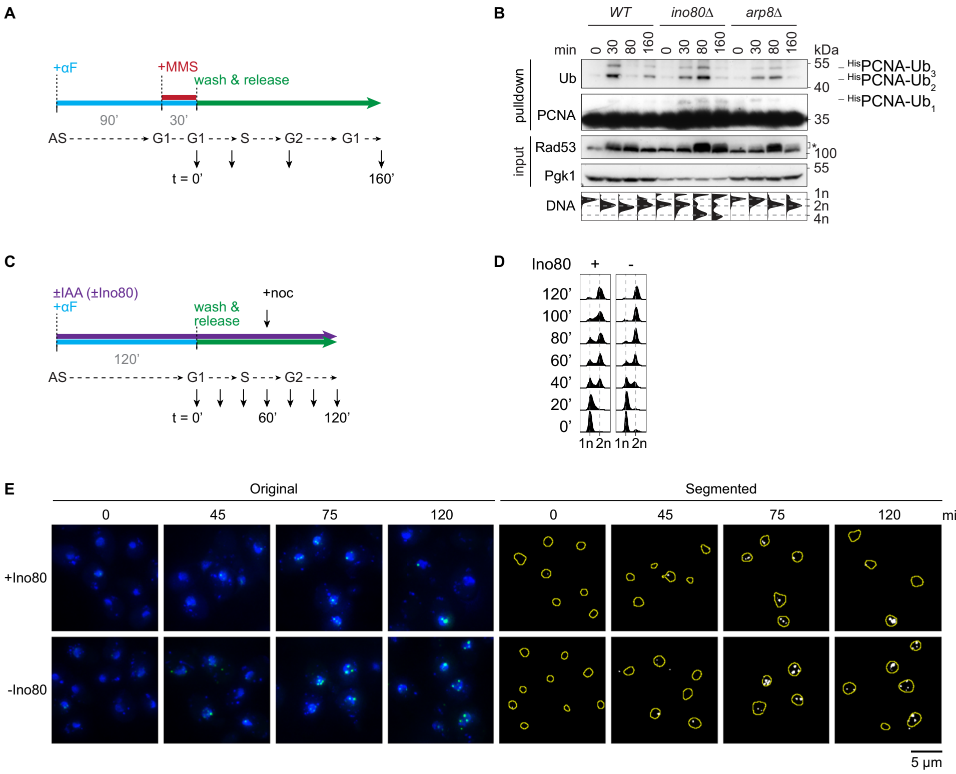


**Figure EV2. The INO80 complex prevents accumulation of ssDNA during replication of damaged DNA.**

(**A**) Experimental scheme to assess damage-induced PCNA ubiquitylation along S phase. Cells are synchronised in G1 phase and treated with 0.02% MMS for 30 min prior to release into S phase and collection of samples. (**B**) DNA damage-induced PCNA ubiquitylation is delayed but not reduced in the absence of functional INO80 complex. Modified PCNA was detected by Ni-NTA pulldown in lysates prepared under denaturing conditions from strains of the indicated genotypes harbouring a $His_6$-tagged allele of PCNA (${}^{His}$*POL30*). Rad53 phosphorylation (*) was detected in total lysates as a marker of checkpoint activation, and Pgk1 served as a loading control. Corresponding cell cycle profiles are shown below the blots (biological replicates: $N = 1$). (**C**) Experimental scheme to assess cell cycle progression upon depletion of Ino80. Cells harbouring $INO80^{AID*\text{-}FLAG}$ are synchronised in G1 in the presence or absence of auxin (IAA, 0.5 mM), washed and released into YPD medium. Nocodazole (noc) is added 60 min after release to prevent transition to the next cell cycle. Samples are collected for flow cytometry at the indicated time points. (**D**) Depletion of Ino80 causes minimal delay in cell cycle progression in the absence of DNA damage. Cell cycle profiles were determined by flow cytometry from samples treated as shown in panel (**C**) (biological replicates: $N = 1$). (**E**) An example of foci segmentation. Representative images of RPA foci from Fig. 2D, sampled during the course of an S phase in the presence or absence of Ino80, were segmented according to the procedure described in the Methods section (white: segmented foci; yellow: outline of nuclei). Source data are available online for this figure.

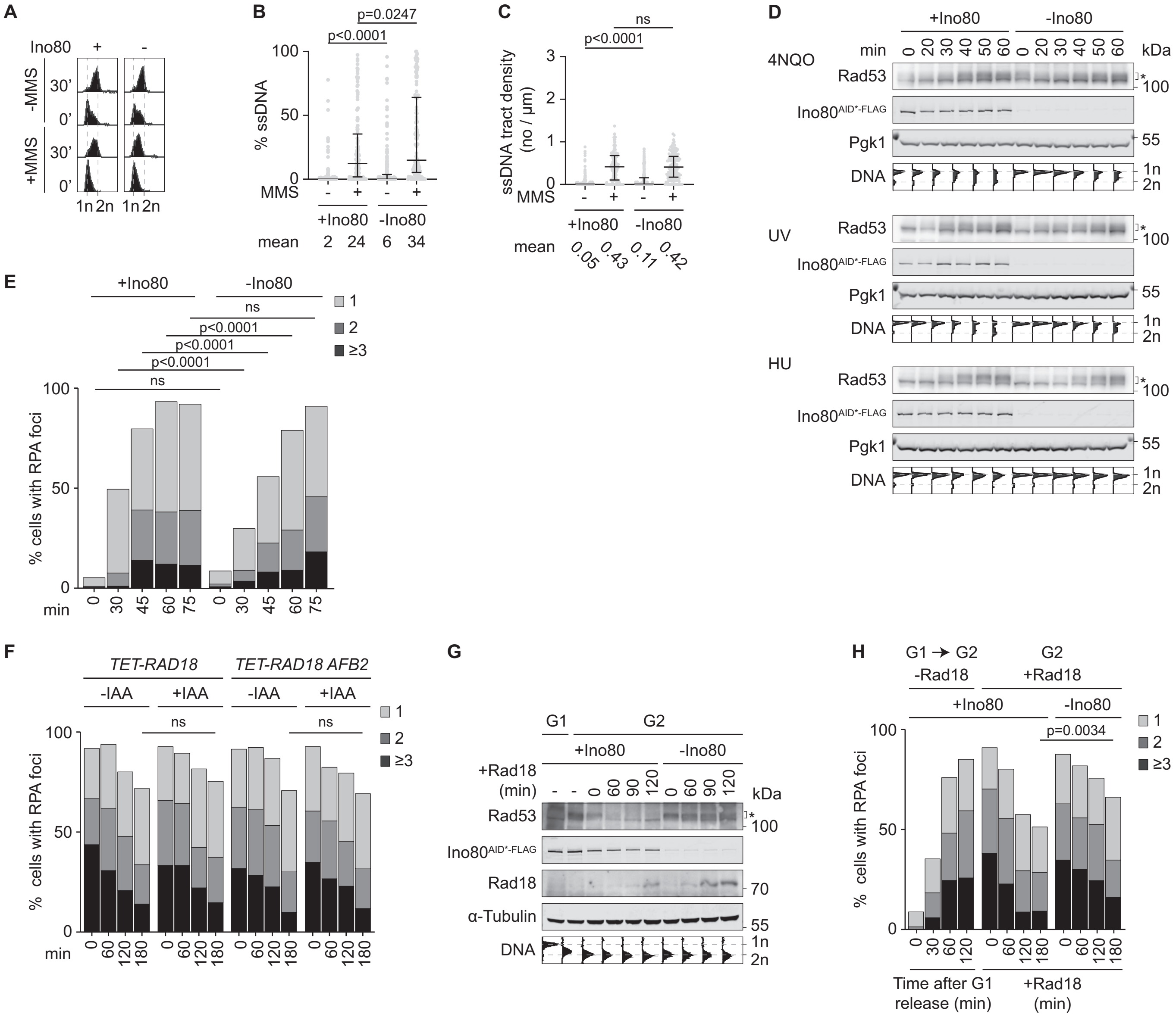


**Figure EV3. The INO80 complex facilitates both DNA resection and daughter-strand gap filling.**

(**A–C**) An independent biological replicate of the experiment shown in Fig. 3B–D (Mann–Whitney *U*-test; ns not significant; biological replicates: $N = 2$). Bars indicate median with interquartile ranges. (**D**) Depletion of Ino80 delays damage-induced checkpoint activation (*) but also S phase progression. Cells were treated as in Fig. 2A, but MMS was replaced with a 30 min treatment with 0.1 µg/ml 4-nitroquinoline oxide (4NQO) or a pulse of 20 J/m$^2$ UV-C before release or a release into medium containing 120 mM HU (biological replicates: $N = 2$). (**E**) Percentage of cells with RPA foci in the presence and absence of Ino80, corresponding to the experiment shown in Fig. 3G (Mann–Whitney *U*-test; ns not significant; biological replicates: $N = 2$). (**F**) Auxin and the auxin-responsive F-box protein Afb2 do not affect DDT-dependent resolution of RPA foci in G2/M. Gap-filling assays were performed as in Fig. 3M in the strains *TET-RAD18 RFA1$^{GFP}$* and *TET-RAD18 AFB2 RFA1$^{GFP}$* (Mann–Whitney *U*-test; ns not significant; biological replicates: $N = 1$). (**G**) An independent biological replicate of the experiment shown in Fig. 3L (biological replicates: $N = 2$). (**H**) An independent biological replicate of the experiment shown in Fig. 3M (Mann–Whitney *U*-test; biological replicates: $N = 2$). Source data are available online for this figure.

 © The Author(s)

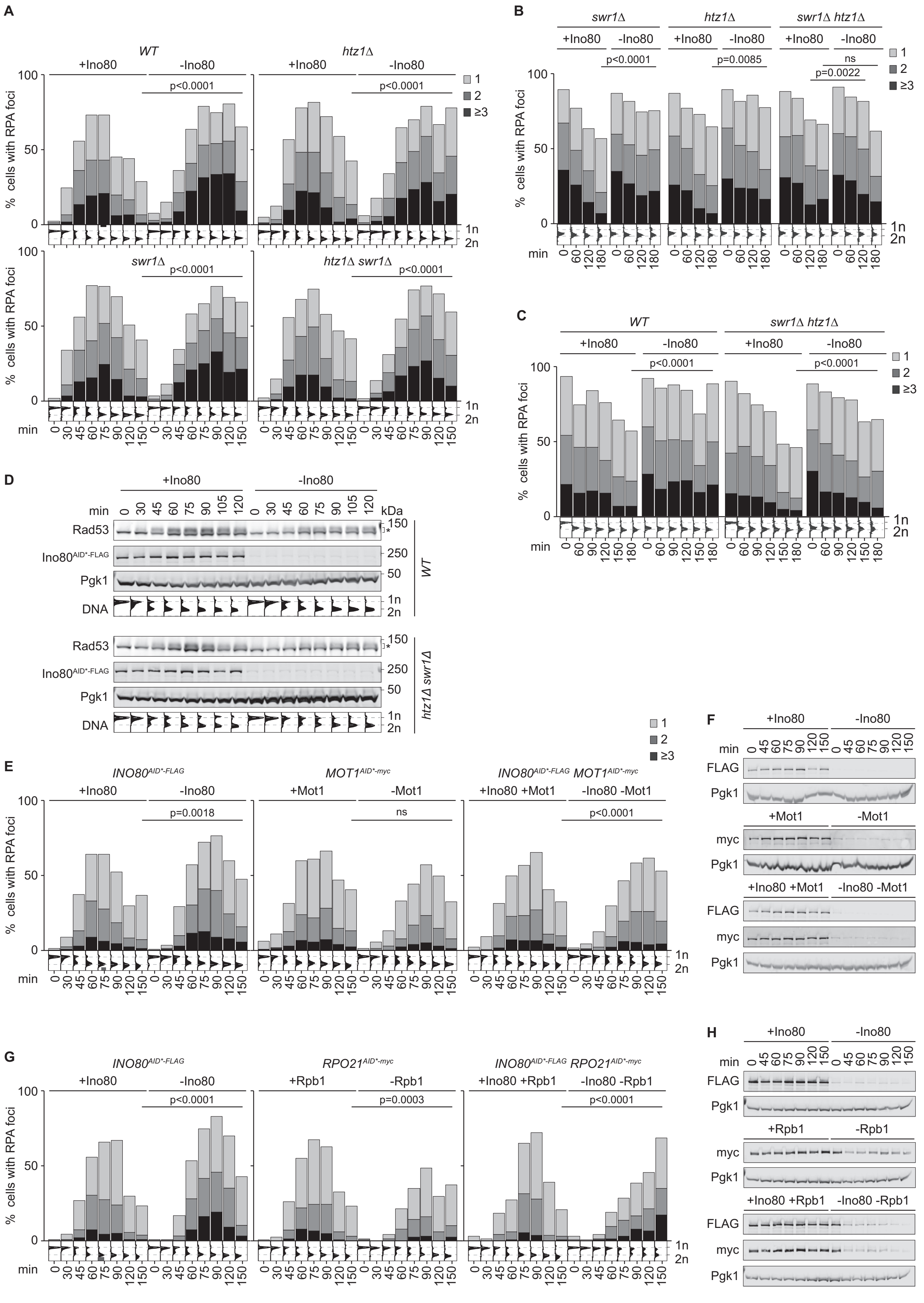
A
WT
htz1Δ
+Ino80
-Ino80
p<0.0001
swr1Δ
htz1Δ swr1Δ
% cells with RPA foci
min
1n
2n
1
2
≥3
B
swr1Δ
htz1Δ
swr1Δ htz1Δ
p=0.0085
p=0.0022
ns
C
D
Rad53
Ino80AID*-FLAG
Pgk1
DNA
kDa
E
INO80AID*-FLAG
MOT1AID*-myc
+Mot1
-Mot1
p=0.0018
F
FLAG
myc
G
RPO21AID*-myc
+Rpb1
-Rpb1
p=0.0003
H

**Figure EV4. The function of the INO80 complex in postreplicative repair is unrelated to its role in H2A.Z extraction and repression of pervasive transcription.**

(**A**) An independent biological replicate of the experiment shown in Fig. 4E (Mann–Whitney *U*-test; biological replicates: $N = 2$). (**B–C**) Two independent biological replicates of the experiment shown in Fig. 4F (Mann–Whitney *U*-test; ns not significant; biological replicates: $N = 3$). (**D**) Deletion of *HTZ1* and *SWR1* does not suppress the prolonged checkpoint activation observed upon depletion of Ino80. *WT* and *htz1 swr1* mutants were treated as in Fig. 2A, and checkpoint activation was monitored via Rad53 phosphorylation (*). Cell cycle profiles are shown below the blots (biological replicates: $N = 1$). (**E**) Depletion of Mot1 causes cell cycle delays and interferes with the replication-associated emergence of RPA foci. RPA foci were monitored in the indicated strain backgrounds according to the scheme in Fig. 2A, except that AID*-tagged proteins were transiently degraded for 45 min before release into S phase (Mann–Whitney *U*-test; ns not significant; biological replicates: $N = 2$). Cell cycle profiles are shown below the graphs. (**F**) Western blots corresponding to the experiment shown in panel (**E**), demonstrating efficient depletion of Ino80 and Mot1 (biological replicates: $N = 2$). (**G**) Depletion of the large subunit of RNA polymerase II, Rpb1 (encoded by *RPO21*), delays replication but does not suppress the defect in the resolution of replication-associated RPA foci in the absence of Ino80. RPA foci were monitored in the indicated strain backgrounds according to the scheme in Fig. 2A, except that AID*-tagged proteins were transiently degraded for 45 min before release into S phase (Mann–Whitney *U*-test; biological replicates: $N = 1$). Cell cycle profiles are shown below the graphs. (**H**) Western blots corresponding to the experiment shown in panel (**G**), demonstrating efficient depletion of Ino80 and Rpb1 (biological replicates: $N = 1$). Source data are available online for this figure.

 © The Author(s)

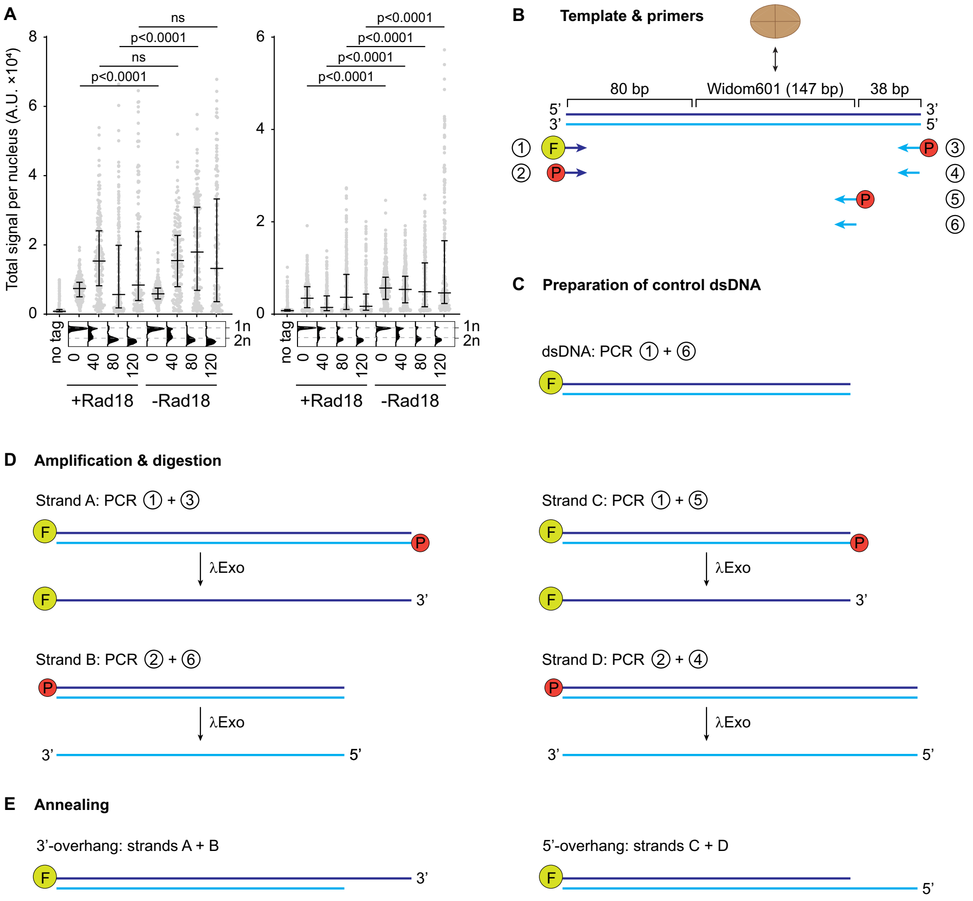


**Figure EV5. Construction of daughter-strand gap mimics for in vitro nucleosome sliding assays.**

(**A**) Two independent biological replicates of the proximity ligation assay shown in Fig. 5B (Mann–Whitney *U*-test; ns not significant; biological replicates: $N = 3$). Bars indicate median with interquartile ranges. (**B**) Template DNA for the preparation of substrates for nucleosome repositioning assays and oligonucleotides used for amplification of the template (P: 5′-phosphoryl; F: 5′-Fluoro-dT). (**C**) Preparation of control dsDNA substrate. (**D**, **E**) Preparation of substrates with 38 nucleotide overhangs requires isolation of individual strands (**D**) and annealing of single-stranded products in the desired combinations (**E**). PCR products produced with the indicated primer pairs were subjected to Lambda exonuclease (λExo) digestion to selectively degrade the 5′-phosphorylated strand. Source data are available online for this figure.

© The Author(s)
